# Chromosome-scale *Elaeis guineensis* and *E. oleifera* assemblies: comparative genomics of oil palm and other Arecaceae

**DOI:** 10.1093/g3journal/jkae135

**Published:** 2024-06-26

**Authors:** Eng-Ti Leslie Low, Kuang-Lim Chan, Noorhariza Mohd Zaki, Elizaveta Taranenko, Jared M Ordway, Corey Wischmeyer, Jaap Buntjer, Mohd Amin Ab Halim, Nik Shazana Nik Mohd Sanusi, Jayanthi Nagappan, Rozana Rosli, Eugeniya Bondar, Nadzirah Amiruddin, Norashikin Sarpan, Ngoot-Chin Ting, Pek-Lan Chan, Meilina Ong-Abdullah, Marhalil Marjuni, Suzana Mustaffa, Norziha Abdullah, Norazah Azizi, Blaire Bacher, Nathan Lakey, Tatiana V Tatarinova, Mohamad Arif Abd Manaf, Ravigadevi Sambanthamurti, Rajinder Singh

**Affiliations:** Advanced Biotechnology and Breeding Centre, Malaysian Palm Oil Board, 6 Persiaran Institusi, Bandar Baru Bangi, 43000 Kajang, Selangor, Malaysia; Advanced Biotechnology and Breeding Centre, Malaysian Palm Oil Board, 6 Persiaran Institusi, Bandar Baru Bangi, 43000 Kajang, Selangor, Malaysia; Advanced Biotechnology and Breeding Centre, Malaysian Palm Oil Board, 6 Persiaran Institusi, Bandar Baru Bangi, 43000 Kajang, Selangor, Malaysia; Biology Department, University of La Verne, La Verne, CA 91750, USA; Orion Genomics, 3730 Foundry Way, St. Louis, MO 63110, USA; Orion Genomics, 3730 Foundry Way, St. Louis, MO 63110, USA; Orion Genomics, 3730 Foundry Way, St. Louis, MO 63110, USA; Advanced Biotechnology and Breeding Centre, Malaysian Palm Oil Board, 6 Persiaran Institusi, Bandar Baru Bangi, 43000 Kajang, Selangor, Malaysia; Advanced Biotechnology and Breeding Centre, Malaysian Palm Oil Board, 6 Persiaran Institusi, Bandar Baru Bangi, 43000 Kajang, Selangor, Malaysia; Advanced Biotechnology and Breeding Centre, Malaysian Palm Oil Board, 6 Persiaran Institusi, Bandar Baru Bangi, 43000 Kajang, Selangor, Malaysia; Advanced Biotechnology and Breeding Centre, Malaysian Palm Oil Board, 6 Persiaran Institusi, Bandar Baru Bangi, 43000 Kajang, Selangor, Malaysia; Biology Department, University of La Verne, La Verne, CA 91750, USA; Advanced Biotechnology and Breeding Centre, Malaysian Palm Oil Board, 6 Persiaran Institusi, Bandar Baru Bangi, 43000 Kajang, Selangor, Malaysia; Advanced Biotechnology and Breeding Centre, Malaysian Palm Oil Board, 6 Persiaran Institusi, Bandar Baru Bangi, 43000 Kajang, Selangor, Malaysia; Advanced Biotechnology and Breeding Centre, Malaysian Palm Oil Board, 6 Persiaran Institusi, Bandar Baru Bangi, 43000 Kajang, Selangor, Malaysia; Advanced Biotechnology and Breeding Centre, Malaysian Palm Oil Board, 6 Persiaran Institusi, Bandar Baru Bangi, 43000 Kajang, Selangor, Malaysia; Advanced Biotechnology and Breeding Centre, Malaysian Palm Oil Board, 6 Persiaran Institusi, Bandar Baru Bangi, 43000 Kajang, Selangor, Malaysia; Advanced Biotechnology and Breeding Centre, Malaysian Palm Oil Board, 6 Persiaran Institusi, Bandar Baru Bangi, 43000 Kajang, Selangor, Malaysia; Advanced Biotechnology and Breeding Centre, Malaysian Palm Oil Board, 6 Persiaran Institusi, Bandar Baru Bangi, 43000 Kajang, Selangor, Malaysia; Advanced Biotechnology and Breeding Centre, Malaysian Palm Oil Board, 6 Persiaran Institusi, Bandar Baru Bangi, 43000 Kajang, Selangor, Malaysia; Advanced Biotechnology and Breeding Centre, Malaysian Palm Oil Board, 6 Persiaran Institusi, Bandar Baru Bangi, 43000 Kajang, Selangor, Malaysia; Orion Genomics, 3730 Foundry Way, St. Louis, MO 63110, USA; Orion Genomics, 3730 Foundry Way, St. Louis, MO 63110, USA; Biology Department, University of La Verne, La Verne, CA 91750, USA; Advanced Biotechnology and Breeding Centre, Malaysian Palm Oil Board, 6 Persiaran Institusi, Bandar Baru Bangi, 43000 Kajang, Selangor, Malaysia; Advanced Biotechnology and Breeding Centre, Malaysian Palm Oil Board, 6 Persiaran Institusi, Bandar Baru Bangi, 43000 Kajang, Selangor, Malaysia; Advanced Biotechnology and Breeding Centre, Malaysian Palm Oil Board, 6 Persiaran Institusi, Bandar Baru Bangi, 43000 Kajang, Selangor, Malaysia

**Keywords:** *Elaeis guineensis*, *Elaeis oleifera*, chromosome-scale genomes, comparative genomics

## Abstract

*Elaeis guineensis* and *E. oleifera* are the two species of oil palm. *E. guineensis* is the most widely cultivated commercial species, and introgression of desirable traits from *E. oleifera* is ongoing. We report an improved *E. guineensis* genome assembly with substantially increased continuity and completeness, as well as the first chromosome-scale *E. oleifera* genome assembly. Each assembly was obtained by integration of long-read sequencing, proximity ligation sequencing, optical mapping, and genetic mapping. High interspecific genome conservation is observed between the two species. The study provides the most extensive gene annotation to date, including 46,697 *E. guineensis* and 38,658 *E. oleifera* gene predictions. Analyses of repetitive element families further resolve the DNA repeat architecture of both genomes. Comparative genomic analyses identified experimentally validated small structural variants between the oil palm species and resolved the mechanism of chromosomal fusions responsible for the evolutionary descending dysploidy from 18 to 16 chromosomes.

## Introduction

Oil palm is the major global oil crop (Murphy 2009). Yields reach 5- to 10-fold of other oil crops, allowing oil palm to produce 33 and 45% of the world's vegetable and edible oils, respectively, while being cultivated on less than 5.5% of oil crop land mass (0.36% of agricultural land mass) (Vijay *et al*. 2016; Kushairi *et al*. 2018; Kushairi *et al*. 2019; Meijaard *et al*. 2020; Oil World 2020). Despite this remarkable productivity, continuous improvements through advances in genomics and molecular biology of the largely undomesticated crop are critical to counterbalance global demand with sustainable land utilization.

The interfertile oil palm species, *E. guineensis* and *E. oleifera*, are perennial, monoecious, monocotyledonous diploids (Dransfield *et al*. 2008) of paleotetraploid origin (Singh, Ong-Abdullah, *et al*. 2013). Each oil palm species has 16 chromosomes and a haploid genome size of ∼1.8 Gb, as determined by flow cytometry (Singh, Ong-Abdullah, *et al*. 2013). Date palm (*Phoenix dactylifera*) and coconut (*Cocos nucifera*) diverged from *Nypa fruiticans* ∼75.5 million years ago (Mya). Divergence of oil palm and date palm occurred at ∼62.5 Mya, followed by the divergence of oil palm and coconut, ∼45.1 Mya (Singh, Ong-Abdullah, *et al*. 2013; He *et al*. 2015).

Due to its higher yield relative to *E. oleifera* (Central and South American origin), *E. guineensis* (West African origin) is nearly exclusively cultivated for palm oil production. This difference is partly due to the yield increase resulting from the single gene heterotic effect of *SHELL* gene mutations exclusively in *E. guineensis* (Singh, Low, *et al*. 2013). While lower yielding, *E. oleifera* has several unique desirable characteristics, including higher unsaturated fatty acid (FA) content oil and important disease resistances (Sambanthamurthi *et al*. 2000; Noh *et al*. 2002; Prada *et al*. 2011; Barcelos *et al*. 2015). Other favorable *E. oleifera* traits include a shorter trunk and smaller canopy (advantageous for harvesting and planting density, respectively) (Sharma 2005; Corley and Tinker 2007). Given the interfertility between the species, introgression of favorable *E. oleifera* alleles into *E. guineensis* breeding materials is an attractive approach to improve commercial oil palm traits, albeit exceptionally slow due to a 10–12-year breeding cycle.

Clearly, yield and composition optimization require complete and contiguous reference genomes for both *Elaeis* species. A major step was achieved by our release of the first reference genome of *E. guineensis* (AVROS *pisifera*) and a comparative draft assembly of *E. oleifera* (Singh, Ong-Abdullah, *et al*. 2013). Subsequently, Ong *et al*. (2020) reported an extension of the EG5 *E. guineensis* reference genome using genetic linkage mapping efforts resulting in the assignment of 1,968 additional scaffolds, bringing the physical genome coverage to 79%. Furthermore, an independent draft genome sequence of an elite *E. guineensis dura* reference including 10,971 scaffolds with 1.701 Gb total scaffold length has been reported (Jin *et al*. 2016). More recently, Wang *et al*. (2023) described a chromosome-level assembly of an *E. guineensis dura* reference genome, resulting in a 1.70 Gb chromosome-scale assembly of 932 scaffolds, with an N50 of 111.6 Mb. Despite these important advances, continual improvements through the utilization of advanced complementary genome analysis platforms are necessary to allow construction of complete and contiguous genome assemblies for comprehensive comparative genomic analyses aimed at improving yield, disease resistance, and sustainability of this globally important oil crop. Here, utilizing long-read sequencing, proximity ligation sequencing, optical mapping, and genetic mapping, we report a substantially improved chromosome-scale reference genome for *E. guineensis* and the first chromosome-scale reference genome for *E. oleifera*, allowing analyses of structural variation between both oil palm species and comparative genomic analyses to other tropical crops.

## Methods & materials

### Genome sequencing, proximity ligation sequencing, and optical mapping

The *E. guineensis* (AVROS *pisifera* fruit form) and *E. oleifera* reference genomes were sequenced to 60× and 48× coverage of reads ≥ 10 Kb by PacBio Sequel sequencing, respectively. The specific oil palms used for genome sequencing are the same as those used for the initial *E. guineensis* reference genome assembly (P5) and *E. oleifera* draft reference genome (O7) (Singh, Ong-Abdullah, *et al*. 2013). Both species underwent proximity ligation sequencing (HiC and Chicago) by Dovetail Genomics, as well as Bionano optical mapping (McDonnell Genome Institute at Washington University in St. Louis, MO, USA).

### Genome assemblies

In preparation for the assembly, *E. guineensis* PacBio reads were subsampled down to 10 Kb or longer. The ≥ 10 Kb reads were then subsampled to 60x coverage of the expected 1.8-Gb genome size. SMRT Link, the PacBio analysis web application, was then used to run the Falcon pipeline resulting in P11a genome build. The resulting assembly was then polished using Quiver. After this, the resulting assembly was sent to Dovetail Genomics for Chicago and HiC assembly, designated as P11b and P11c builds, respectively. Bionano optical mapping was conducted using the Saphyr software to correct the assembly a final time, resulting in the P11d2 build (Supplementary Table 1). Bionano can use the physical DNA to estimate the distance between tagged sites and add a proportional amount of N's for the gaps. *E. oleifera* PacBio reads were loaded into SMRT Link and were filtered down to a set of reads that were ≥ 10 Kb. The reads were further subsampled for use in WTDBG2, resulting in 3,078,930 reads with 47× coverage. WTDBG2 was then run according to the default pipeline, which involved aligning the reads to themselves to create a consensus. The consensus was then aligned with 8 Kb Illumina linker libraries, and a new consensus called. This polishing step was carried out twice. The O12a assembly was then sent to Dovetail Genomics for Chicago (O12b build) and HiC (O12c build) assembly using HiRise. At this point, the original assembly was broken and joined according to the long-distance read information. The O12c assembly was then corrected using Bionano scaffolding, where the scaffolds were broken and joined according to the optical map, resulting In the O12d build. The O12d build was polished with Illumina linker library reads to generate O12e2 build (Supplementary Table 1). The resulting assemblies were quality control tested using Merqury (Rhie *et al*. 2020) and LTR Assembly Index (LAI) program (Ou *et al*. 2018). The LAI program uses the output of LTRharvest (Ellinghaus *et al*. 2008), LTR_FINDER_ parallel (Ou and Jiang 2019), and LTR_retriever (Ou and Jiang 2018) to estimate LTR assembly index. LTR_FINDER_ parallel and LTRharvest were run using default values. BUSCO5 (genome mode) (Seppey *et al*. 2019) analysis was carried out using the Liliopsida profiles.

The EG11 pseudochromosomes were constructed using ALLMAPS (Tang *et al*. 2015; Sanusi *et al*. 2023). Markers from P2 (Zolkafli *et al*. 2021; Sanusi *et al.* 2023), T128 (Ting, Chan, *et al*. 2023), and PUP (Ting, Ordway, *et al*. 2023) genetic maps were aligned to the P11d2 build using Exonerate (Slater and Birney 2005) and any marker that had low scores (<90% match) or discrepancies in the mapping locations was removed. The pseudochromosomes were ordered and oriented based on the three genetic map information. Scaffolds were concatenated with an additional 100 nucleotides between each consecutive scaffold.

Heterozygosity for each reference genome was calculated using 29-fold coverage of Illumina paired-end 250-bp reads and 36-fold coverage of Illumina paired-end 150- or 250-bp reads for the reference *E. guineensis pisifera* and the reference *E. oleifera* palms, respectively. Meryl 1.4.1 (Rhie *et al*. 2020) was used to count all 27-mers in the input reads as well as histograms of the counts. Finally, histograms were analyzed with GenomeScope 2.0 (Ranallo-Benavidez *et al*. 2020) with *p* = 2 and *k* = 27. *E. guineensis* reported haploid length of 1.71 Gb, unique 42.5%, kcov 7.22, error 0.0414%, and duplication 0.547. *E. oleifera* reported haploid length of 1.91 Gb, unique 39.2%, kcov 12, error 0.074%, and duplication 0.751.

### Chromosome preparation and fluorescence in situ hybridization (FISH)

Chromosome spreads were prepared from root tips of *E. guineensis* and *E. oleifera* lines maintained at the MPOB Research Station, Kluang, Johor, Malaysia. Lines were the same as those previously used for the initial *E. guineensis* reference genome and *E. oleifera* draft genome (Singh, Ong-Abdullah, *et al*. 2013). In situ hybridization was performed according to Schwarzacher and Heslop-Harrison (2000) and Zaki *et al*. (2021). In brief, a total of 40 µl probe was applied per slide, containing 50% (v/v) formamide, 20% (w/v) dextran sulfate, 2x SSC, 0.25% (w/v) sodium dodecyl sulfate, 0.25 mM ethylenediamine-tetraacetic acid, and 20 pmol and/or 25–100 ng of the oligonucleotide and retrotransposon/rDNA, respectively. Probe and chromosomal DNA were denatured together on a heated block (Thermo Fisher Scientific) at 73°C for 5 min under plastic coverslips and incubated at 37°C overnight. A series of post-hybridization washes were carried out with 2x SSC and 0.1x SSC at 42°C. DAPI (4,6-diamidino-2-phenylindole) in Citifluor AF1 (Chem Lab) antifade solution was used to counterstain the chromosomes. Previously published Gypsy (Friesen *et al*. 2001), LINE (Kubis *et al*. 2003), and Copia (Zaki *et al*. 2017) probes were used, and primer sequences for probe amplification are provided in Supplementary Table 9.

The selected oligos were designed from chromosome regions ranging from 200 to 500 Kb of *E. guineensis* genome assembly EG9 (Zaki *et al*. 2021), an unpublished and an updated version of the EG5 oil palm genome assembly (Singh, Ong-Abdullah, *et al*. 2013). Clone pTa794, containing the 5S rRNA genes and intergenic spacer from wheat (Gerlach and Dyer 1980), was used as the 5S rDNA probe labeled with digoxigenin-11-dUTP random priming (Invitrogen) and detected by FITC antidig.

Photographs were taken on a Nikon Eclipse N80*i* fluorescent microscope equipped with a DS-QiMc monochromatic camera (Nikon, Tokyo, Japan). Each metaphase was captured with three different filter sets and then overlaid and further analyzed with Adobe Photoshop CS5 (Adobe Systems, San Jose, CA, USA) or NIS-Elements BR3.1 software (Nikon) using only cropping and functions affecting the whole image equally.

### Gene prediction

Gene models were predicted using two approaches: Mikado (Venturini *et al*. 2018) and Seqping (Chan, Rosli, *et al.* 2017) pipelines. The Mikado *E. guineensis* transcript dataset was generated using 321 *E. guineensis* transcriptome libraries from various tissues in 19 BioProjects in GenBank (DRA001857, PRJDB4476, PRJEB17971, PRJEB27915, PRJEB33139, PRJEB7252, PRJNA181193, PRJNA186405, PRJNA201497, PRJNA245226, PRJNA263523, PRJNA305816, PRJNA306710, PRJNA345530, PRJNA490055, PRJNA530030, PRJNA543528, PRJNA553301, and PRJNA66349) and 8 in-house unpublished projects, while the *E. oleifera* transcript dataset was from 50 transcriptome libraries from 5 in-house unpublished projects. Each transcriptome library was mapped to the reference genome using STAR (Dobin *et al*. 2013) and individually assembled with StringTie (Pertea *et al*. 2015). The assembled transcripts were merged using Mikado, with a maximum of 10 isoforms per locus. When more isoforms were detected, the top 10 high scoring isoforms were selected. Mikado uses Portcullis (https://github.com/EI-CoreBioinformatics/portcullis) and TransDecoder (https://github.com/TransDecoder/TransDecoder) to calculate reliable splicing junctions from each alignment and, in the presence of ORF, resolves fusion proteins. The second approach, Seqping uses MAKER2 (Holt and Yandell 2011) to combine predictions from SNAP (Korf 2004), AUGUSTUS (Stanke *et al*. 2008), Fgenesh (Solovyev *et al*. 2006), and GlimmerHMM (Majoros *et al*. 2004) to *de novo* predict genes from the EG11 and EO12.1 assemblies. Seqping was run with additional supporting evidence, described as follows: (1) Mikado transcript dataset, (2) Cupcake ToFU (https://github.com/Magdoll/cDNA_Cupcake) clustered Iso-Seq data, (3) mapped transcripts from published data (Bourgis *et al*. 2011; Dussert *et al*. 2013; Shearman *et al*. 2013; Teh *et al*. 2016), and (4) liftover by UCSC liftOver (https://genome.ucsc.edu/cgi-bin/hgLiftOver) of gene model annotations [including coding sequence (CDS) information] from EG5 assembly in GenBank (gene model version 102) and from Chan, Tatarinova, *et al*. (2017).

The two sets of Mikado and Seqping gene models were combined using R, whereby only one Seqping transcript was maintained if the transcript from both Mikado and Seqping had the same transcript start and stop sites, as well as CDS coordinates. If the transcript start and stop sites were identical, but had different exon boundaries, the transcripts were retained and assigned to the same gene. In the subsequent step, long non-coding RNA (lncRNA) were identified and removed from the transcript datasets. lncRNA were identified using two approaches, namely CPC2 (Coding Potential Calculator 2) (Kang *et al*. 2017) and LncFinder (Han *et al*. 2019). Transcripts that were identified by both or either one of the two methods and did not have significant homology (e-value cutoff: 1e-5) to Genbank's RefSeq database were considered as candidate lncRNAs.

Gene ontology annotation and classification via OmicsBox were carried out using the protein sequences translated from the CDS of the transcripts. Protein sequences that did not have any gene ontology classifications were analyzed to determine whether there were any additional evidences to support the gene predictions. Sequences that had at least two independent support evidences, from BLASTP homology results, Cap Analysis of Gene Expression (CAGE) or RNA-seq data, were combined with the protein sequences with gene ontology annotations for further analysis. The combined set of transcripts was manually inspected using a multi-genome viewer, Persephone (Persephone Software; https://persephonesoft.com) to determine if transcripts from different genes were within 150 nt and 300 nt of each other and the genes were in the same direction or on opposite strands with overlapping 3′ regions, respectively. Transcripts that did not have good RNA-seq support for their exon boundaries were removed. The remaining transcripts were defined as the final selected set of transcripts and gene models for the reference genome. BUSCO5 quality assessment was carried out using Liliopsida profiles (Seppey *et al*. 2019) on all transcripts.

### Gene annotation

Gene ontology, enzyme code, EggNOG orthologs, and KEGG pathway annotations of the transcripts were determined using the following procedure. Protein sequences translated from the CDS of transcripts were searched for protein homology using BLASTP (e-value cutoff: 1e-5) against the GenBank RefSeq protein database, followed by the GenBank non-redundant (nr) protein database for gene models with no significant hit to RefSeq sequences. The BLASTP searches were limited to proteins that were listed under the embryophyte (txid3193) but not *Elaeis* (txid51952) taxonomy, and not annotated as hypothetical, predicted, uncharacterized, unnamed, unknown, low quality, or partial genes. The filtered RefSeq and nr datasets downloaded on 18-Feb-2022 consisted of 4,339,261 and 8,884,589 proteins, respectively. InterProScan (Jones *et al*. 2014) was used to search for protein functions in the following databases: CDD-3.18, Coils-2.2.1, Gene3D-4.3.0, Hamap-2020_05, MobiDBLite-2.0, PANTHER-15.0, Pfam-33.1, Phobius-1.01, PIRSF-3.10, PIRSR-2021_02, PRINTS-42.0, ProSitePatterns-2021_01, ProSiteProfiles-2021_01, SFLD-4, SignalP_EUK-4.1, SMART-7.1, SUPERFAMILY-1.75, TIGRFAM-15.0, and TMHMM-2.0c. The BLASTP and InterProScan results in XML format were imported into OmicsBox to determine the final gene annotation.

### Gene model independent support data

For transcripts to be determined as supported by CAGE sequencing data, the CAGE tag should be located from −3000 to 10 nt of the CDS start. One CAGE tag could be associated with multiple transcripts (isoforms) from one gene but not with other genes. Raw reads were trimmed using fastx_trimmer (http://hannonlab.cshl.edu/fastx_toolkit/), with PHRED quality threshold of 33 and length 50. rRNA sequences were removed from the reads using rRNAdust v 1.06 (https://fantom.gsc.riken.jp/5/suppl/rRNAdust/). Reads were aligned to the reference genome using the Burrow-Wheeler Aligner software (Li and Durbin 2009), and the remaining unaligned reads were mapped again using HISAT2 (Kim *et al*. 2019). Mapped reads were clustered to identify CAGE Transcription Start Sites (CTSSs) using FANTOM consortium scripts (Lizio *et al*. 2015). For RNA-Seq support data, the transcripts were generated using Illumina paired-end RNA sequencing data from 34 samples across different tissue types (4 leaf, 6 root, 7 mesocarp, 5 kernel, 4 whole fruit, 3 embryo, 4 flower, and 1 germinated seedling). The Illumina sequencing reads were trimmed and filtered with Trim Galore, and mapped to the reference assembly using STAR 2-pass. Expression data were generated by StringTie. BLASTP results were obtained based on a filtered Liliopsida database, where “hypothetical proteins” and “low-quality proteins” sequences were removed. The coverage and e-value thresholds were 50% and 1e-5, respectively.

### Classification of gene models

Gene models were classified based on Illumina expression data (RNA-Seq), CAGE expression data, and BLAST alignment results, as described in the *Gene Model Independent Support Data* section. Six classes of gene models were defined based on the following support data: (1) Class N1—CAGE, RNA-Seq and BLAST result; (2) Class N2—CAGE and either RNA-Seq or Blast results; (3) Class N3—RNA-Seq and BLAST results; (4) Class N4—CAGE or RNA-Seq data only; (5) Class N5—BLAST results only; and (6) Class N6—Not supported by experimental evidence.

### Representative genes

The representative isoform for each gene was selected based on the following criteria: (1) Highest class (N1-N6); (2) Best e-value for BLAST; (3) Expressed in the highest number of tissues; (4) Highest transcripts per million value; (5) Longest CDS; and (6) First model predicted by gene prediction pipeline. First, the isoforms with the best gene model class were identified. In cases where there are multiple isoforms within the best class, they were ranked by the BLAST E-value, breadth of tissues expressed, level of expression, and length of the coding region (criteria 2–5). If this ranking still resulted in more than one isoform, the first isoform of the gene was selected.

### Methylation analysis

Methylation data of oil palm ortets were obtained from Ong-Abdullah *et al*. (2015). Quality control checks of the reads were performed by FastQC v0.11.3 software (Babraham Bioinformatics). To remove adapter contaminants and low-quality reads, all reads were trimmed using Trimmomatic v0.36 (Bolger *et al*. 2014). Trimmed reads were mapped to EG11 using Bismark v0.16.3 (Krueger and Andrews 2011) and Bowtie2 v2.2.5 (Langmead and Salzberg 2012). Alignments to complementary strands (CTOT and CTOB) were ignored. Using SAMtools v1.9 (Li *et al*. 2009), read deduplication was performed by Bismark before the identification of the methylation state of all cytosines in a CG, CHG, and CHH context using the bismark_methylation_extractor modules. Positions within +/− 500 nt of the transcription start site and transcription start exon were extracted from Bismark output. Average methylation was calculated for each context and averaged in 5 nt windows.

### Genomic repeat analyses

Four genomes, namely EG11, EO12.1, *P. dactylifera* [BioProject Accession No.: PRJNA692501 (Al-Mssallem *et al*. 2013)] and *Cocos nucifera* [coconut (Xiao *et al*. 2017; Yang *et al*. 2021)] were analyzed for their repetitive elements using RepeatModeler2 [version 2.0.1 (Flynn *et al*. 2020)]. The pipeline uses two discovery algorithms, RepeatScout [version 1.0.6 (Price *et al*. 2005)] and RECON [version 1.0.8 (Bao and Eddy 2002)], followed by consensus building and classification of transposable elements (Tes). First, genomic sequences of the four genomes were combined, and a database was built. Next, RepeatModeler2 pipeline was deployed using the genomeSampleSizeMax parameter of 6,700 Mb sample size that encompasses the total size of the four genomes. After all the rounds of discovery, refinement, and merging, the final library was classified via RepeatClassifier (version 2.0.1). In the final step, the four genomes were masked and annotated via RepeatMasker software (version 4.1.1). This package used RMBlast (version 2.11.0), TRF (version 4.0.9), Python (version 3.8.8), and Perl (5.26.2).

Karyoplots were created using an R package KaryoploteR (Gel and Serra 2017) with a window size of 1 Mb. The density of genes and repeats was calculated by KaryoploteR with the same window size and extracted into tables. Then, Spearman correlation was calculated for these 1 Mb segments. The correlation was plotted using the R package corrplot [https://cran.r-project.org/web/packages/corrplot/vignettes/corrplot-intro.html (Wei and Simko 2021)]. Negative or positive correlation in the 1 Mb region indicates that the genes in the region were negatively or positively selected in comparison to the repeat element, respectively.

### Marker density plots on chromosomes

MapChart 2.32 program (Voorrips 2002) was used to draw the individual chromosomes based on the physical position of markers. Chromosomes were presented as vertical bars, while each horizontal line represented a marker.

### Comparative genomic analyses

Comparative genomic analyses were performed using Synvisio (https://synvisio.github.io/) to compare EG11 and EO12.1 to published genomes of date palm (Al-Mssallem *et al*. 2013), coconut (Xiao *et al*. 2017; Yang *et al*. 2021), and banana (D'Hont *et al*. 2012). Ortholog analysis of *E. guineensis, E. oleifera*, and coconut gene models was carried out using EggNOG (Hernández-Plaza *et al.* 2023) in OmicsBox. The date palm orthologs in each of the orthologous groups identified were determined from the EggNOG database.

To identify potential structural variations (SVs) between oil palm species, PacBio subreads generated for *E. oleifera* were aligned to both EG11 and EO12.1 using nglmr (https://github.com/philres/ngmlr), which aligns reads in the presence of structural variations. The resulting alignments to EG11 were used to call structural variants using Sniffles (https://github.com/fritzsedlazeck/Sniffles). The putative structural variants for deletions, duplications, and translocations were examined in Integrative Genomics Viewer (IGV).

To identify additional large SVs, SYRI (Goel *et al*. 2019) was used for pairwise assembly comparisons of EG11 and EO12.1. EO12.1 was aligned to EG11 using minimap 2 v.2.26 (Li 2021) with settings “—eqx -t 48 -x asm5.” After alignment, SYRI v1.6.3 was used with default parameters to detect putative inversions, duplications, and translocations/transpositions. All predicted SVs mapping to a gap in one or both assemblies were filtered out. Visualizations of predicted SVs were built using plotsr v.1.1.0 (Goel and Schneeberger 2022). For each SV type, the size distribution of predicted SVs was plotted and used to identify natural breaks in the distributions of predicted SV sizes. Accordingly, inversions ≥ 5 MB and duplications or translocations ≥ 50 KB were selected as predicted large SVs.

### PCR-based small structural variant validation

Sniffles-based alignments that had strong evidence were chosen to be examined using PCR. PCR was performed using 10 ng reference genomic DNA, 0.6 µM forward and reverse primers, and 1x Quanta Toughmix. The products were run on 1% agarose gel to confirm amplification and size. PCR with expected-sized products and correct genome specificity were used as evidence to support SV.

EG11 was considered the reference genome with deletions observed in EO12.1. Two sets of primer pairs were designed for each of the 18 potential deletions. The first set was designed with the forward primer (D1F) in sequence present in both EG11 and EO12.1 sequence, 5′ to the deletion region (Supplementary Fig. 7a). The reverse primer (D1R) was designed for sequence only present in EG11 and not in EO12.1. The second set was designed with the forward primer (D2F) in sequence only found in EG11 and absent in EO12.1. The reverse primer (D2R) was designed for sequence found in both EG11 and EO12.1, 3′ to the deletion region. Each set would only amplify from *E. guineensis* genomic DNA when a deletion is present in the corresponding region of *E. oleifera* genomic DNA. The forward primer in the first set and the reverse primer in the second set span the deletion region and would only amplify *E. oleifera* genomic DNA when a deletion is present.

EG11 was considered the reference genome with duplications observed in EO12.1. Primer pairs were designed to amplify within the duplication regions. The forward primer (U1F) was designed from the 3′ end of the duplicated region, and the reverse primer (U1R) was designed from the 5′ end of the duplication (Supplementary Fig. 7b). These primers would only amplify the *E. oleifera* genomic DNA, where tandem duplications are present.

EO12.1 was considered the reference genome with translocations present in EG11. Two sets of primers were designed for each translocation region. The first set was designed with the forward primer (T1F) 5′ to the translocation. The reverse primer (T1R) was designed 3′ to the translocation junction. The second set was designed on the corresponding chromosome translocation with the forward primer (T2F) 5′ to the translocation and the reverse primer (T2R) 3′ to the translocation junction (Supplementary Fig. 7c). The separate sets would amplify *E. guineensis* genomic DNA where translocation had occurred, and not in *E. oleifera*.

## Results and discussion

### Sequencing and assembly of *E. guineensis* and *E. oleifera* reference genomes

**Fig. 1.**
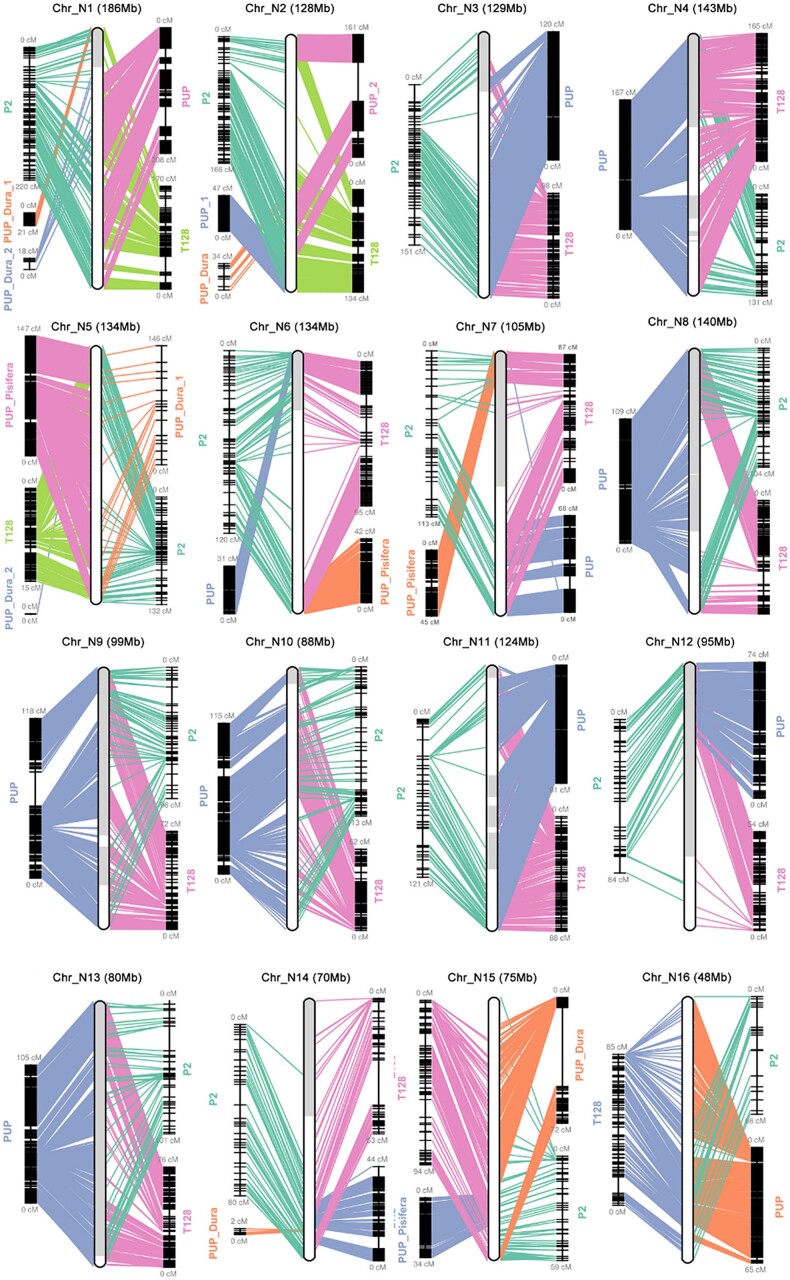
Genetic map-guided *E. guineensis* reference genome (EG11) pseudochromosome assembly. For each of the 16 pseudochromosomes, flanking T128, P2, and PUP genetic map positions are shown with lines connecting to the central physical positions on the assembled pseudochromosomes. Subgroups in genetic maps are labeled as _1 and _2.

The improved *E. guineensis pisifera* reference genome (hereafter referred to as EG11) was assembled using 60X PacBio sequencing reads (≥ 10 kb). A starting assembly was constructed and polished using the default SMRTLink configuration. Continuity and accuracy of the initial assembly were then improved by Dovetail HiRise using Chicago (P11b build) and HiC (P11c build) proximity ligation sequencing (Methods). Bionano optical mapping (Methods) was utilized to further improve the assembly (P11d2 build). Pseudochromosomes were assembled using a genetic map-guided approach (Fig. 1, Supplementary Fig. 1-2, Methods), resulting in 16 pseudochromosomes with gap content of 5.63% and 1.78 Gb of total sequence (∼99% of the flow cytometry determined *E. guineensis* genome size) (Singh, Ong-Abdullah, *et al*. 2013) and N50 length of 128.3 Mb [range, 93.2 Kb–186.2 Mb (Table 1)]. These improvements represent a 1000-fold reduction in scaffold number, a 100-fold increase in scaffold N50, and a 4-fold decrease in gap content relative to the EG5 assembly [Supplementary Table 1 (Singh, Ong-Abdullah, *et al*. 2013)]. Relative to the Wang *et al. dura* genome assembly (Wang *et al*. 2023), EG11 incorporates ∼10% more of the predicted genome size into the assembly (1.87 Gb vs 1.70 Gb), with ∼24-fold reduction in scaffold number (39 vs 932 scaffolds), ∼15.4% higher pseudochromosome N50 (128.3 Gb vs 111.6 Gb), and includes 13,000 more predicted evidence supported genes (46,697 vs 33,447) (Supplementary Table 1). These differences are likely provided by the integration of 60X PacBio Sequel sequencing with proximity ligation sequencing and optical mapping, relative to ∼2.5-fold more PacBio sequences alone.

**Table 1.** EG11 and EO12.1 genome assembly statistics.

		EG11	EO12.1
**General Statistics**			
	No. of Scaffolds	39	26
	Total bases	1,867,267,987	2,042,916,827
	Guanine-Cytosine (GC) content	38.54%	39.33%
	Gap content	7.95%	14.37%
	Maximum length	186,201,113	226,035,659
	Minimum length	93,154	89,593
	Mean length	47,878,666	78,573,724
	N50 length	128,314,321	141,087,874
	N90 length	69,731,926	79,040,982
**16 Pseudochromosomes**			
	Total no. bases	1,777,716,855	2,022,763,378
	% Gap	5.63%	13.76%
**Quality Assessment [BUSCO5 Liliopsida Profile (Genome Mode)]**			
	Complete BUSCOs	2,965 (91.6%)	3,022 (93.3%)
	Complete and single copy BUSCOs	2,582 (79.8%)	2,629 (81.2%)
	Complete and duplicated BUSCOs	383 (11.8%)	393 (12.1%)
	Fragmented BUSCOs	180 (5.6%)	161 (5.0%)
	Missing BUSCOs	91 (2.8%)	53 (1.7%)
	Total BUSCO groups searched	3236	3236
**Merqury**			
	QV Score	32.1	33.4
	k-mer completeness	91.7%	97.2%
	False duplications	7.1%	2.4%
**LTR Assembly Index**			
	LTR Index	15.33	18.93

The *E. oleifera* reference genome assembly (hereafter referred to as EO12.1) followed a similar assembly strategy (Methods) beginning with 48X coverage PacBio sequencing reads (≥ 10 Kb) assembled into a polished initial assembly (O12a build), followed by HiRise Chicago (O12b build) and HiC (O12c build) proximity ligation sequencing and optical mapping, and additional polishing using shotgun sequencing reads [O12e2 build (Supplementary Table 1)]. The 16 pseudochromosome EO12.1 assembly consists of 26 scaffolds with N50 length 141.1 Mb (range, 89.6 Kb–226.0 Mb), gap content of 14.37%, and 2.04 Gb of total sequence (Table 1, Supplementary Table 1). Relative to the initial draft *E. oleifera* genome (Singh, Ong-Abdullah, *et al*. 2013), EO12.1 represents a 1000-fold reduction in scaffold number, increases scaffolded bases by 45.7% (Supplementary Table 1) and is the first chromosome-scale *E. oleifera* reference genome reported to date, highly similar in quality to the EG11 *E. guineensis* reference. Consistent with the slightly larger genome size of *E. oleifera* relative to *E. guineensis* based on reported flow cytometry measurements (Camillo *et al*. 2014), total bases included in the EO12.1 assembly are 9.4% higher than in the EG11 assembly. Although gap content is improved for both EG11 and EO12.1, some gaps remain in each pseudochromosome, and therefore, none meet the strict definition of telomere-to-telomere assemblies. Heterozygosity was estimated for each reference palm (Methods). As expected, considering the ancestry of the 2 reference palms, *E. guineensis* is predicted to be approximately 3-fold more heterozygous than *E. oleifera* (0.745 and 0.261% heterozygosity, respectively).

For the EG11 and EO12.1 assemblies, 46,697 and 38,658 gene models (105,560 and 80,520 transcripts) were predicted, respectively, representing 79.3% complete and 8.7% fragmented BUSCO genes (Seppey *et al*. 2019) for *E. guineensis* and 88.8% complete and 4.7% fragmented BUSCO genes for *E. oleifera* (Supplementary Table 2). *E. guineensis* and *E. oleifera* transcript datasets had 12.0 and 6.5% missing BUSCOs, respectively, probably due to prediction inaccuracies. Nevertheless, losses (14.6% of missing BUSCOs) detected in the assemblies of both oil palm species could have been due to selection over evolutionary time.

Assembly accuracy and completeness were assessed by Merqury [Supplementary Fig. 3, Methods (Rhie *et al*. 2020)], which reported assembly consensus quality values (QV) of 32.1 and 33.4 (>99.9% accuracy), k-mer completeness (fraction of reliable K-mers in the read set that are also found in the assembly) of 91.7 and 97.2%, and false duplications of 7.1 and 2.4% for EG11 and EO12.1, respectively (Table 1). The genome assembly quality was also assessed using LAI, where *E. guineensis* and *E. oleifera* genome assemblies had LAI scores of 15.33 and 18.93, respectively (Table 1). These LAI values fall within the classification of reference quality assembly, as classified by Ou *et al*. (2018). These scores are higher than that of the Arabidopsis reference genome, with an LAI score of 14.9, and slightly lower than that of the rice reference genome (LAI score = 21.1; “gold standard”: LAI ≥ 20).

### Refinement of *E. guineensis* and *E. oleifera* reference assemblies

**Fig. 2.**
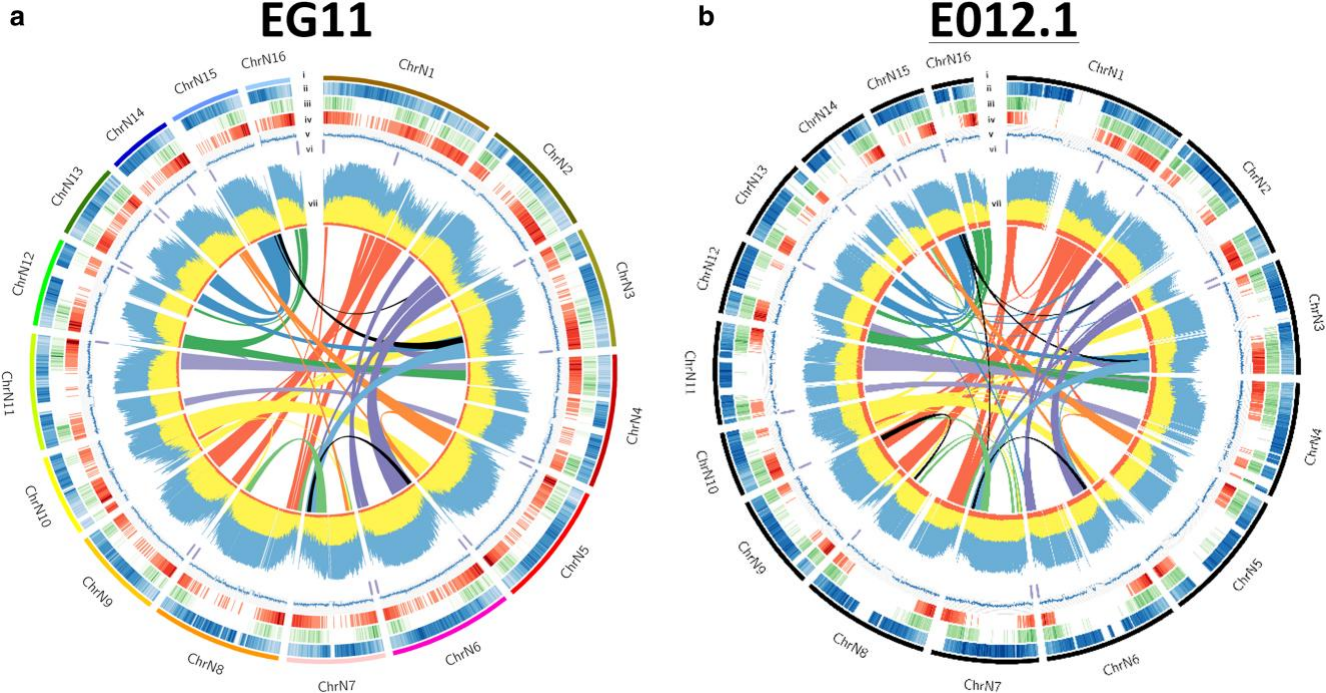
*E. guineensis* (EG11) and *E. oleifera* (EO12.1) genome features. Circos plots of a) EG11 and b) EO12.1 genome assemblies. Tracks include i) the 16 oil palm pseudochromosomes, ii) Type I retrotransposons, iii) Type II DNA repeats, iv) gene density, v) G + C content, vi) telomeric repeats, and vii) DNA methylation density. DNA methylation density is plotted for CG (blue), CHG (yellow), and CHH (red) on the same scale. Intra-genomic synteny plots are drawn in the center of each circus plot. Colored lines represent regions of gene order homoeologous relationships (segmental duplications) between different pseudochromosomes of the same species.

**Fig. 3.**
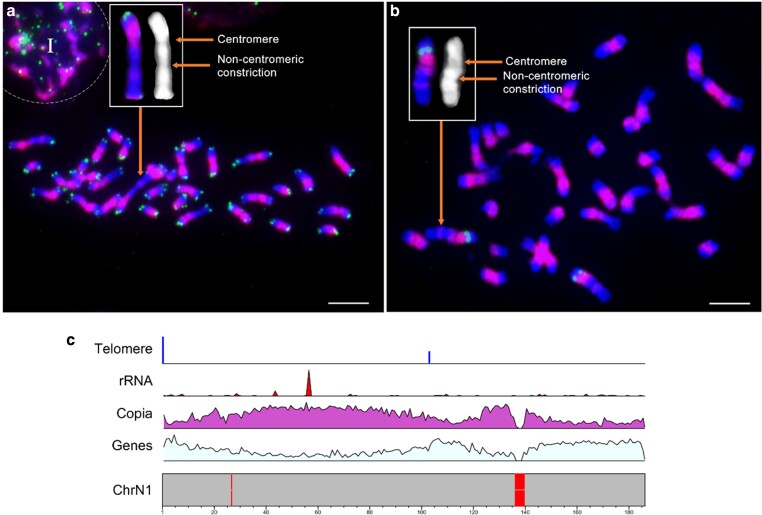
Reassignment of pseudochromosomes 1 and 2 of EG11. Pseudochromosome 2 of the previous EG5 assembly (Singh, Ong-Abdullah, *et al*. 2013) was reassigned as Chromosome N1, as it is the longest chromosome in oil palm. In situ hybridization of mitotic chromosomes hybridized with a) Copia (magenta) and telomere (green) probes and b) Copia (magenta) and 5S rDNA (green) probes (adapted from the study by Zaki *et al*. 2021). The 32 chromosomes were counterstained with DAPI (blue). Boxed chromosome shows the two constriction sites in the largest chromosome of the oil palm. The non-centromeric constriction site is located opposite to the arm carrying 5S rDNA. I alphabet in panel a) indicates the nonspecific background. Bars: 5 µm c) Distribution of telomeric, rRNA, Copia, and gene sequences in pseudochromosome N1. The red bars indicate gap regions that do not contain any sequence reads.

Oil palm chromosomes have proven challenging to distinguish cytogenetically, but fall into four groups, comprising (i) the largest chromosome which hybridizes to 5S rDNA, (ii) 8 medium-sized chromosomes, (iii) 6 small chromosomes, and (iv) a small acrocentric chromosome including 18S-25S rDNA (Madon *et al*. 1995; Castilho *et al*. 2000). Previous attempts to identify specific chromosomes by fluorescence in situ hybridization (FISH) using RFLP clones from early genetic maps were unsuccessful in unambiguously assigning chromosome identification numbers (Singh, Ong-Abdullah, *et al*. 2013). Therefore, pseudochromosomes were numbered according to the base pair size of sequence scaffolds. The largest EG5 scaffold (68,432,966 bp) was assigned as chromosome 1, with the next largest (65,556,141 bp) assigned as chromosome 2 (Singh, Ong-Abdullah, *et al*. 2013). However, in the improved EG11 assembly, pseudochromosome sizing suggested that the previously assigned chromosome 1 is instead chromosome 2, and *vice versa*. Furthermore, telomeric repeats embedded within a putative centromeric region, which were previously localized to pseudochromosomes 2 and 14 of EG5 (Singh, Ong-Abdullah, *et al*. 2013), are localized to the EG11 assembly pseudochromosomes N1 and N14 (Fig. 2a). Cytogenetically, it appears that the centromere corresponds to a Copia dense region on the same chromosome with distinct 5S rDNA staining (Fig. 3b). Collectively, these findings support the reciprocal reassignment of pseudochromosomes N1 and N2 in EG11 and EO12.1. Note that the inclusion of an “N” prefix in the pseudochromosome numbers of EG11 and EO12.1 is intended to avoid confusion given this reciprocal reassignment relative to previously published genomes. For example, pseudochromosome 1 of the EG5 *E. guineensis* reference (Singh, Ong-Abdullah, *et al*. 2013) is designated pseudochromosome N2 in EG11. Likewise, EG5 pseudochromosome 2 is designated pseudochromosome N1 in EG11 (Supplementary Table 3).

Comparison of EG11 to genetic maps (Fig. 1, Supplementary Fig. 1) identified and corrected a previous misassembly in pseudochromosome N5 (Supplementary Fig. 4). The HiRise assembly resulted in the merger of distal ends of pseudochromosome N5. The breakpoint of the incorrect merger was identified via marker positions of the T128 (Ting, Chan, *et al.* 2023), P2 (Zolkafli *et al*. 2021; Sanusi *et al.* 2023), and PUP (Ting, Ordway, *et al*. 2023) genetic maps, contigs of the different assemblies and identification of the telomeric sequences. The correction involved the removal of 10.7 Mb from the distal end of pseudochromosome N5 and the joining of the sequence to the opposite distal end of the pseudochromosome, as supported by the genetic map marker positions. Pseudochromosome N7 and one of the distal ends of pseudochromosome N13 that were merged into a single scaffold in the O12e2 build were also corrected using syntenic positions of the *E. guineensis* and *E. oleifera* chromosomes and marker positions of the T128, P2, and PUP genetic maps.

Following HiRise and optical mapping, the *E. oleifera* assembly was compared to the EG11 assembly. Given that the two species are interfertile, highly conserved synteny between the two genome assemblies is expected. A total of 33 scaffold-scale differences were detected, involving relatively minor scaffold inversions of the *E. oleifera* assembly relative to that of *E. guineensis.* Evidence supporting or contradicting each potential correction was investigated within the proximity ligation data alone, the optical mapping data alone and assembled reads of bacterial artificial chromosome (BAC) pools. Of the 33 candidate corrections, 17 had direct data supporting the corrected version. No direct evidence contradicting a potential correction was found. Therefore, all 33 corrections were made, thus completing the EO12.1 assembly (Supplementary Table 4).

**Fig. 4.**
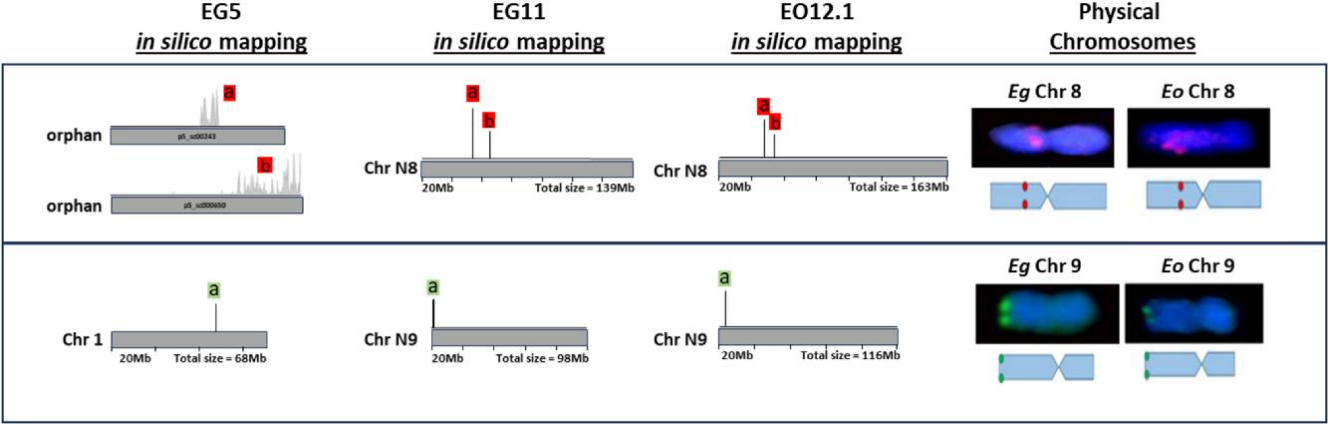
Cytogenetic mapping validates improvements to EG11, relative to P5. Cytogenetic analyses of chromosomes 8 and 9 resolved the previous misassembly in EG5 or incorporated genome regions that were not assembled into pseudochromosomes (orphans) in EG5 but were incorporated into pseudochromosomes N8 or N9 in EG11. Gray horizontal bars represent pseudochromosome assemblies or orphan scaffolds of EG5 (left) or pseudochromosome assemblies of EG11 (middle) or EO12.1 (right). *In silico* mapping of probe sequences are represented by vertical lines. Colored boxes indicate the fluorescent labeling of the associated probes. Images of cytogenetically stained physical chromosomes are shown with a diagrammatic representation of the staining pattern below the image (physical chromosomes).

Two chromosomes for which EG11 pseudochromosome assemblies were different relative to the previous EG5 pseudochromosomes were further analyzed by cytogenetic staining to provide validation support for the EG11 and EO12.1 corrections of EG5 misassemblies or exclusion of orphan scaffolds (Methods). *In silico* mapping of EgOligoFISH (Zaki *et al*. 2021) probe sequences to EG5, EG11, and EO12.1 identified two independent EG5 orphan scaffolds that were incorporated into an internal region of pseudochromosome N8 of EG11 and EO12.1, as well as probe sets *in silico* mapping to EG5 pseudochromosome 1 (analogous to EG11 and EO12.1 pseudochromosome N2) that instead mapped to pseudochromosome N9 of EG11 and EO12.1 (Fig. 4). Although the cytogenetics results were not utilized to guide the assemblies, in each of these cases, the staining patterns of the relevant metaphase chromosomes were consistent with the EG11 and EO12.1 pseudochromosome assemblies, providing physical evidence for appropriate pseudochromosome assembly in the improved reference genomes.

### Intra-genus comparative genomics: *E. guineensis* vs *E. oleifera*

Circos plots of the EG11 and EO12.1 assemblies demonstrate that the two interfertile species have very similar genome configurations in terms of gene density, repeats, and DNA methylation (Fig. 2, Methods). As previously reported (Singh, Ong-Abdullah, *et al*. 2013), a comparison of each species’ chromosomes to themselves demonstrates consistent large segmental duplications in both species (Fig. 2, Methods). For example, in both species, chromosomes N1, N8, and N9 share extensive regions of synteny and conserved gene order. Likewise, chromosomes N2 and N6 share syntenic regions. In fact, every chromosome shares substantial synteny with at least one other chromosome of the genome. These findings indicate that the segmental duplications occurred prior to the divergence of the African and South American oil palm species ∼51 Mya (Singh, Ong-Abdullah, *et al*. 2013) and that oil palm species are ancient palaeotetraploids. The high conservation between the two genomes explains the interfertility between the African and South American species, giving rise to fertile hybrids (Hardon and Tan 1969). As hypothesized previously (Singh, Ong-Abdullah, *et al*. 2013), this conservation remains consistent with the vicariant hypothesis for phylogeographical divergence, in which geographically isolated species are not under selective pressure to evolve reproductive isolation (Riggins and Seigler 2012).

### Inter-genus comparative genomics: oil palm vs date palm and coconut

**Fig. 5.**
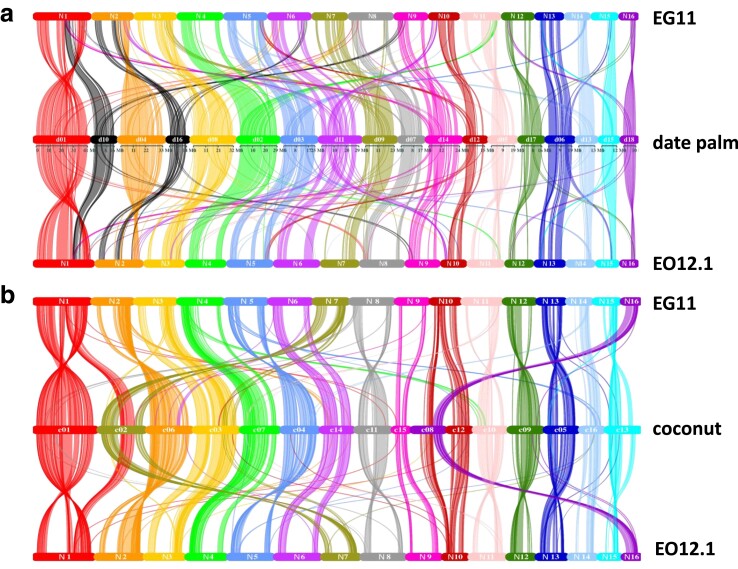
Comparative genomics of oil palm species, date palm, and coconut. a) *E. guineensis*, *E. oleifera,* and *Phoenix dactylifera* (date palm) gene order synteny. EG11 and EO12.1 pseudochromosomes are depicted in order from chromosomes 1 to 16. The 18 date palm reference chromosome assemblies are depicted in the order that is most homologous to oil palm. Lines represent regions of gene order homoeologous relationships. b) *E. guineensis*, *E. oleifera* and *Cocos nucifera* (coconut) gene order synteny. The 16 chromosome assemblies of the coconut reference genome are depicted in the order that is most homologous to oil palm.

We previously reported the identification of interstitial telomeric repeat arrays embedded within putative centromeric regions of pseudochromosomes N1 (previously annotated as chromosome 2) and N14 (Singh, Ong-Abdullah, *et al*. 2013). We hypothesized that these chromosomes arose as Robertsonian fusions of telocentric chromosome ends which are, by definition, end-to-end-type chromosomal fusions. Reported size estimates of the date palm (*P. dactylifera*) genome range from ∼671 Mb (Al-Mssallem *et al*. 2013) to ∼880 Mb (Hazzouri *et al*. 2019), 50–60% smaller than the oil palm genome. Comparative genomic alignments (Methods) of the 16 chromosomes of *E. guineensis* and *E. oleifera* to the 18 chromosomes of date palm (*P. dactylifera*) demonstrate a mostly pairwise syntenic relationship between oil palm and date palm chromosomes (Fig. 5a). For example, pseudochromosome N3 of EG11 and EO12.1 share synteny with the entirety of date palm pseudochromosome 8, while oil palm pseudochromosome N4 shares synteny with date palm chromosome 2. However, consistent with a previous comparative genomic analysis utilizing an earlier *E. guineensis* genome assembly (Ong *et al*. 2020), EG11 and EO12.1 pseudochromosome N1 [named chromosome 2 in Ong *et al*. (2020)] are syntenic with date palm chromosomes 1 and 10. Likewise, EG11 and EO12.1 pseudochromosome N2 are syntenic with date palm chromosomes 4 and 16. These results confirm the chromosomal fusion events resulting in descending dysploidy in the evolutionary transition from 18 (date palm) to 16 (oil palm and coconut) chromosomes.

Chromosomal fusions underlying descending dysploidy can occur either as end-to-end chromosomal fusions (EEF) or nested chromosomal fusions (NCF) [reviewed by Lysak (2022)]. Meiotic double-strand breaks (DSBs) or compromised capping in (sub)telomeric or telomeric regions of nonhomologous chromosomes may, if in proximity to each other, be repaired by nonhomologous end joining or nonallelic homologous recombination, resulting in EEF. In eukaryotes with monocentric chromosomes, one of the two centromeres resulting from EEF is eliminated or epigenetically inactivated. In contrast, NCF involves the insertion of one chromosome between the arms of a recipient chromosome, with the centromere of the inserted chromosome becoming the functional centromere of the fusion chromosome and the centromere of the recipient chromosome being disrupted. NCF requires DSBs in the (peri)centromeric region of the recipient chromosome and DSBs (or compromised telomere capping) at both (sub)telomeric regions of the inserted chromosome, followed by recombination between (peri)centromeric ends of the recipient chromosome and (sub)telomeric ends of the inserted chromosome. Observed NCFs in plant evolution are rare due to infrequency or instability of (peri)centromere-(sub)telomere recombination and/or subsequent chromosomal rearrangements that mask their identification (Lysak 2022). Potentially due to the comparably advanced state of genomics studies of cereal grasses compared to other plants, NCF events have primarily been documented in grasses (family Poaceae) [reviewed by Lysak (2022)].

For oil palm pseudochromosome N1, the comparative genomics alignments of oil palm to date palm demonstrates that date palm chromosome 10-syntenic sequence is inserted within the oil palm chromosomal regions syntenic to date palm chromosome 1, suggesting an NCF event (Fig. 5a, Supplementary Fig. 5a). BLAST alignments of date palm chromosomes 1 and 10, telomeric repeats, 5S rDNA, and pericentromeric repeats to EG11 pseudochromosome N1 reveal that the interstitial telomeric repeat arrays embedded in EG11 pseudochromosome N1 (Fig. 2a) map to a terminal end of the N1 sequence syntenic with date palm chromosome 10 and are likely a remnant of the date palm chromosome 10 telomere that has not been completely excised from oil palm chromosome 1 (Supplementary Fig. 5a). While the synteny pattern appears consistent with an NCF event, the mechanism of NCF dictates that the breakpoint of the recipient chromosome occurs in the centromeric region (functionally inactivating the recipient chromosome centromere) and that recombination with the inserted chromosome results in the inserted chromosome's centromere becoming the functional centromere of the fused chromosome [reviewed by Lysak (2022)]. However, the distribution of Copia elements within chromosome 1 (Fig. 3) suggests that the date palm chromosome 1 syntenic region carries the functional centromere. Given these characteristics, we propose that oil palm chromosome 1 arose from an EEF event between date palm chromosomes 1 and 10, followed by an independent intrachromosomal rearrangement that relocated a region of the chromosome syntenic with date palm chromosome 1 to the opposite side of the region syntenic with date palm chromosome 10. It is currently undetermined whether the non-centromeric Copia staining constriction visible on metaphase spreads of chromosome 1 is the remnant of the inactivated centromere within the date palm chromosome 10-syntenic region. Alignments of pseudochromosome N2 with date palm chromosomes show that the date palm chromosome 16-syntenic region is largely localized to one end of oil palm pseudochromosome N2 (Fig. 5a, Supplementary Fig. 5b), also consistent with an EEF event. Relatively small overlapping peaks of date palm chromosomes 4 and 16 synteny (Supplementary Fig. 5b) may be due to intrachromosomal rearrangements occurring after the EEF event.

Comparisons of syntenic gene order between oil palm and coconut [*Cocos nucifera* (Fig. 5b)] indicate a near one-to-one ancestral relationship between chromosomes. The coconut genome (2.4 Gb) is 34% larger than that of the oil palm, primarily due to increased repeat sequence content (73%) as a result of massive transposable element expansion within the last million years (Yang *et al*. 2021). Some rearrangements are apparent. For example, consistent with the findings of Yang *et al*. (2021), coconut chromosome 2 shares gene order synteny with oil palm chromosomes N1 and N7, and coconut chromosome 8 shares synteny with oil palm pseudochromosomes N10 and N16. These represent recombination events involving large blocks of synteny rather than entire chromosome fusion events. As expected, the gene order conservation between oil palm and banana (*Musa acuminata*, family *Musaceae*) is minimal (Supplementary Fig. 6).

### Identification of structural variants between *Elaeis* species

Although oil palm species genomes are highly structurally conserved, differences between the genomes exist. EG11 was considered the reference genome (predicted deletions or duplications refer to deletions or duplications within the *E. oleifera* genome relative to *E. guineensis*). PacBio subreads generated for EO12.1 were aligned to both EO12.1 and EG11 and used to identify candidate SVs (Methods). Candidates for deletions, duplications, and translocations with strong evidence based on a manual review of IGV alignments were selected for the design of validating PCR assays. To minimize false negative PCR reactions due to interspecies SNPs or other minor variants, PCR primers were designed so that targeted sequences shared by both species were invariant between the two species. Two sets of primer pairs were successfully designed for 18 candidate deletions (Methods), and all candidate deletions were validated by PCR. For duplications, PCR primer pairs were designed to amplify within seven candidate duplicated regions (Methods), of which four were validated by PCR. For candidate translocations, EO12.1 was considered the reference with translocations present in EG11. Two sets of primers were designed for each translocation region (Methods). 18 of 32 candidate translocations were validated by PCR assays. Representative PCR validation results are shown in Supplementary Fig. 7. Approximate chromosomal coordinates of validated small SVs, along with gene annotations, are provided in Supplementary Table 5. Structural variants may contribute to the oil yield, oil properties, and disease resistance differences between the two species. For example, one translocation variant involves a region of *E. oleifera* chromosome 7, including the 3′ end of *PTi6* gene, that is translocated to chromosome 11 in *E. guineensis* resulting in the deletion of the 3′ end of *PTi6* in *E. guineensis*. PTi6 is an ethylene-response transcription factor (ERF) that promotes bacterial and fungal disease resistance in tomatoes and grapes (Gu *et al*. 2002; Su *et al*. 2019; Wang *et al*. 2021). Future studies will be required to determine whether this variant is functionally involved in disease resistance differences between the oil palm species.

Additionally, a pairwise whole genome assembly comparison was conducted to predict large putative interspecific genomic inversions, duplications, transpositions, and translocations that may have been missed by the PacBio read alignment approach (Methods). Comparing EG11 and EO12.1, 8 inversions (>5 MB) were predicted, one each on pseudochromosomes N1, N2, N3, N4, N6, N11, N12, and N15 (Supplementary Fig. 8). Of these, 7 involve regions of high repeat content and low gene density, and therefore, the possibility that these represent inverted misassemblies of scaffolds within highly repetitive regions of the genomes cannot be ruled out. One predicted inversion involves a telomere-proximal gene-dense region of pseudochromosome 4. Five duplication events (> 50 Kb) were predicted (Supplementary Fig. 8), of which 4 are predicted duplications in EG11 relative to EO12.1 (pseudochromosomes N4, N10, N13, and N15), and 1 is a predicted duplication in EO12.1 relative to EG11 (pseudochromosome N7). Finally, three inverted translocation events were predicted (Supplementary Fig. 9) involving EG11 pseudochromosome N3 to EO12.1 pseudochromosome N10, EG11 pseudochromosome N10 to EO12.1 pseudochromosome N3 (analogous to PCR-validated event_10 in Supplementary Table 5) and EG11 pseudochromosome N6 to EO12.1 pseudochromosome N8. Genome assembly coordinates of each of the 16 predicted SV events are provided in Supplementary Table 6. Future pan-genome analyses including multiple reference palms for each species will be required to validate these SV predictions and provide higher resolution of species-specific variation across the genomes.

### Gene prediction and annotation

Gene predictions were based, in part, on whole transcriptome sequencing data from 19 BioProjects and 13 in-house unpublished projects. Trimmed and filtered reads were mapped to the reference genomes to identify regions containing expressed genes, while CAGE data were clustered to identify base-pair-accurate transcription start sites [CTSSs (Methods)]. The *E. guineensis* and *E. oleifera* Mikado (Venturini *et al*. 2018) transcript datasets with 216,830 and 101,472 transcripts, respectively, were constructed by integrating mapped transcripts from 321 *E. guineensis* and 50 *E. oleifera* RNA-Seq libraries. The Seqping (Chan, Rosli, *et al.* 2017) gene prediction pipeline, which generates and uses species-specific hidden Markov models was used for *de novo* gene prediction. Integration of both datasets resulted in the identification of 293,746 (234,523 gene models) and 250,045 (210,318 gene models) *E. guineensis* and *E. oleifera* transcripts, respectively.

Protein sequences translated from the CDS of transcripts were searched for homology using BLASTP to the GenBank RefSeq protein database, followed by GenBank nr protein database for sequences with no association to RefSeq sequences (Methods). InterProScan was used to predict protein functions in various databases (Methods). BLASTP and InterProScan results were imported into OmicsBox (Götz *et al*. 2008) for various annotations (Methods). Protein sequences with gene ontology (GO) annotation, combined with protein sequences that did not have GO annotation but had at least two independent sources of support evidence, from homology search via BLASTP, CAGE, or RNA-seq data, were used to generate a selected transcript dataset for further analysis. In total, 105,560 transcripts from 46,697 gene models, and 80,520 transcripts from 38,658 gene models were selected for *E. guineensis* and *E. oleifera*, respectively. A comprehensive gene model listing, with annotation, is provided in Data S1.

**Fig. 6.**
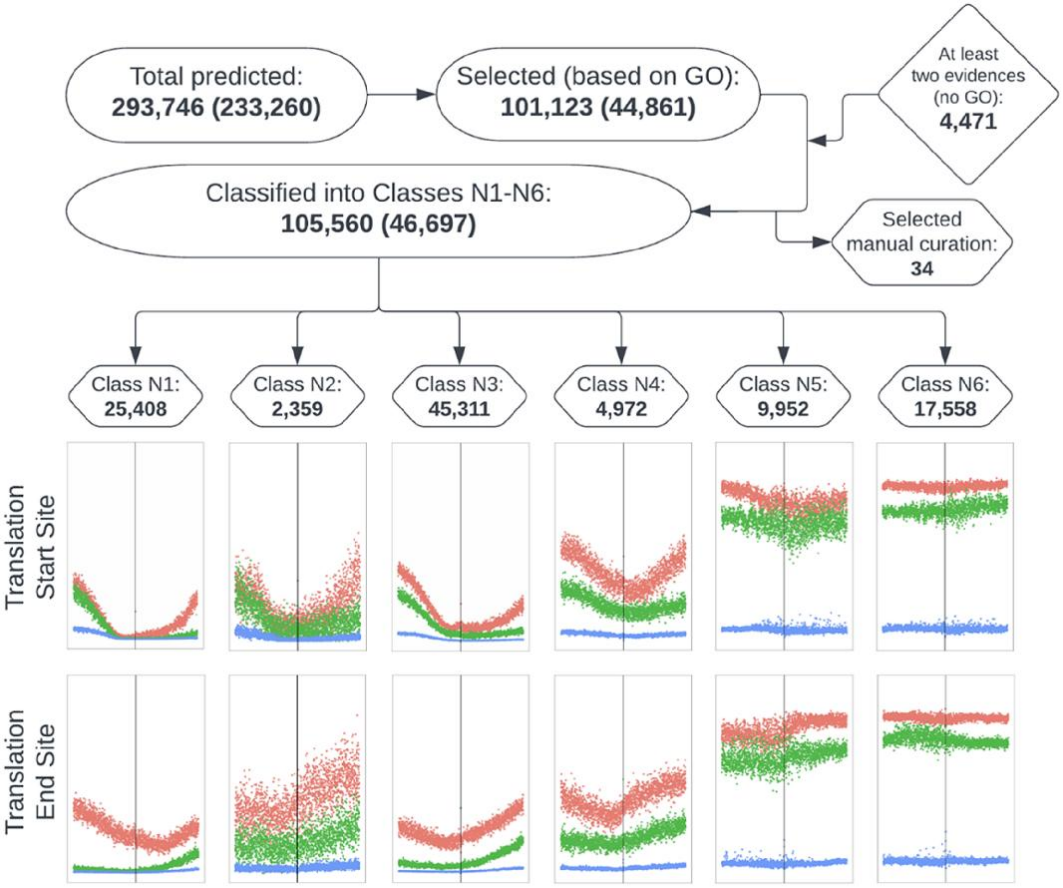
Number and methylation patterns of transcripts and gene models identified in the *E. guineensis* genome. The first number represents the count of transcripts, and the count of gene models is in parentheses. The transcripts that were identified using the Seqping and Mikado pipelines were selected based on their BLAST, Interproscan, and EggNOG annotations in OmicsBox. An additional 4,471 transcripts that did not have any GO annotation but had at least two independent support evidence (BLASTP, RNA-seq, and/or CAGE) were also selected for classification into six classes based on the supporting data available. Class N1, N2, N3, and N4 have evidence (CAGE and/or RNAseq) that the genes are expressed while Class N5 and N6 do not have any expression data support. Methylation patterns at the translation start and end sites of representative isoforms of each gene model in the six classes show that the genes that do not have expression data support have high levels of methylation. Red, green, and blue lines indicate the CG, CHG, and CHH methylation profiles of the isoforms, respectively.

Classification of gene models was based on RNA-Seq expression data from different tissues, CAGE expression data, and BLAST alignment results to Genbank's Liliopsida/monocotyledon library. Gene models were classified into six categories according to the level of independent data support for the genes: (N1) CAGE sequencing, RNA-Seq and BLAST, (N2) CAGE and either RNA-Seq or BLAST, (N3) RNA-Seq and BLAST, (N4) CAGE or RNA-Seq only, (N5) BLAST only, and (N6) in silico predicted genes not supported by experimental evidence (Fig. 6, Supplementary Table 7, Supplementary Table 8, Methods); 55.4% (25,884) and 72.0% (27,840) of *E. guineensis* and *E. oleifera* predicted genes have one or more sources of gene expression support, respectively (Fig. 6).

Cytosine methylation at translation start and end sites was calculated from whole genome bisulfite sequencing data derived from tissue-cultured mother palms (ortets) (Methods). Expressed genes have characteristic cytosine methylation patterns, whereby CG and CHG methylation tends to be low at the translation start and end sites relative to the gene body. These patterns are consistent with the gene expression-based classification of the gene models, whereby genes with CAGE and/or RNA-Seq supporting evidence (Classes N1, N2, N3, and N4) demonstrated these CG and CHG methylation characteristics, while genes with BLAST only (Class N5) or no experimental expression support (Class N6) exhibited dense CG and CHG methylation throughout the gene (Fig. 6). Furthermore, genes with no expression evidence (Classes N5 and N6) are enriched for housekeeping genes relative to those with expression evidence (Supplementary Fig. 10). Therefore, the latter classes of genes may represent potentially expressed genes that are epigenetically silenced in the tissues analyzed or pseudogenes that have been inactivated over evolutionary time. Comparisons of gene ontology characterization by biological process, molecular function, and cellular component demonstrate the general consistency of gene content between the two oil palm species (Supplementary Fig. 11). Ortholog analysis showed that the oil palm genes belong to 13,335 orthologous groups (Data S1).

### Genomic repeat element analyses

**Fig. 7.**
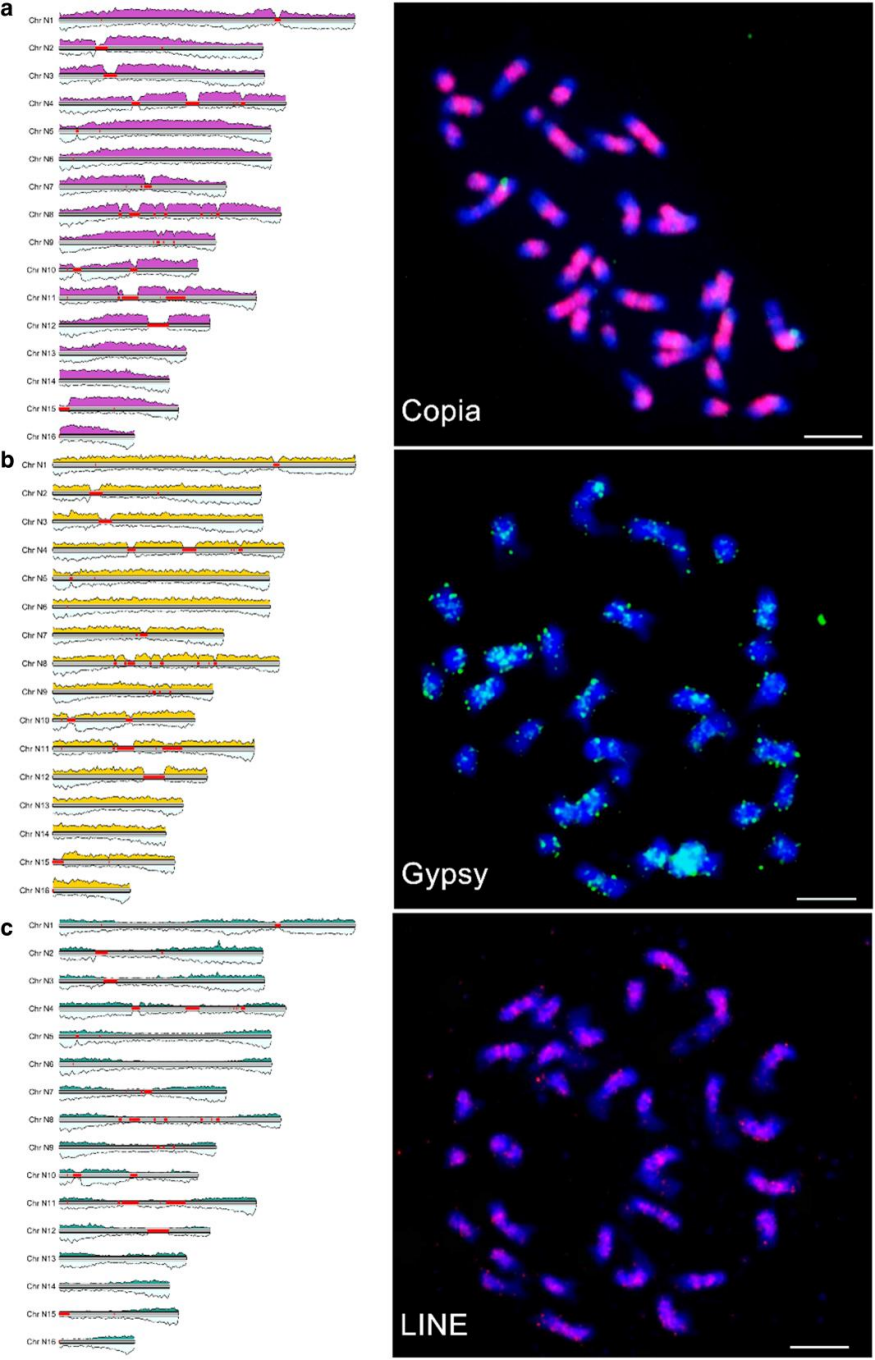
Repeat element distribution in *E. guineensis* chromosomes. The distributions of repeat elements were predicted by in silico analysis and independently confirmed by hybridization using (a) Copia, (b) Gypsy, and (c) LINE-like retrotransposon probes. Bar: 5 µm. Copia, Gypsy, and LINE densities are plotted on the left panel in magenta, yellow, and turquoise, respectively, while gene densities are in light blue. Red lines are gaps in the genome sequence.

Repeat content was analyzed in *E. guineensis*, *E. oleifera*, coconut, and date palm genomes (Methods). Localization of Ty1/Copia, Gypsy, and LINE repetitive elements was consistent between in silico localization and hybridization of *E. guineensis* chromosomes with FISH probes (Fig. 7). Ty1/Copia elements (of the Angela lineage) are concentrated at the pericentromeric regions of each chromosome, as well as the non-centromeric constricted region on chromosome 1. In contrast, Gypsy elements are more broadly distributed with more granular staining patterns across each chromosome, consistent with Wang *et al.* (2023). LINE elements, which are correlated with genes, are broadly distributed, with a more telomere-proximal distribution relative to Copia elements in both the DNA sequence and cytogenetic analyses. Correlations between specific repeat elements and gene density were analyzed in both species relative to the gene prediction classes described in Fig. 6 (Supplementary Fig. 12). In *E. guineensis*, strong negative correlations exist between specific LTR family repeats (Ngaro, Gypsy, and Copia) and gene classes with expression evidence and lower translation start and end site DNA methylation densities, but not for densely methylated gene predictions without expression evidence (Supplementary Fig. 12a), consistent with the reported negative correlations between repetitive sequences and gene density observed in the reference *dura* genome assembly (Wang *et al*. 2023). Conversely, other repeat types (simple and low-complexity repeats, LINE, RC/Helitron, DNA/MULE-MuDR, and others) are positively correlated with gene density for classes N1, N2, N3, and N4. Class N6 gene predictions have low levels of correlation with most of the repeat types, the highest being positive correlations with Gypsy elements. Distributions of genes and repeats are generally similar in the two oil palm species (Supplementary Fig. 12). However, in contrast to *E. guineensis*, *E. oleifera* class N6 gene densities are negatively correlated with Ngaro, Gypsy, and Copia elements and positively correlated with other repeat types. Neither species has correlations between any gene prediction class and LTR/Caulimovirus elements. Oil palm genes (all classes combined) have stronger positive correlations with multiple repeat types (simple repeats, satellites, LINE, RC/Helitron, DNA/MULE-MuDR, and others) than do coconut and date palm genes (Supplementary Fig. 13). Negative correlations between genes and LTR/Copia repeats are consistent across oil palm, coconut, and date palm. Negative correlations between genes and LTR/Ngaro repeats are present in the oil palm genome, but not in coconut or date palm. Finally, the negative correlation between genes and LTR/Gypsy repeats is higher in date palms than in oil palm or coconut.

Together, these findings indicate that since the divergence of date palm and oil palm ∼65 Mya (Singh, Ong-Abdullah, *et al*. 2013; He *et al*. 2015), multiple repeat elements have undergone enrichment in genic regions in both coconut and oil palm (Wang *et al*. 2023). This enrichment has continued in oil palm relative to coconut since their divergence 45 Mya (He *et al*. 2015). As shown in chromosome-scale karyoplots of various repeat family densities, this enrichment appears to represent a redistribution of specific elements such as LINEs (Supplementary Fig. 14), and simple and low-complexity repeats (Supplementary Fig. 15) in gene-rich telomere-proximal regions in oil palm and coconut relative to date palm, rather than genome-wide amplification of these elements. Other repeat families (LTR/Copia (Supplementary Fig. 16) and LTR/Gypsy (Supplementary Fig. 17)) instead appear to have amplified substantially in oil palm and coconut relative to date palm, with Copia elements enriched in centromeric regions (Fig. 7, Supplementary Fig. 16) and Gypsy elements more broadly distributed (Fig. 7, Supplementary Fig. 17).

## Conclusions

The two oil palm reference genomes reported here represent (i) substantial improvements in contiguity, completeness, gene annotation and repeat composition relative to previously reported *E. guineensis* reference genome assemblies, and (ii) the first chromosome-scale reference genome of *E. oleifera.* Both references share very similar genome quality metrics, making them suitable for continued comparative genomics and association studies. A critical application of these genomics tools will be to advance the introgression of favorable *E. oleifera* alleles into elite *E. guineensis* x *E. oleifera* interspecific hybrid lines. Comparative genomic analyses demonstrate high structural genomic conservation between the two species and point out low abundance and potentially important genetic differences between the species. These findings may lead to the identification of valuable markers associated with traits of interest that may be exploited to advance the development of elite interspecific hybrid lines and will serve as a foundation for the development of a high-resolution oil palm pan-genome including sequence and structural variations across the oil palm germplasm.

## Supplementary Material

jkae135_Supplementary_Data

## Data Availability

The *E. guineensis* and *E. oleifera* genome sequences are available for download at the Genomsawit (http://genomsawit.mpob.gov.my) website and have been registered at NCBI under the BioProject accessions PRJNA 192219 (accession ASJS00000000) and PRJNA 183707 (accession ASIR00000000), respectively. The script for the classification of the gene models is available on the Genomsawit website and on Github (https://github.com/kythol/Low_OilPalm2024). Supplemental material available at G3 online.
