# Supplementary material for: Chromosome-scale Elaeis guineensis and E. oleifera assemblies: comparative genomics of oil palm and other Arecaceae
Source: G3 (Bethesda). 2024 Jun 26;14(9):jkae135. doi: 10.1093/g3journal/jkae135 (PMC11373658; doi:10.1093/g3journal/jkae135)
Supplement: jkae135_Supplementary_Data [file jkae135_supplementary_data.zip › Supplemental_Material_G3-2024-405041.pdf]

Supplementary Information for

Chromosome-scale *Elaeis guineensis* and *E. oleifera* assemblies: Comparative genomics of oil palm and other Arecaceae

Eng-Ti Leslie Low *et al.*

Table of Contents

Figure S1. EG11 pseudochromosomes……………………………………………………………………………………3

Figure S2. *E. guineensis* reference genome (EG11) pseudochromosome assembly using AllMaps……4

Figure S3. Merqury quality control analyses of EG11 and EO12.1 assemblies………………………………12

Figure S4. Hi-C assembly error in Chromosome 5……………………………………………………………………13

Figure S5. Chromosome fusions involved in descending dysploidy from 18 to 16 chromosomes in the evolution of oil palm ……………………………………………………………………………………………………14

Figure S6. Comparative genomics of oil palm species to banana…………………………………………………15

Figure S7. Representative PCR validation results for small structural variants between oil palm species………16

Figure S8. SyRI-predicted genomic inversions and duplications between *E. guineensis* and *E. oleifera* ………17

Figure S9. SyRI-predicted genomic translocations between *E. guineensis* and *E. oleifera*………………18

Figure S10. Fisher's Exact Test comparison of EG11 gene models ………………………………………………19

Figure S11. Gene ontology classifications of the oil palm selected gene models……………………………20

Figure S12. Oil palm genes and repeat elements …………………………………………………………………..23

Figure S13. Correlation analysis of EG/EO/Coconut/Date Palm repeat structures and gene models………25

Figure S14. *E. oleifera*, *C. nucifera* and *P. dactylifera* LINE karyoplots……………………………………………..26

Figure S15. *E. oleifera, C. nucifera and P. dactylifera* simple and low complexity repeat karyoplots..27

Figure S16. *E. oleifera, C. nucifera* and *P. dactylifera* copia karyoplots………………………28

Figure S17. *E. oleifera, C. nucifera* and *P. dactylifera* gypsy karyoplots ……………………….29

Table of Contents (continued)

Table S1. Genome assembly statistics relative to previously published *E. guineensis* and *E. oleifera* assemblies ………30

Table S2. EG11 and EO12.1 transcript statistics……………….…………………………………………………….………….31

Table S3. Summary of pseudochromosome naming between EG5 and EG11/EO12.1…………………………32

Table S4. Summary of EG11 guided edits to EO12.1……………………………………….……………..……..………….33

Table S5. Candidate indel/duplication/translocation structural variants with PCR support evidence…..34

Table S6. Structural variants predicted by SyRI comparisons of EG11 and EO12.1……………………………….37

Table S7. Gene model annotation summary ………………………………………….…………………………………..………….38

Table S8. Gene model prediction summary ……………………………………….………………………………..…….…..…….39

Table S9. Primer pairs for amplification of retrotransposon probes used in the Fluorescence *in situ* hybridization (FISH) analyses……..………40

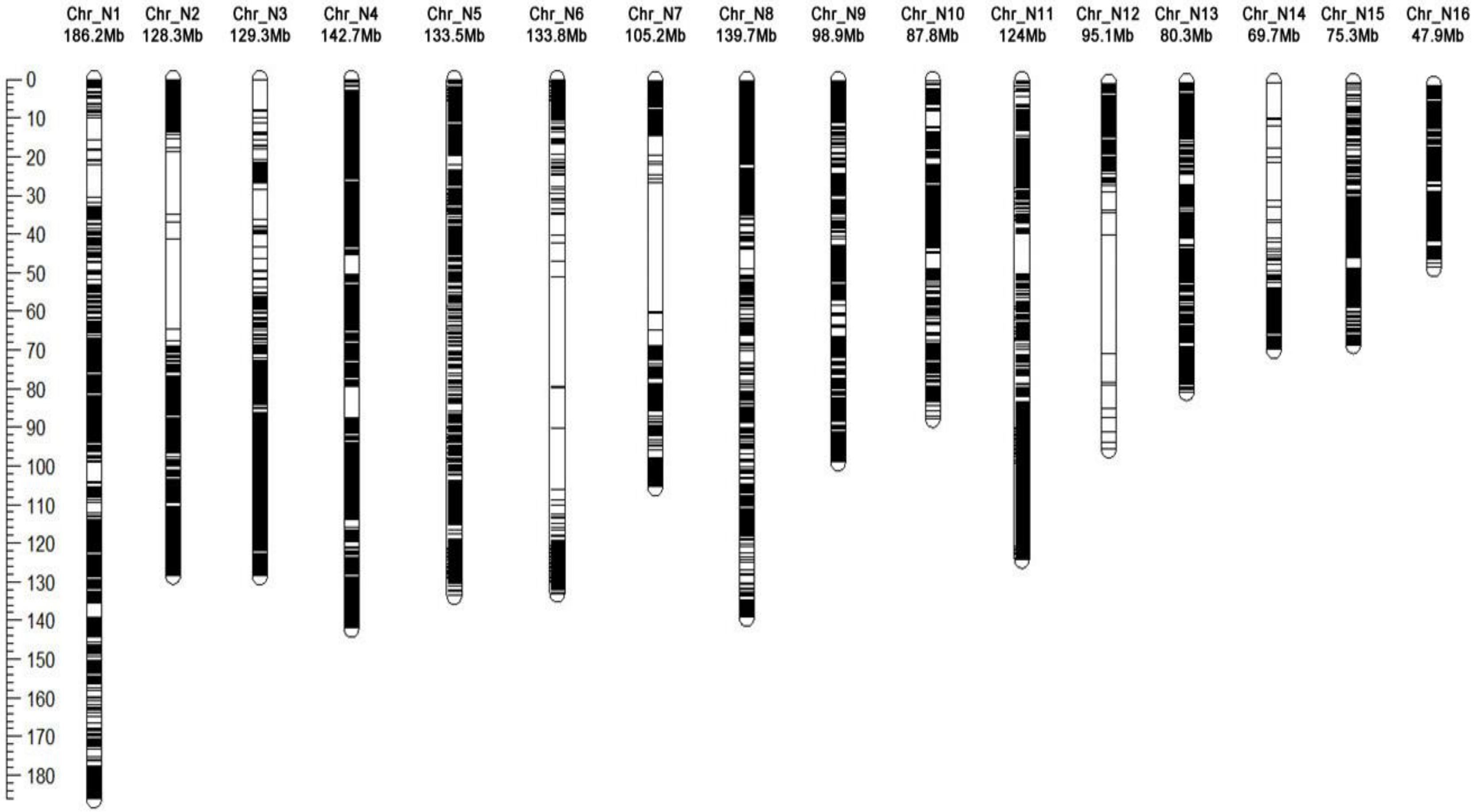

Figure S1. EG11 pseudochromosomes. Dark regions show the distribution of markers from the P2, T128 and PUP genetic maps that were used to generate the EG11 build. Chromosome 2 in Singh, Ong-Abdullah, *et al.* (2013) has been reassigned as Chromosome N1, as it is the longest chromosome that has been verified cytogenetically.

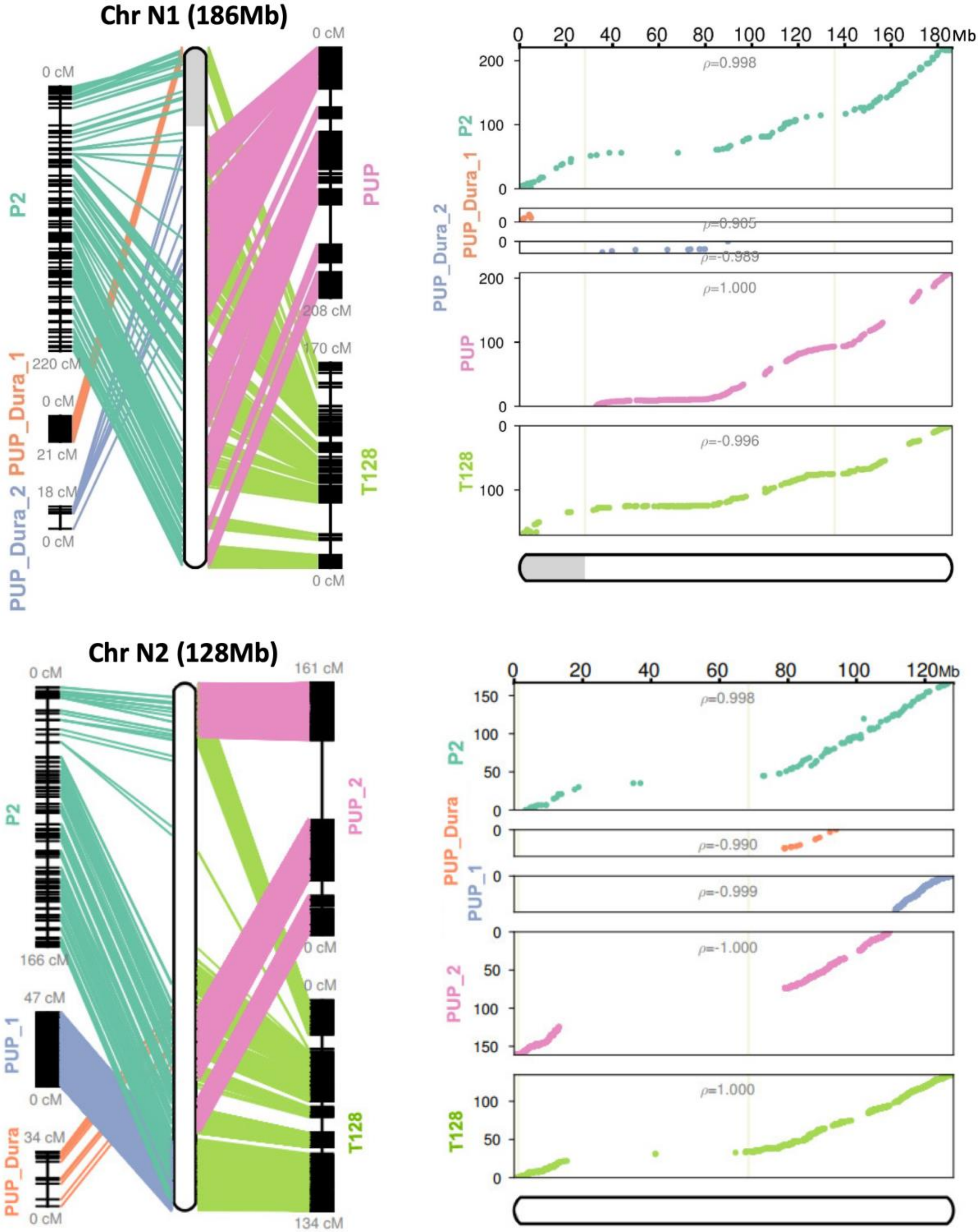

Figure S2. ***E. guineensis*** **reference genome (EG11) pseudochromosome assembly using AllMaps.** For each pseudochromosome, flanking T128, P2 and PUP genetic map positions are shown with lines connecting to the central physical positions on the assembled pseudochromosomes (left panel). For each linkage map, scatter plots of the physical position on the chromosome (x-axis) versus the genetic map location (y-axis) are shown (right panels). Subgroups are labelled as _1 and _2.

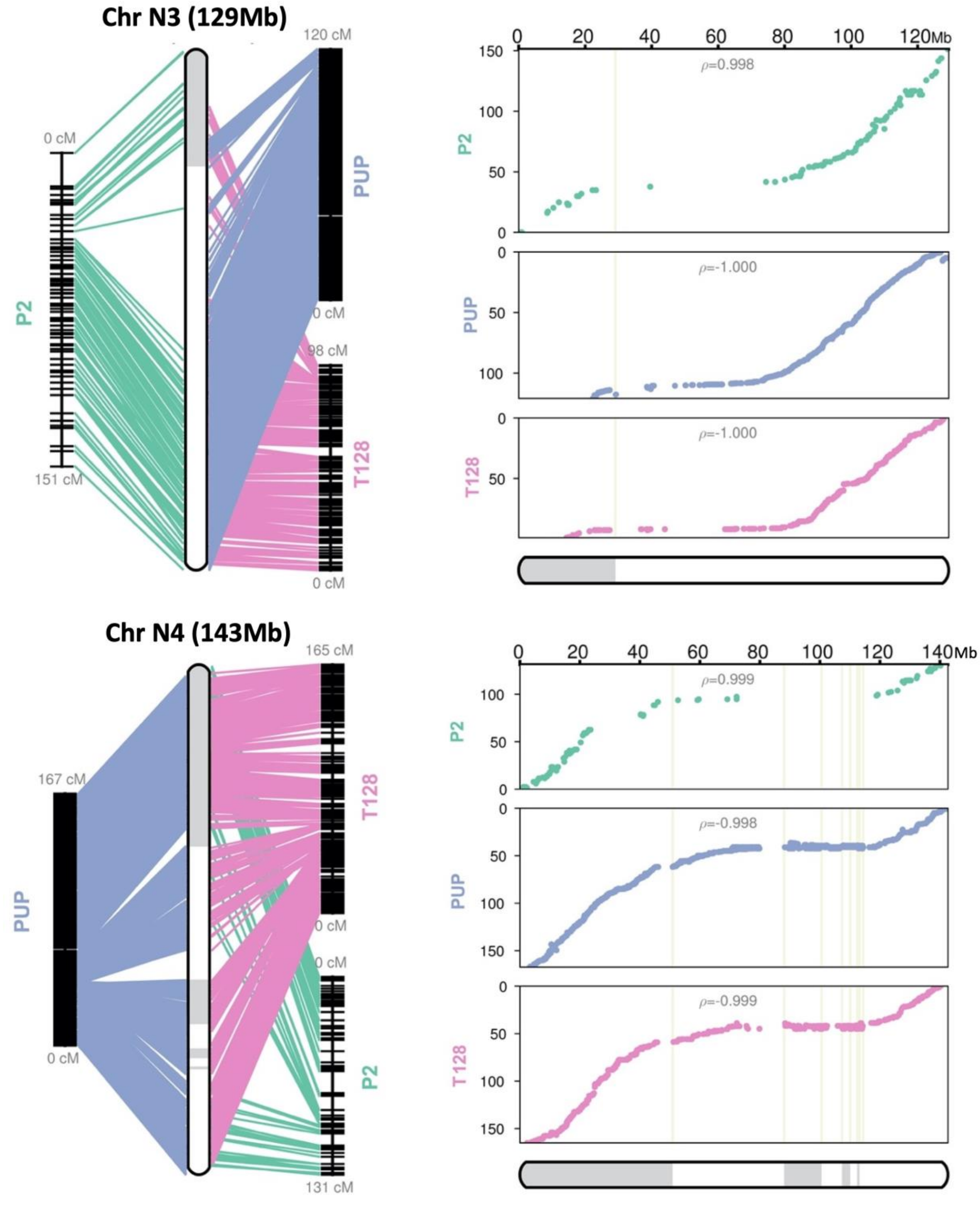

Figure S2 continued. ***E. guineensis*** **reference genome (EG11) pseudochromosome assembly using AllMaps.** For each pseudochromosome, flanking T128, P2 and PUP genetic map positions are shown with lines connecting to the central physical positions on the assembled pseudochromosomes (left panel). For each linkage map, scatter plots of the physical position on the chromosome (x-axis) versus the genetic map location (y-axis) are shown (right panels). Subgroups are labelled as _1 and _2.

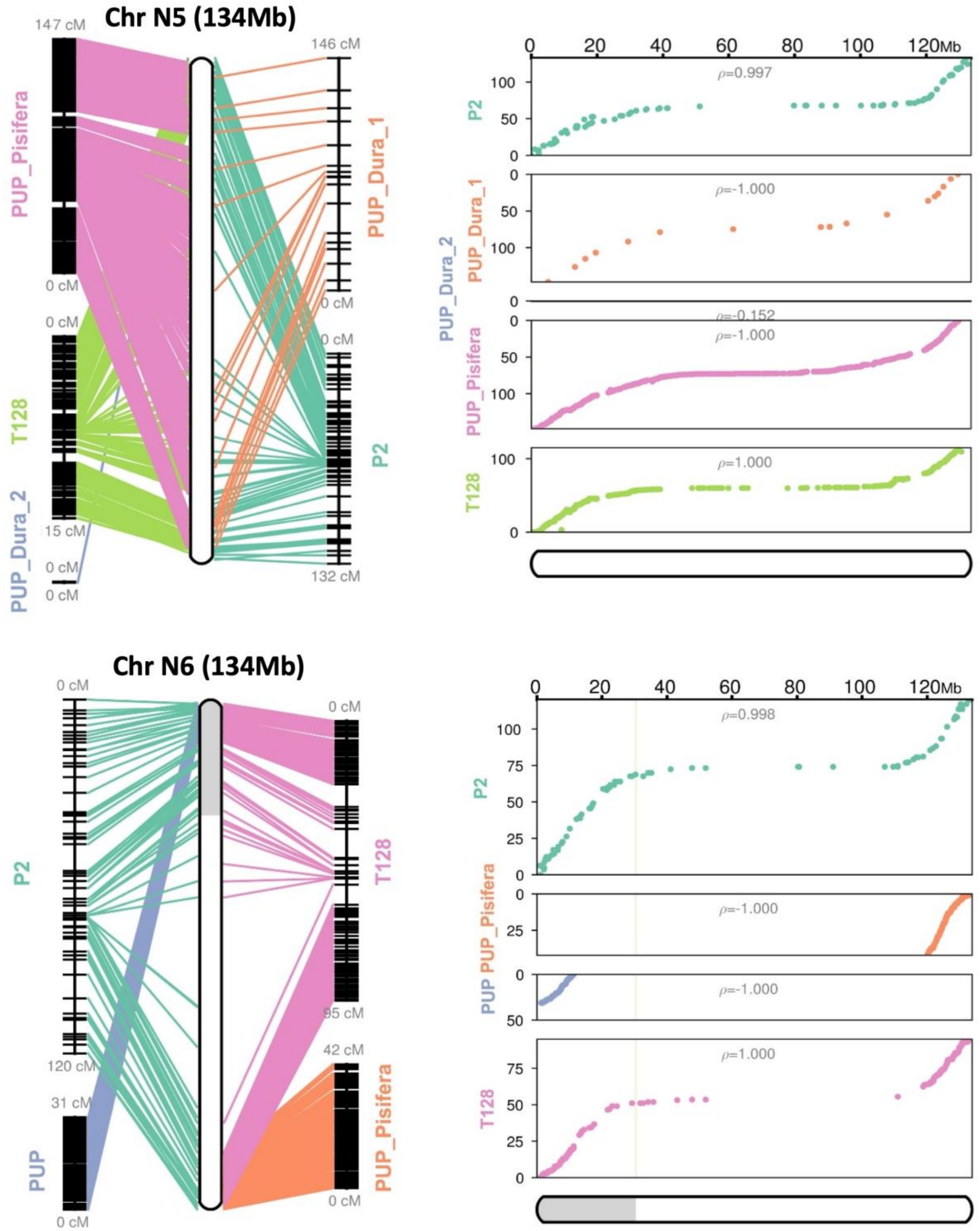

Figure S2 continued. ***E. guineensis*** **reference genome (EG11) pseudochromosome assembly using AllMaps.** For each pseudochromosome, flanking T128, P2 and PUP genetic map positions are shown with lines connecting to the central physical positions on the assembled pseudochromosomes (left panel). For each linkage map, scatter plots of the physical position on the chromosome (x-axis) versus the genetic map location (y-axis) are shown (right panels). Subgroups are labelled as _1 and _2.

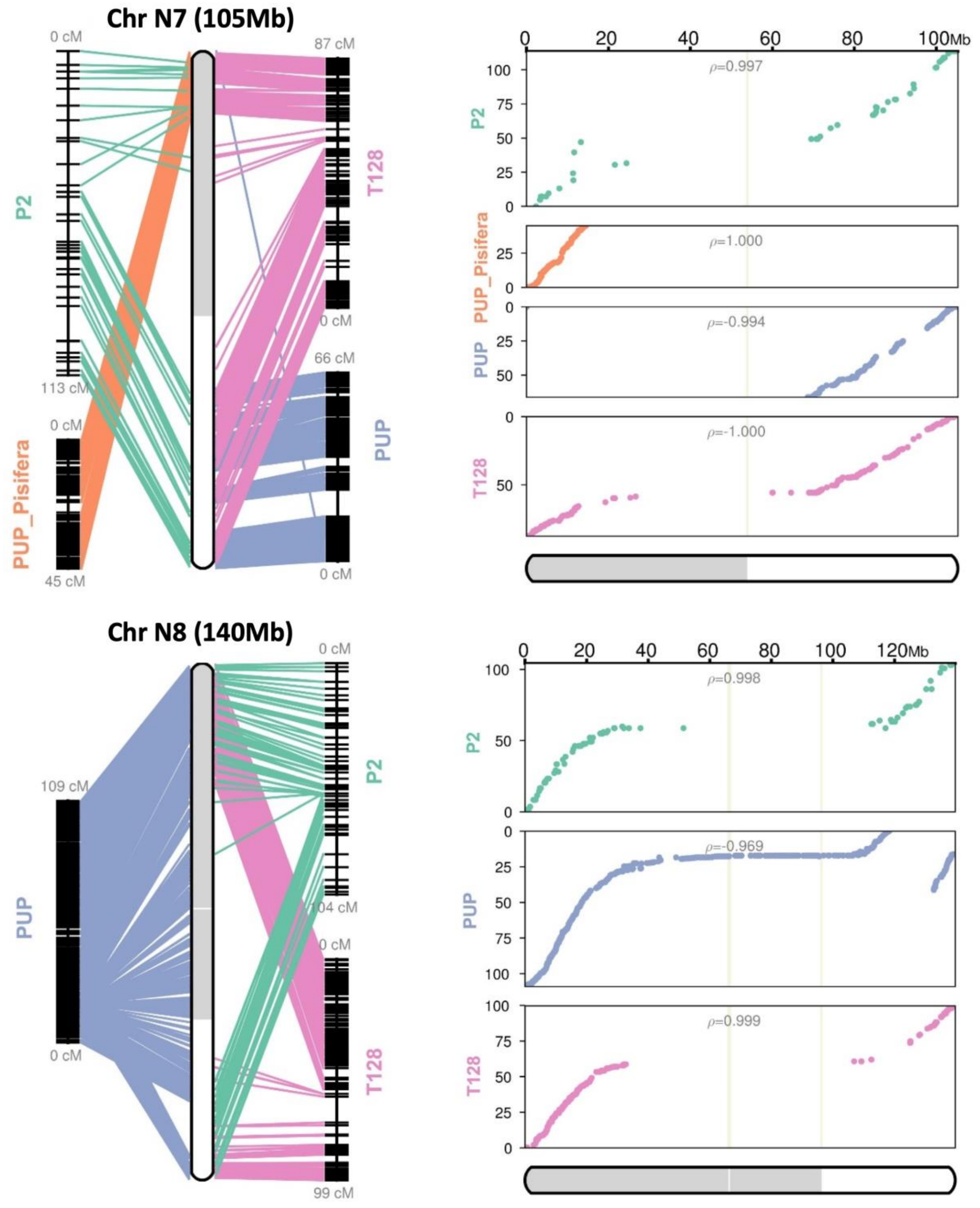

Figure S2 continued. ***E. guineensis*** **reference genome (EG11) pseudochromosome assembly using AllMaps.** For each pseudochromosome, flanking T128, P2 and PUP genetic map positions are shown with lines connecting to the central physical positions on the assembled pseudochromosomes (left panel). For each linkage map, scatter plots of the physical position on the chromosome (x-axis) versus the genetic map location (y-axis) are shown (right panels). Subgroups are labelled as _1 and _2.

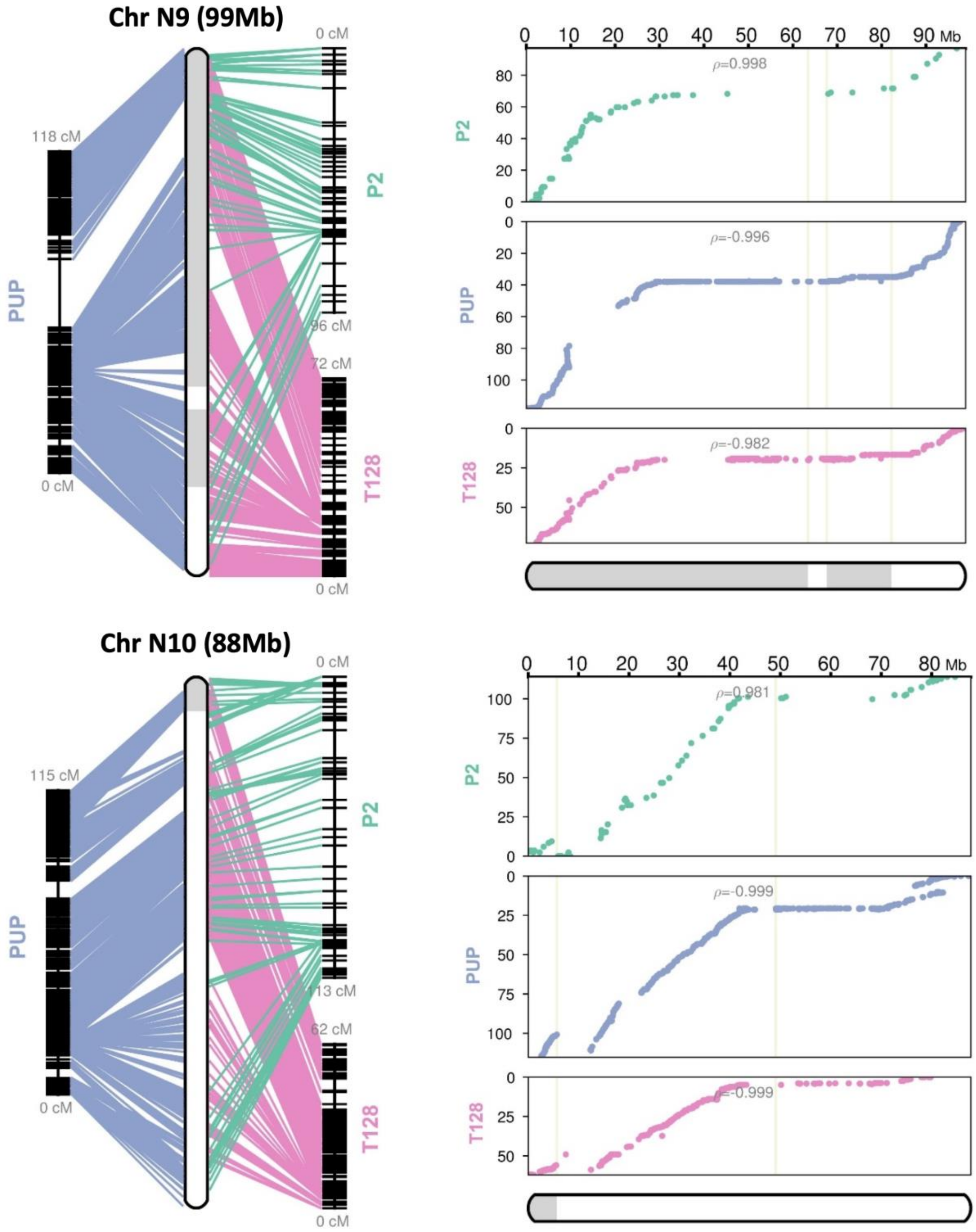

Figure S2 continued. ***E. guineensis*** **reference genome (EG11) pseudochromosome assembly using AllMaps.** For each pseudochromosome, flanking T128, P2 and PUP genetic map positions are shown with lines connecting to the central physical positions on the assembled pseudochromosomes (left panel). For each linkage map, scatter plots of the physical position on the chromosome (x-axis) versus the genetic map location (y-axis) are shown (right panels). Subgroups are labelled as _1 and _2.

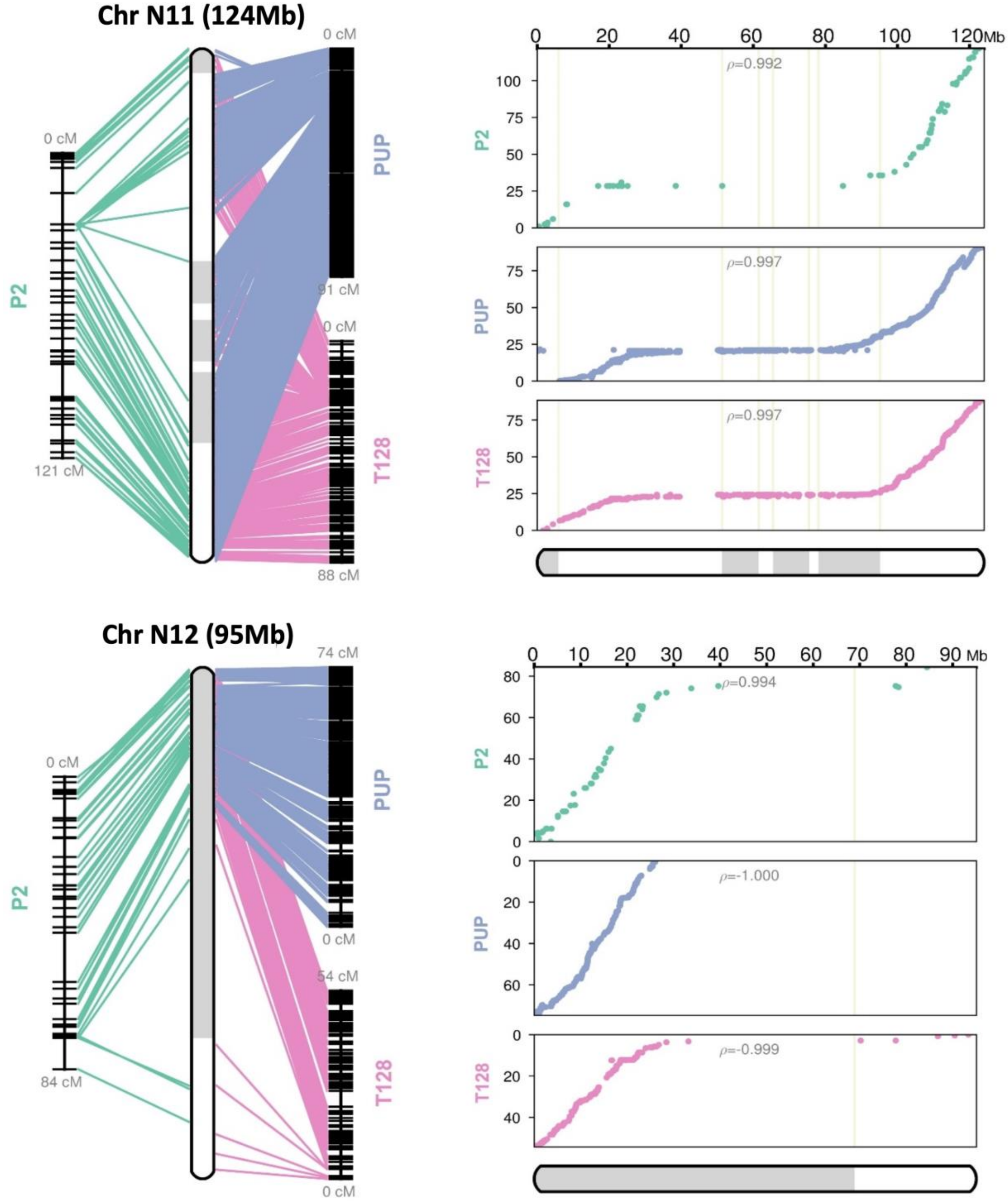

Figure S2 continued. ***E. guineensis*** **reference genome (EG11) pseudochromosome assembly using AllMaps.** For each pseudochromosome, flanking T128, P2 and PUP genetic map positions are shown with lines connecting to the central physical positions on the assembled pseudochromosomes (left panel). For each linkage map, scatter plots of the physical position on the chromosome (x-axis) versus the genetic map location (y-axis) are shown (right panels). Subgroups are labelled as _1 and _2.

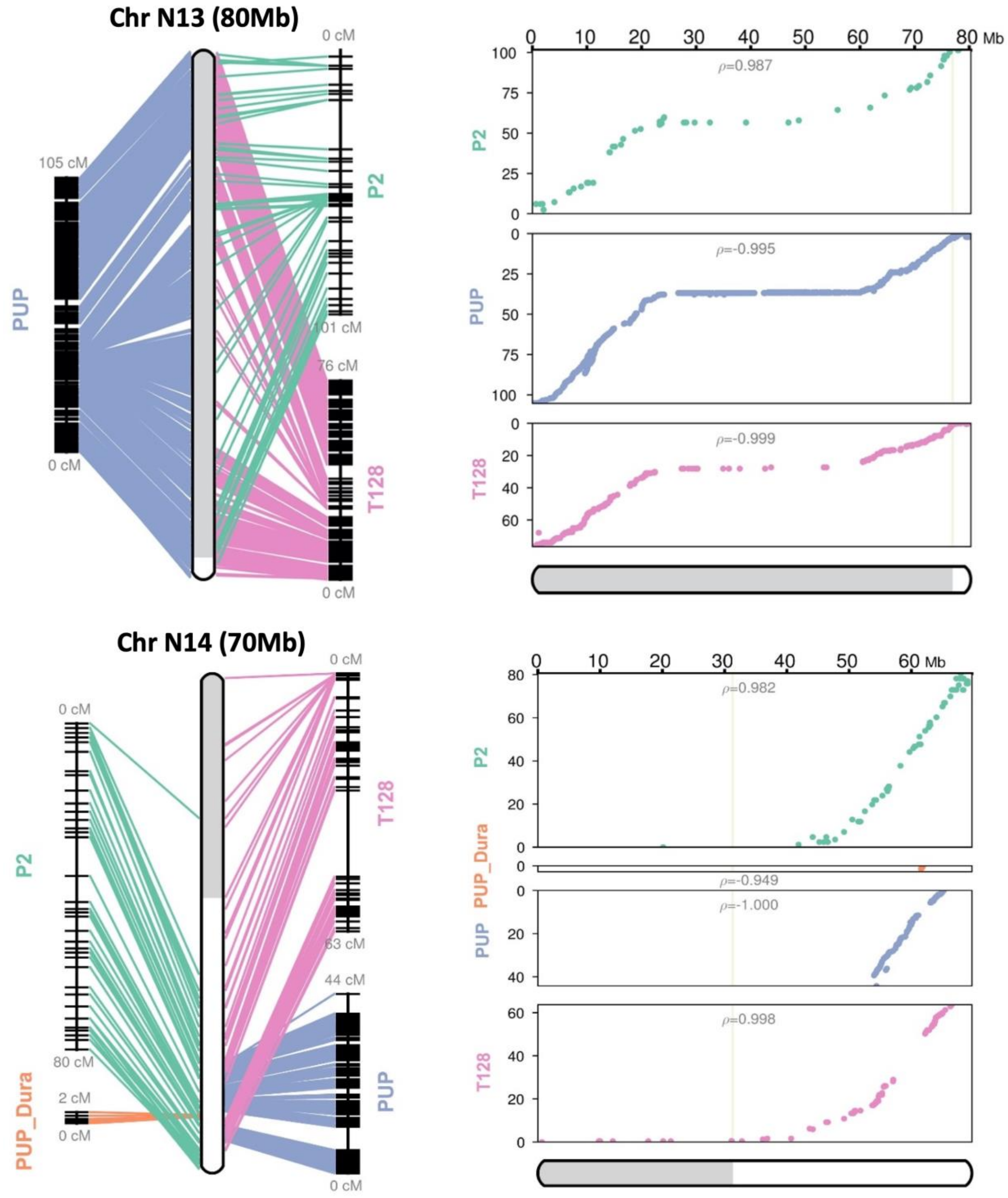

Figure S2 continued. ***E. guineensis*** **reference genome (EG11) pseudochromosome assembly using AllMaps.** For each pseudochromosome, flanking T128, P2 and PUP genetic map positions are shown with lines connecting to the central physical positions on the assembled pseudochromosomes (left panel). For each linkage map, scatter plots of the physical position on the chromosome (x-axis) versus the genetic map location (y-axis) are shown (right panels). Subgroups are labelled as _1 and _2.

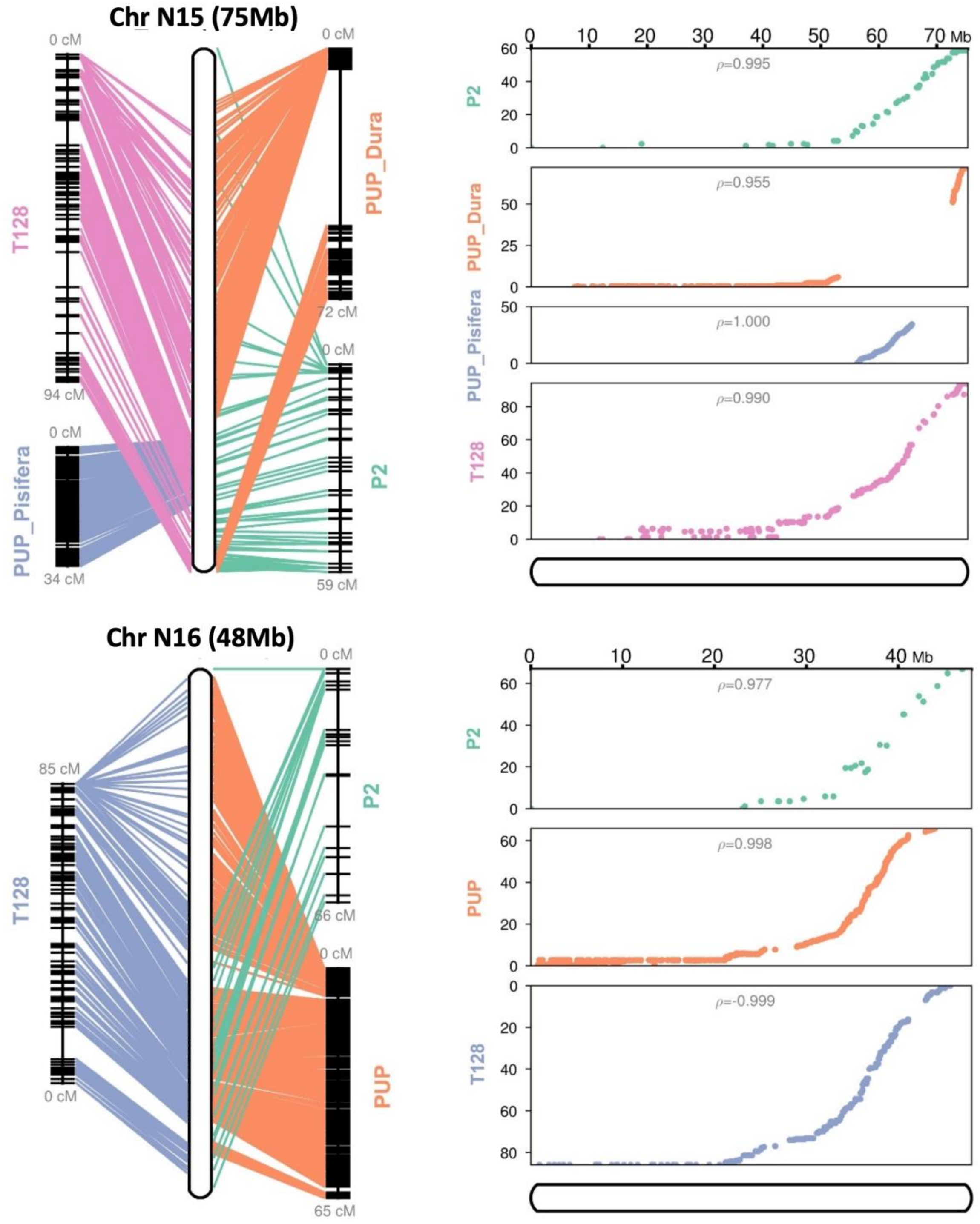

Figure S2 continued. ***E. guineensis*** **reference genome (EG11) pseudochromosome assembly using AllMaps.** For each pseudochromosome, flanking T128, P2 and PUP genetic map positions are shown with lines connecting to the central physical positions on the assembled pseudochromosomes (left panel). For each linkage map, scatter plots of the physical position on the chromosome (x-axis) versus the genetic map location (y-axis) are shown (right panels). Subgroups are labelled as _1 and _2.

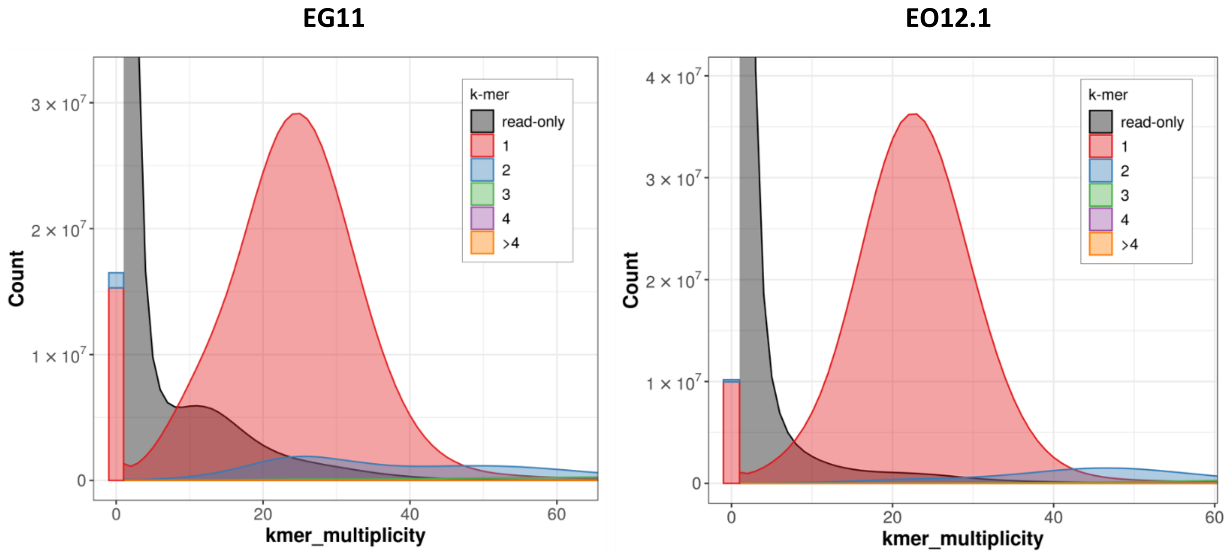

Figure S3. Merqury quality control analyses of EG11 and EO12.1 assemblies. Merqury analyses were performed as described Rhei *et al.* (2020) to produce EG11 (left) and EO12.1 (right) copy number spectrum plots. k-mers from Illumina whole genome short read sequencing of each oil palm species are counted using Meryl. The specta-cn plots represent the multiplicity of each k-mer found in the short-read dataset and colors it by the number of times it is found in the assembly. The grey line represents k-mers found only in the short-read data. Red lines represent k-mers found once in the assembly. For both assemblies, the peak of these single copy k-mers is close to the 30X Illumina short read sequencing depth. Blue lines represent k-mers found twice in the assembly. Vertical bars at the x-axis origin represent reads that are found in the assembly, but not the short-read set, and estimate base error in the assembly.

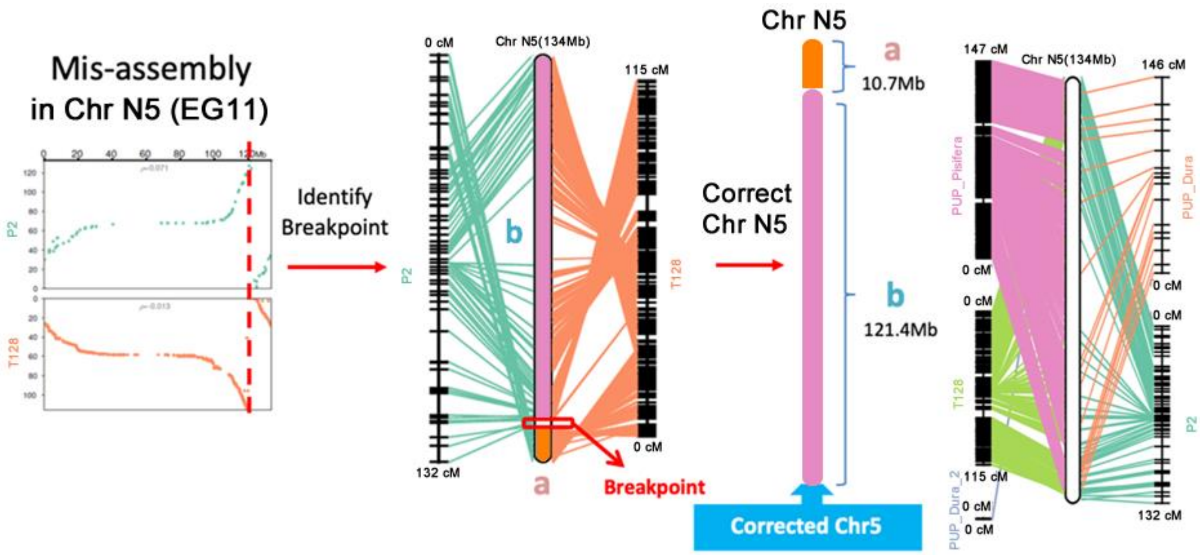

Figure S4. Hi-C assembly error in Chromosome 5. The error, identified using genetic maps from three mapping populations (T128, P2 and PUP), resulted in the merger of the sequences from both ends of chromosome 5. The breakpoint to correct the error was identified via the mapping of genetic markers, contigs of earlier assemblies and the identification of telomeric sequences. Fragment (a) from the end of the chromosome was cut at the join of 2 contigs, and merged with the sequences at the opposite end of the chromosome (b). The placement was verified using markers from the genetic maps.

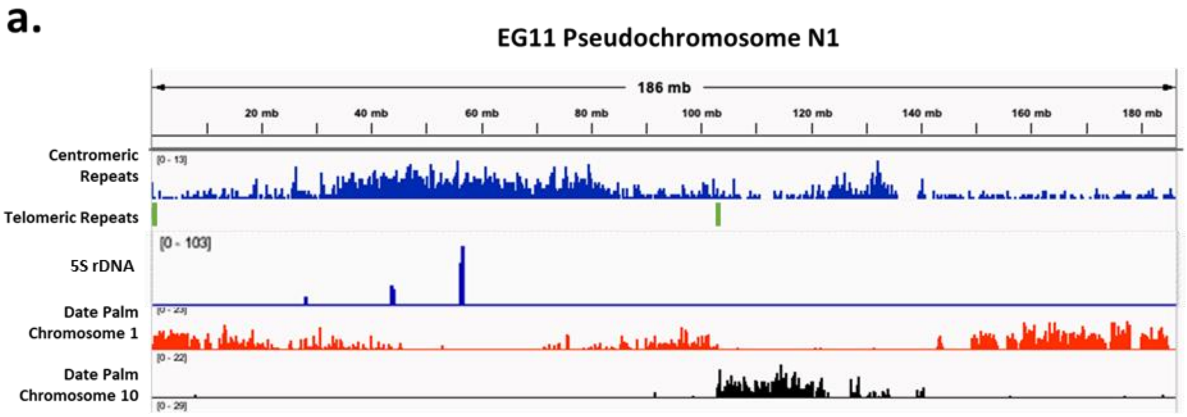

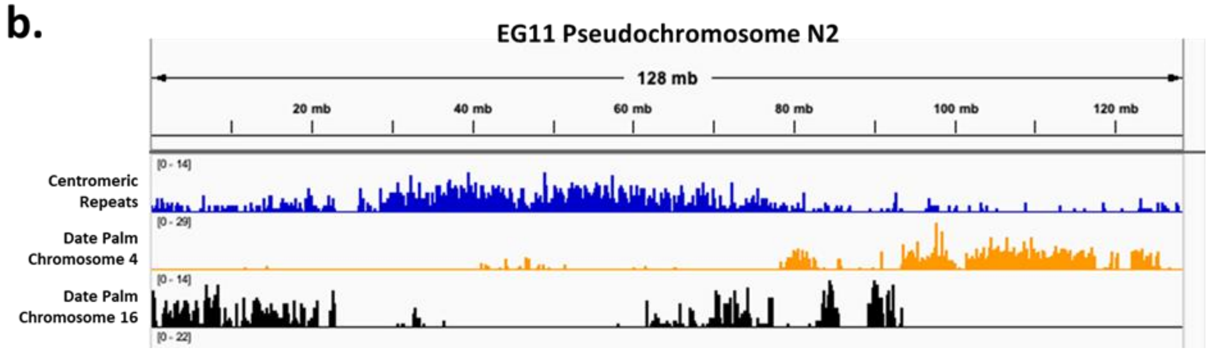

Figure S5. Chromosome fusions involved in descending dysploidy from 18 to 16 chromosomes in the evolution of oil palm. a. The scale of EG11 pseudochromosome N1 (186 Mb) is shown at the top of the panel. Centromeric repeat sequences were BLASTED to EG11 N1 and alignment densities are plotted in blue. Telomeric repeat sequence were BLASTED to EG11 N1 and the interstitial hit is shown by a green bar. Consensus 5S rDNA sequences were BLASTED to EG11 N1 and hits are shown by blue bars labeled 5S rDNA. BLAST hit densities to date palm reference genome chromosomes 1 and 10 are shown in red and black, respectively. The patterns are consistent with an evolutionary descending dysploidy event leading to the fusion of ancestral chromosome 1 with the inserted date palm chromosome 10 by a nested chromosome fusion event. **b.** The scale of EG11 pseudochromosome N2 (128 Mb) is shown at the top of the panel. Centromeric repeat sequences were BLASTED to EG11 N2 and alignment densities are plotted in blue. BLAST hit densities to date palm reference genome chromosomes 4 and 16 are shown in orange and black, respectively. The patterns are consistent with an evolutionary descending dysploidy event leading to the fusion of oil palm chromosome 2 (syntenic with date palm chromosome 4) with the date palm chromosome 16 by an end-to-end fusion event. Overlaps in date palm chromosome 4 and 16 alignments with oil palm chromosome 2 may be the result of intrachromosomal recombination occurring after the fusion event. Interstitial telomeric repeats are not detected in oil palm pseudochromosome 2.

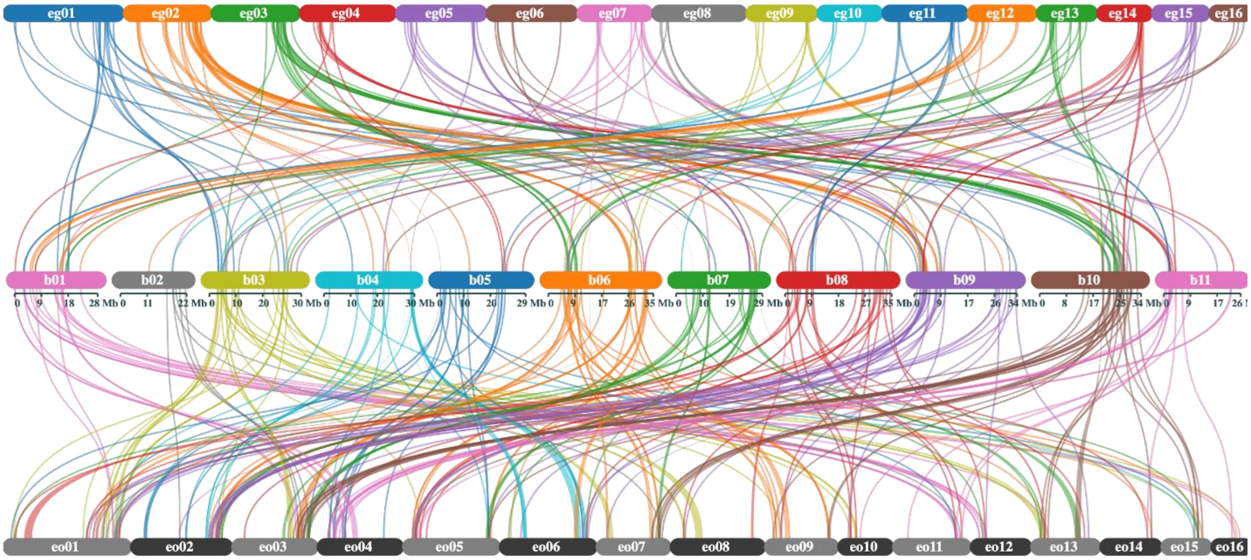

Figure S6. Comparative genomics of oil palm species to banana. *E. guineensis*, *E. oleifera* and *Musa acuminata* (banana) gene order synteny. EG11 pseudochromosomes (top row) and EO12.1 pseudochromosomes (bottom row) are depicted in order from chromosome 1 to 16. The 11 pseudochromosomes of the banana reference genome (middle row) are depicted in order from chromosome 1 to 11. Lines represent regions of gene order homoeologous relationships.

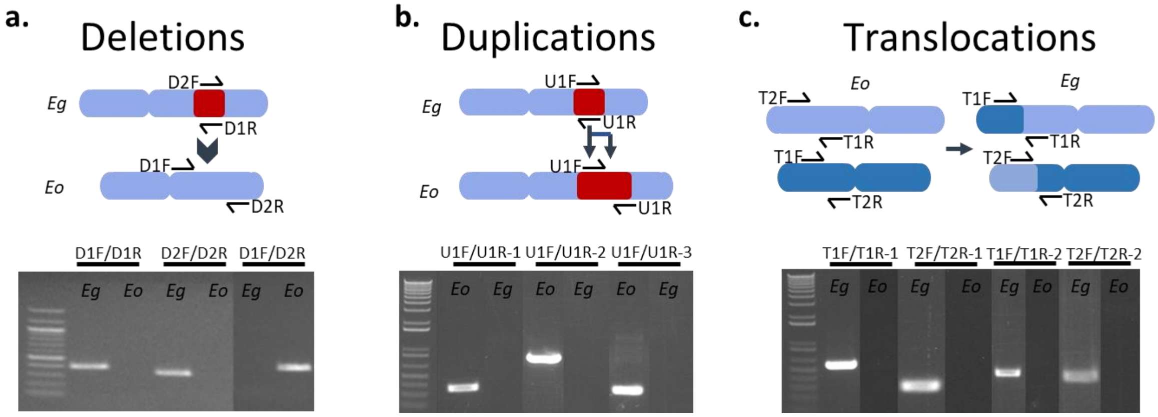

Figure S7. Representative PCR validation results for small structural variants between oil palm species. Example chromosome and PCR primer design diagram is shown at the top. **a.** Primers within (D2F and D1R) and primers flanking (D1F and D1R) predicted deletions in *E. oleifera* relative to *E. guineensis* were designed for each deletion candidate. Validated *E. oleifera* deletions amplify from *E. guineensis*, but not *E. oleifera*, genomic DNA. Three validated deletions are shown. **b.** Primers at the outer flanks of predicted duplications in *E. oleifera* relative to *E. guineensis* were designed for each duplication candidate (U1F and U1R). Validated *E. oleifera* duplications amplify a band of the predicted duplication size from *E. oleifera*, but not *E. guineensis*, genomic DNA. Three validated duplications are shown. **c.** Primers flanking predicted boundaries of candidate translocations were designed. Validated translocations amplify with primer pair T1F and T1R from *E. guineensis*, but not *E. oleifera*, genomic DNA because the primers are in proximity only on the *E. guineensis* chromosome. T2F and T2R amplify from *E. guineensis*, but not *E. oleifera*, genomic DNA because the primers are in proximity only on the *E. guineensis* chromosome. Two validated translocations are shown.

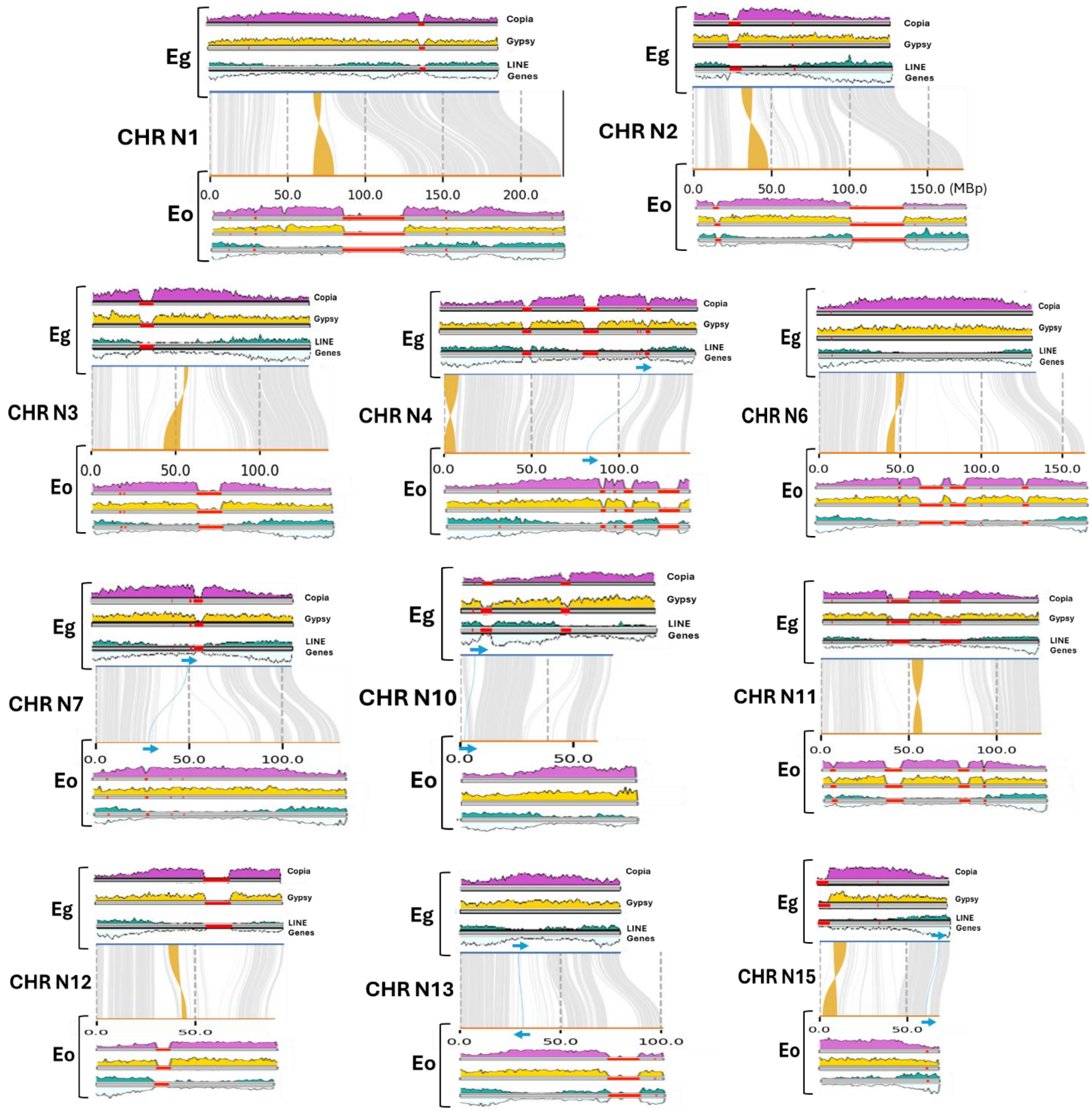

Figure S8. SyRI-predicted genomic inversions and duplications between *E. guineensis* and *E. oleifera.* Each panel compares a given EG11 pseudochromosome (Eg) to the syntenic pseudochromosome of EO12.1 (Eo). Karyoplots depicting Copia (magenta), Gypsy (yellow) and LINE (turquoise) and gene (light blue) density are shown above (EG11) or below (EO12.1) each pseudochromosome comparison plot. Red bars in karyoplots represent gaps. Syntenic regions (grey lines), inversions (orange objects) and duplications (blue lines) are shown in each pseudochromosome comparison plot. Blue arrows indicate directionality of duplicated regions. X-axis labels are in megabase pairs. Genomic coordinates for predicted inversions and duplications are provided in Table S6.

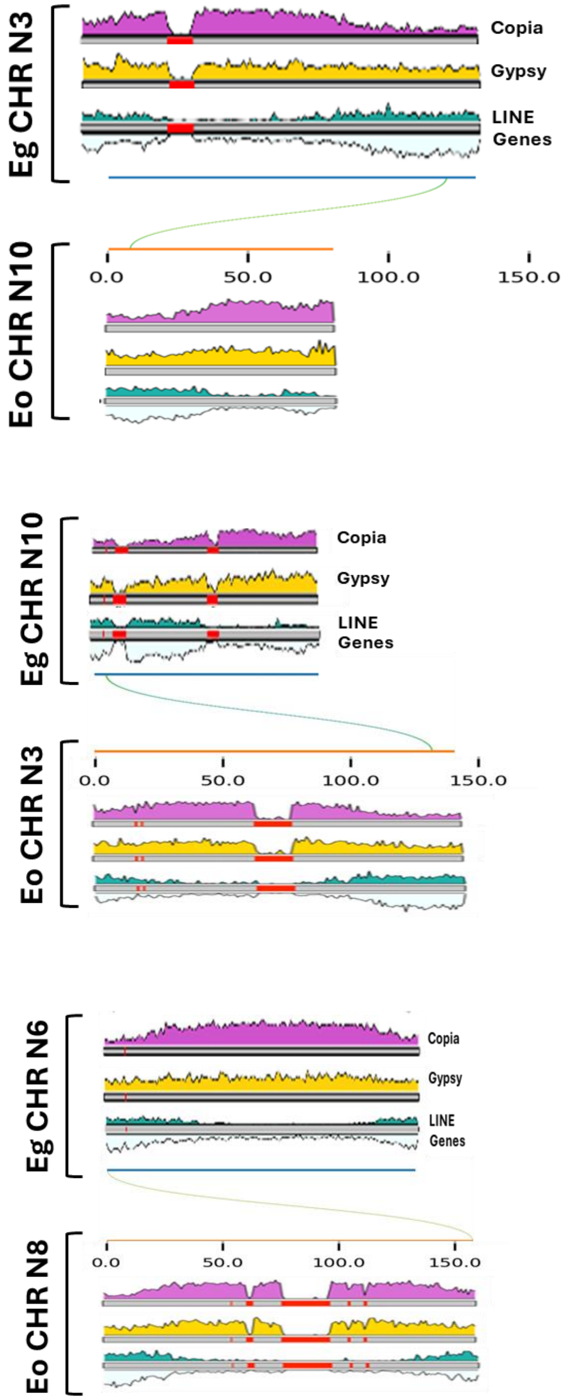

Figure S9. SyRI-predicted genomic translocations between *E. guineensis* and *E. oleifera.* Karyoplots are shown as described in Figure S8. Predicted translocations are depicted by green lines. X-axis labels are in megabase pairs. Genomic coordinates for predicted translocations are provided in Table S6.

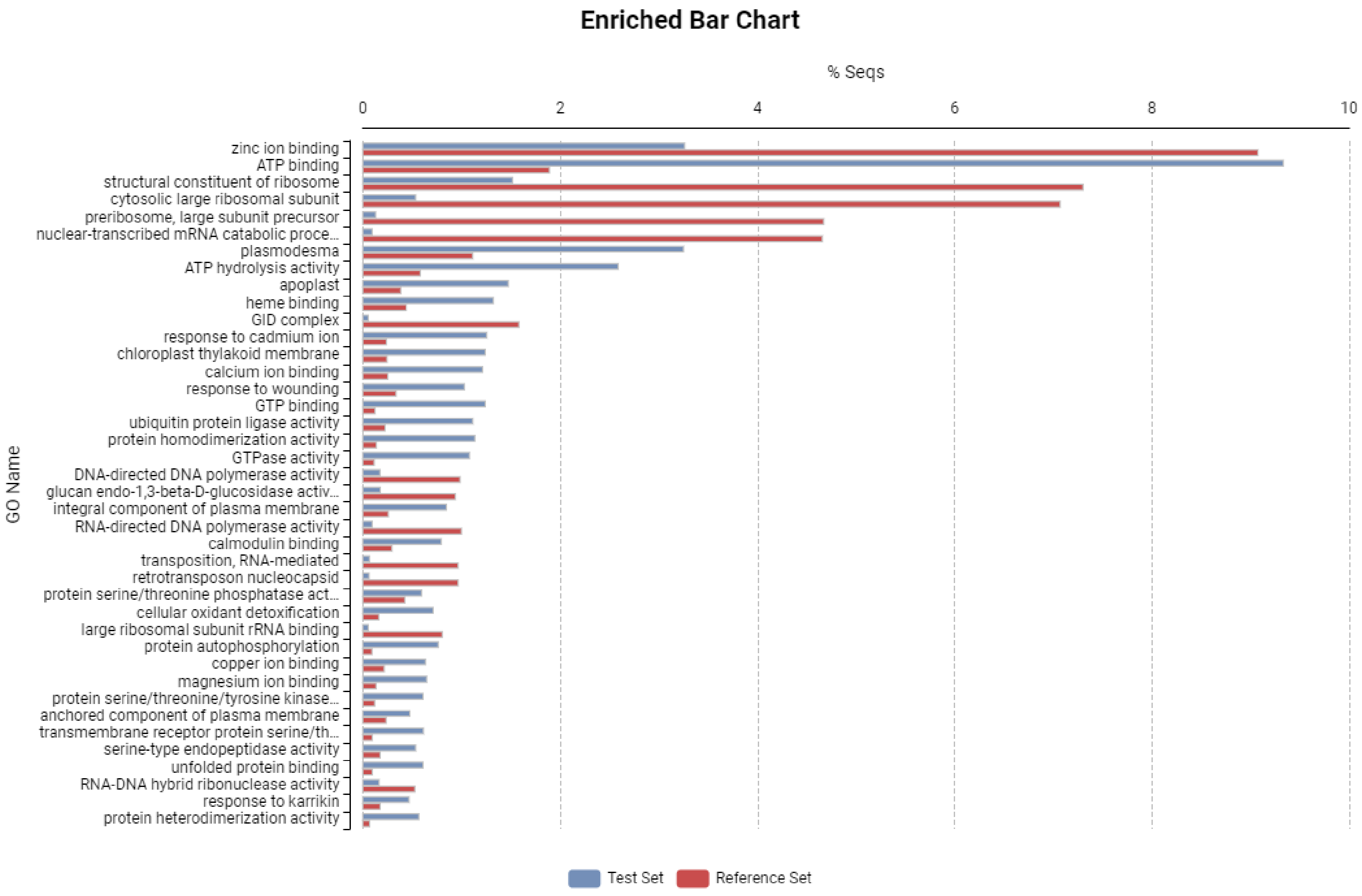

Figure S10. Fisher's Exact Test comparison of EG11 gene models. Comparison of gene ontology terms in Class N1, N2, N3 and N4 gene models with expression evidence (Test Set) to the Reference Set of gene models without any expression evidence (Class N5 and N6) showed that the Reference Set was enriched with house-keeping genes.

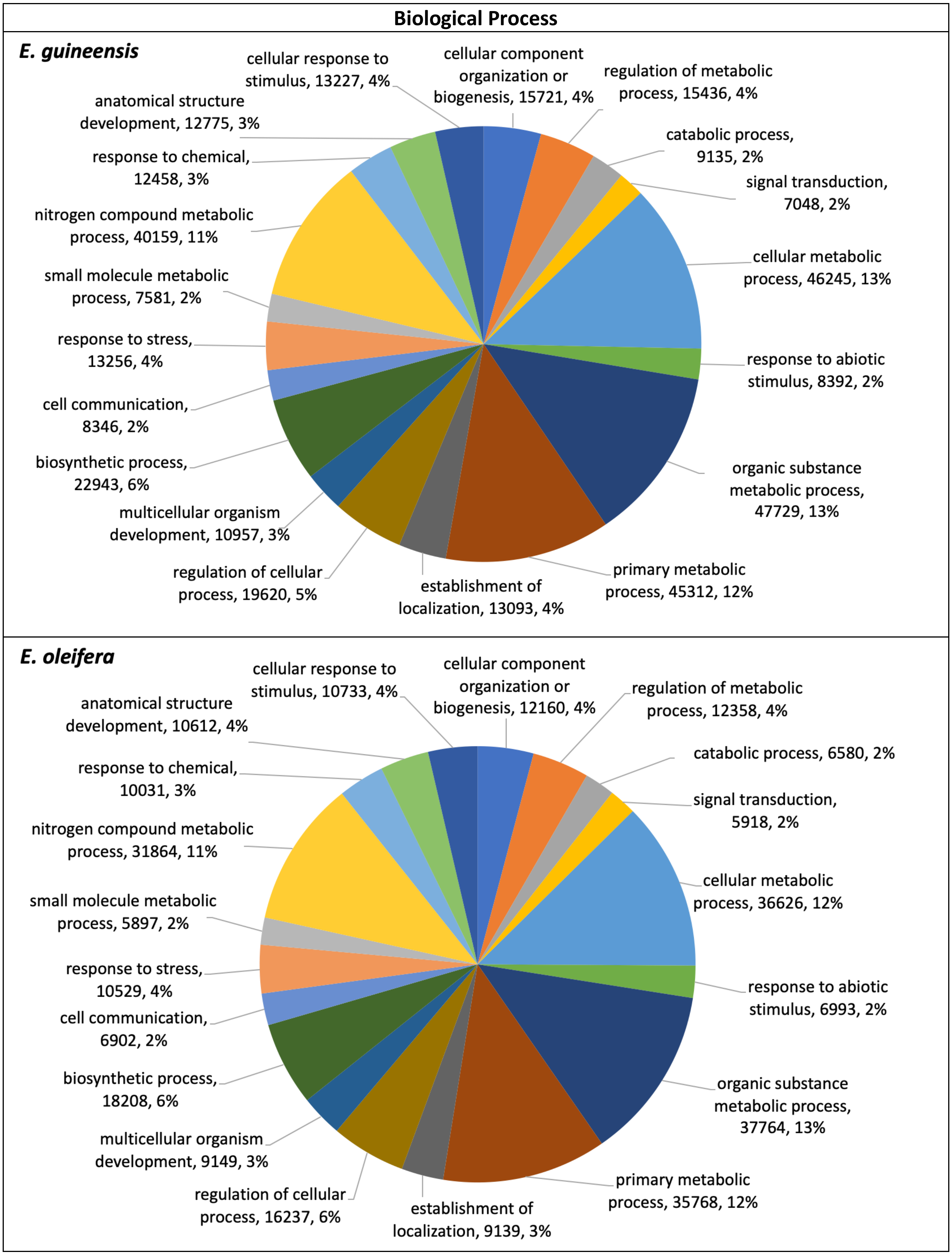

Figure S11. Gene ontology classifications of the oil palm selected gene models.

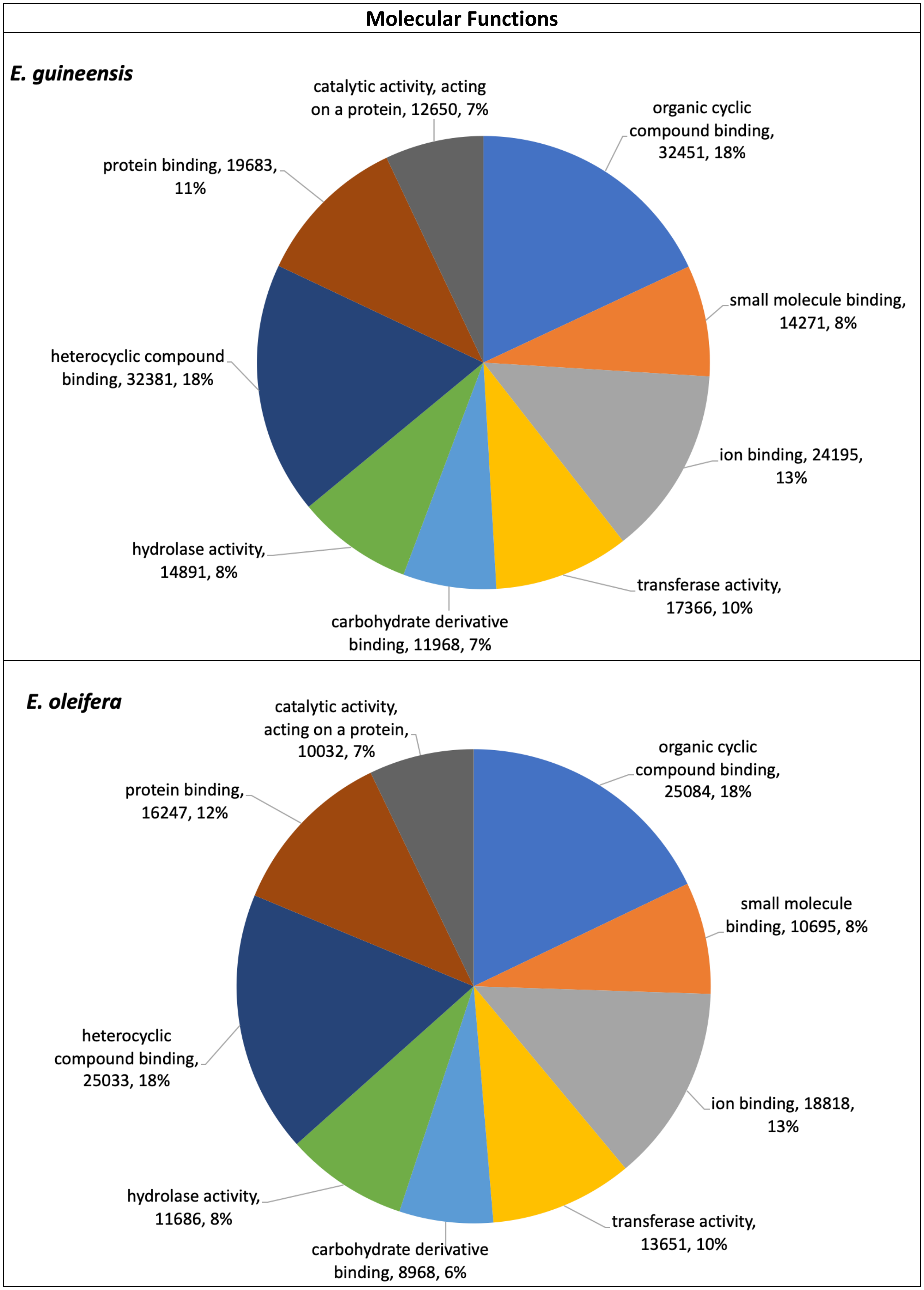

Figure S11 continued. Gene ontology classifications of the oil palm selected gene models

Cellular Component

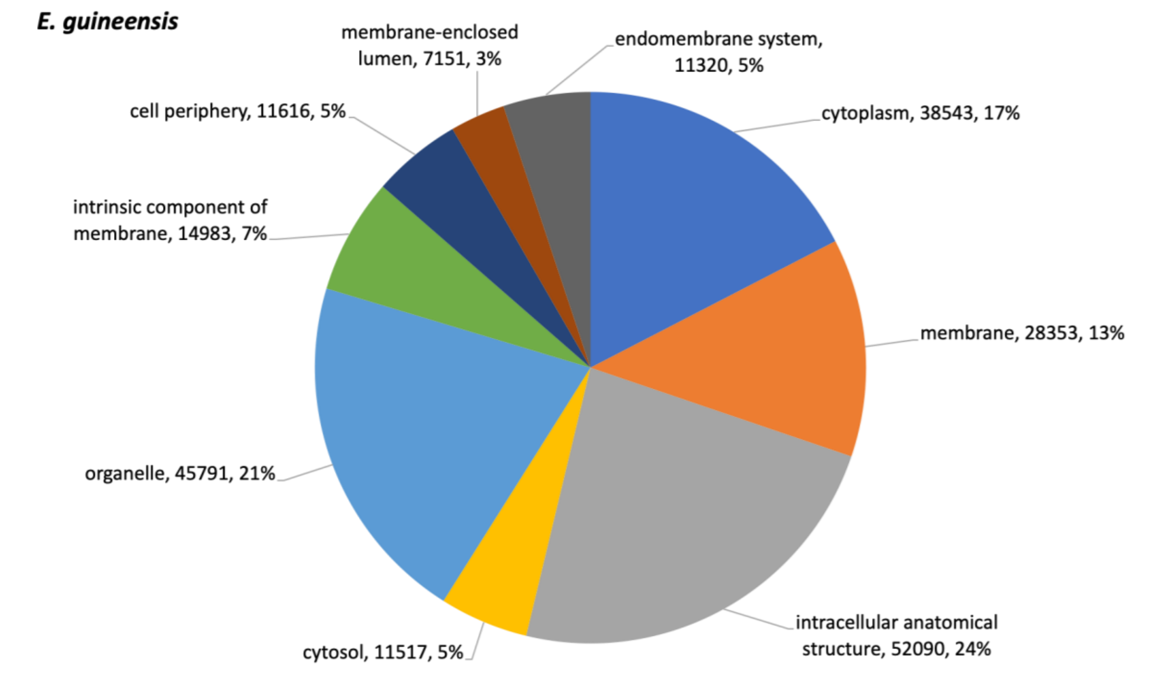

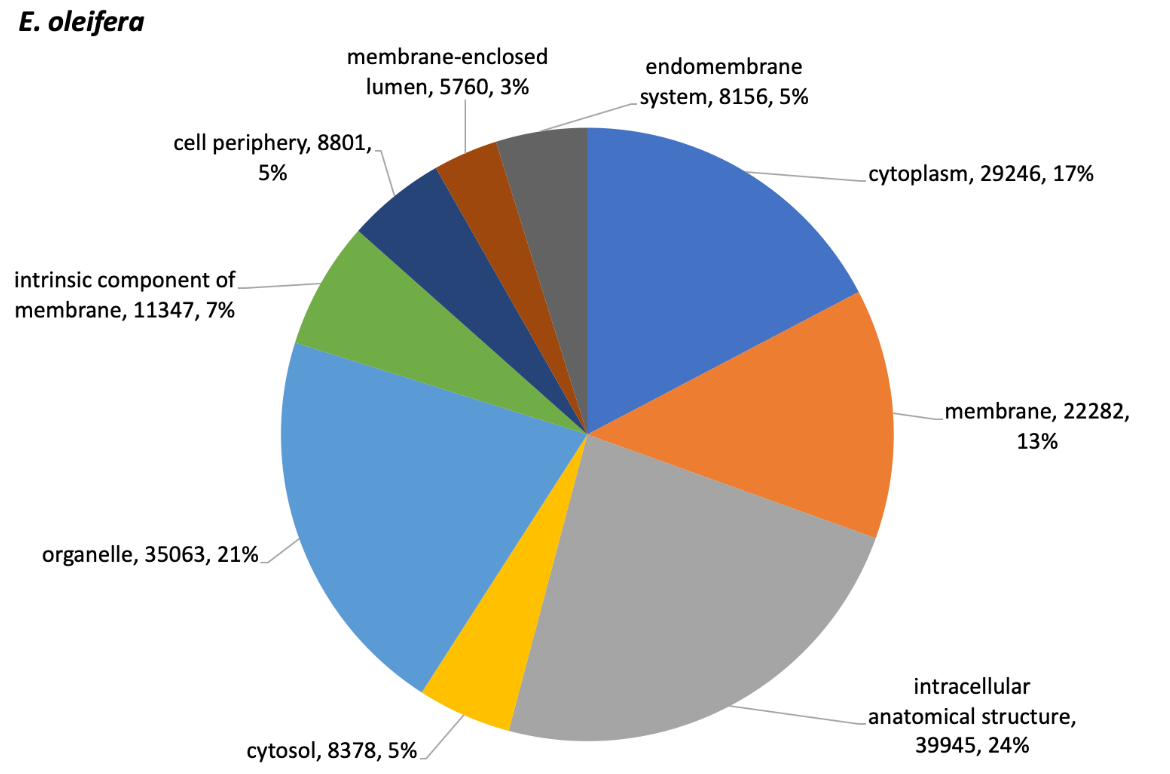

Figure S11 continued. Gene ontology classifications of the oil palm selected gene models

a. b.

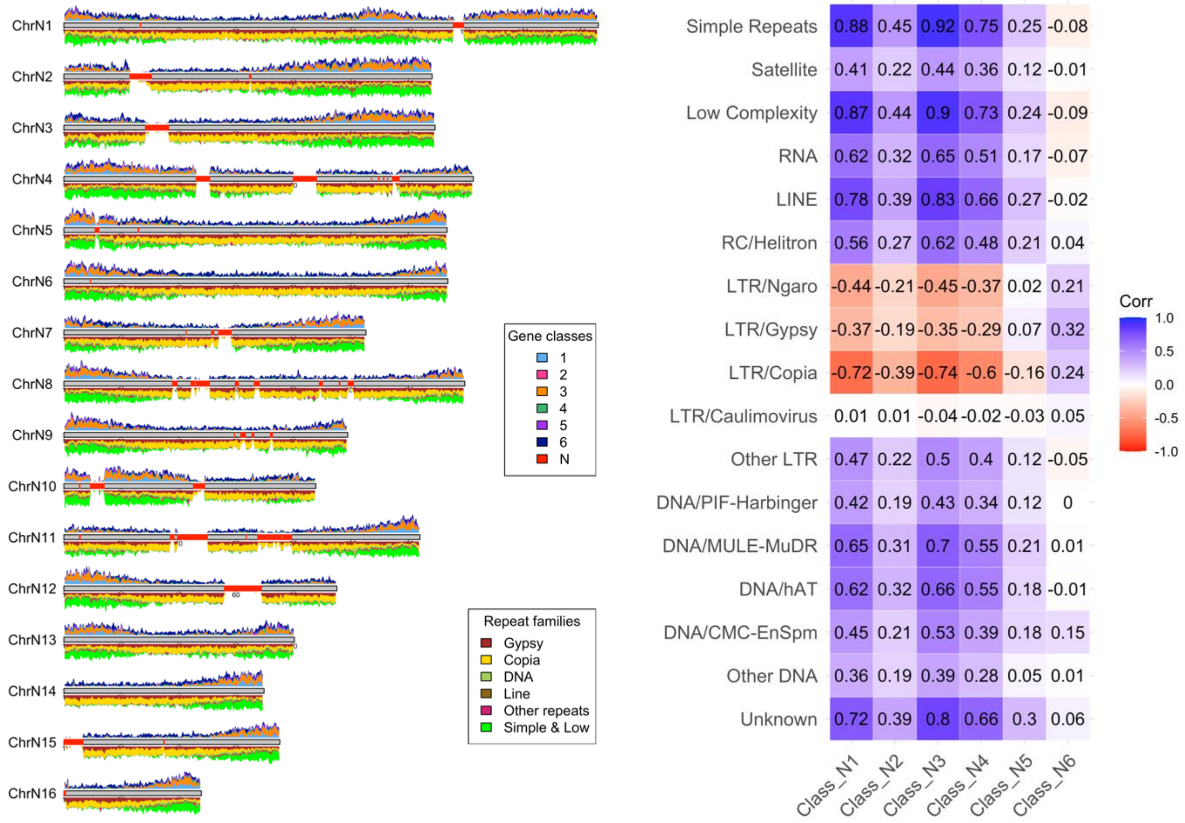

Figure S12. Oil palm genes and repeat elements. Genomic context of **a.** *E. guineensis* (EG) and **c.** *E. oleifera* (EO) genes and repeat elements across the 16 chromosomes. The gene models are classified into 6 classes according to the availability of supporting CAGE, RNAseq and Blast data. Class N1: CAGE, RNAseq and BLAST; Class N2: CAGE, and RNAseq or BLAST; Class N3: RNAseq and BLAST; Class N4: CAGE or RNAseq; Class N5: BLAST; Class N6: No additional support data. Repeat elements were identified using Repeatmodeler2. In general, **b.** Class N1-N4 of EG and **d.** Class N1-N6 of EO gene models are negatively correlated with LTR/Gypsy, LTR/Copia and LTR/Nagaro, but positively correlated with the other repeat classes. EG Class N6 is the only class that have positive correlation LTR/Gypsy, LTR/Copia and LTR/Nagaro and low levels of correlations with all repeat classes. All classes are not correlated with LTR/coulimovirus.

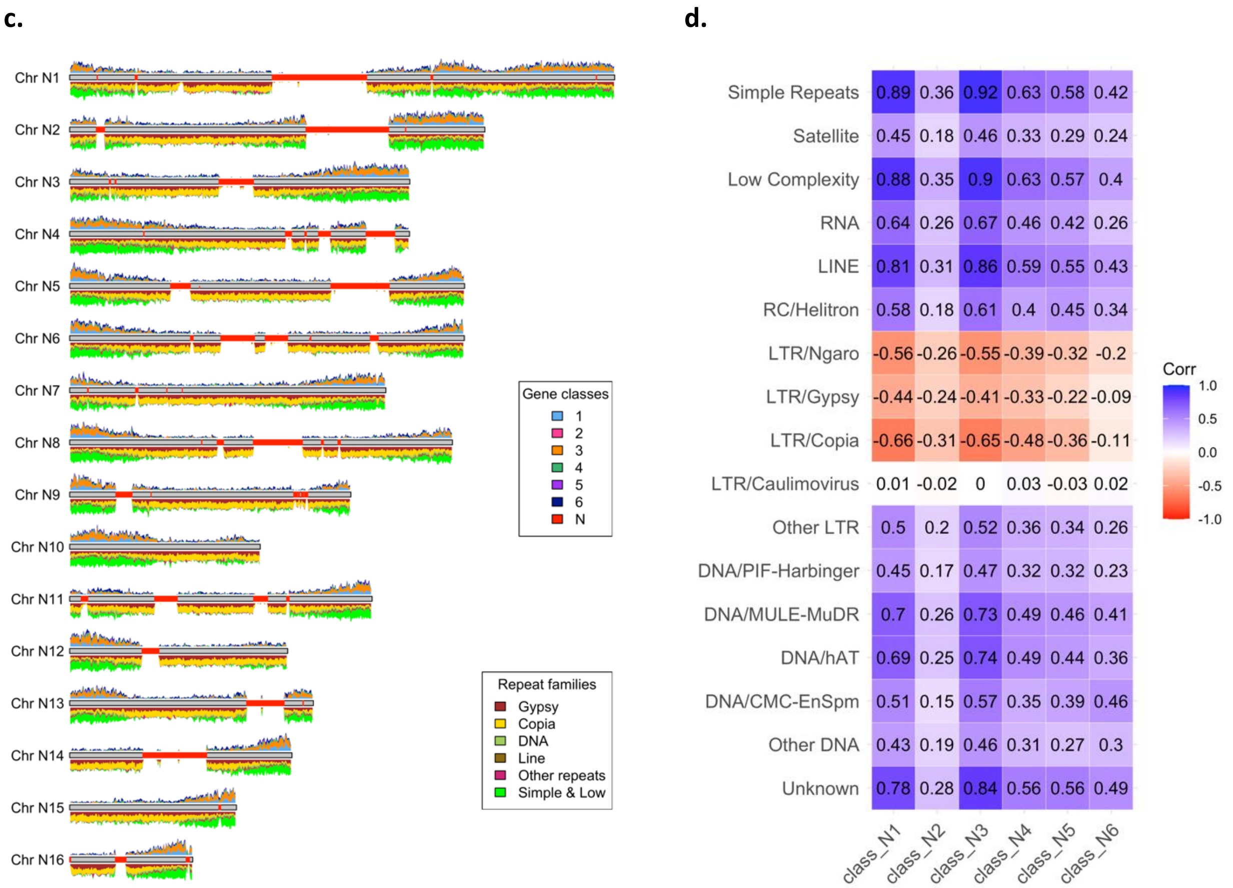

Figure S12 continued. Oil palm genes and repeat elements. Genomic context of **a.** *Elaeis guineensis* (EG) and **b.** *Elaeis oleifera* (EO) genes and repeat elements across the 16 chromosomes. The gene models are classified into 6 classes according to the availability of supporting CAGE, RNAseq and Blast data. Class N1: CAGE, RNAseq and BLAST; Class N2: CAGE, and RNAseq or BLAST; Class N3: RNAseq and BLAST; Class N4: CAGE or RNAseq; Class N5: BLAST; Class N6: No additional support data. Repeat elements were identified using Repeatmodeler2. In general, **b.** Class N1-N4 of EG and **d.** Class N1-N6 of EO gene models are negatively correlated with LTR/Gypsy, LTR/Copia and LTR/Nagaro, but positively correlated with the other repeat classes. EG Class N6 is the only class that have positive correlation LTR/Gypsy, LTR/Copia and LTR/Nagaro and low levels of correlations with all repeat classes. All classes are not correlated with LTR/coulimovirus.

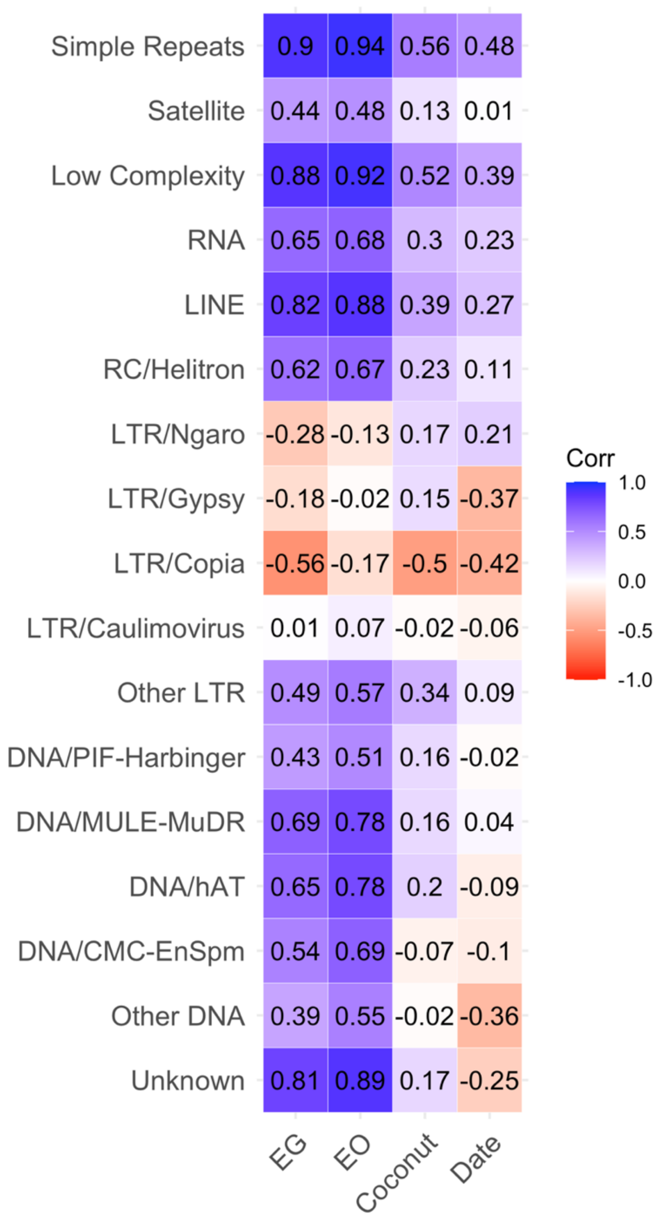

Figure S13. Correlation analysis of EG/EO/Coconut/Date Palm repeat structures and gene models.

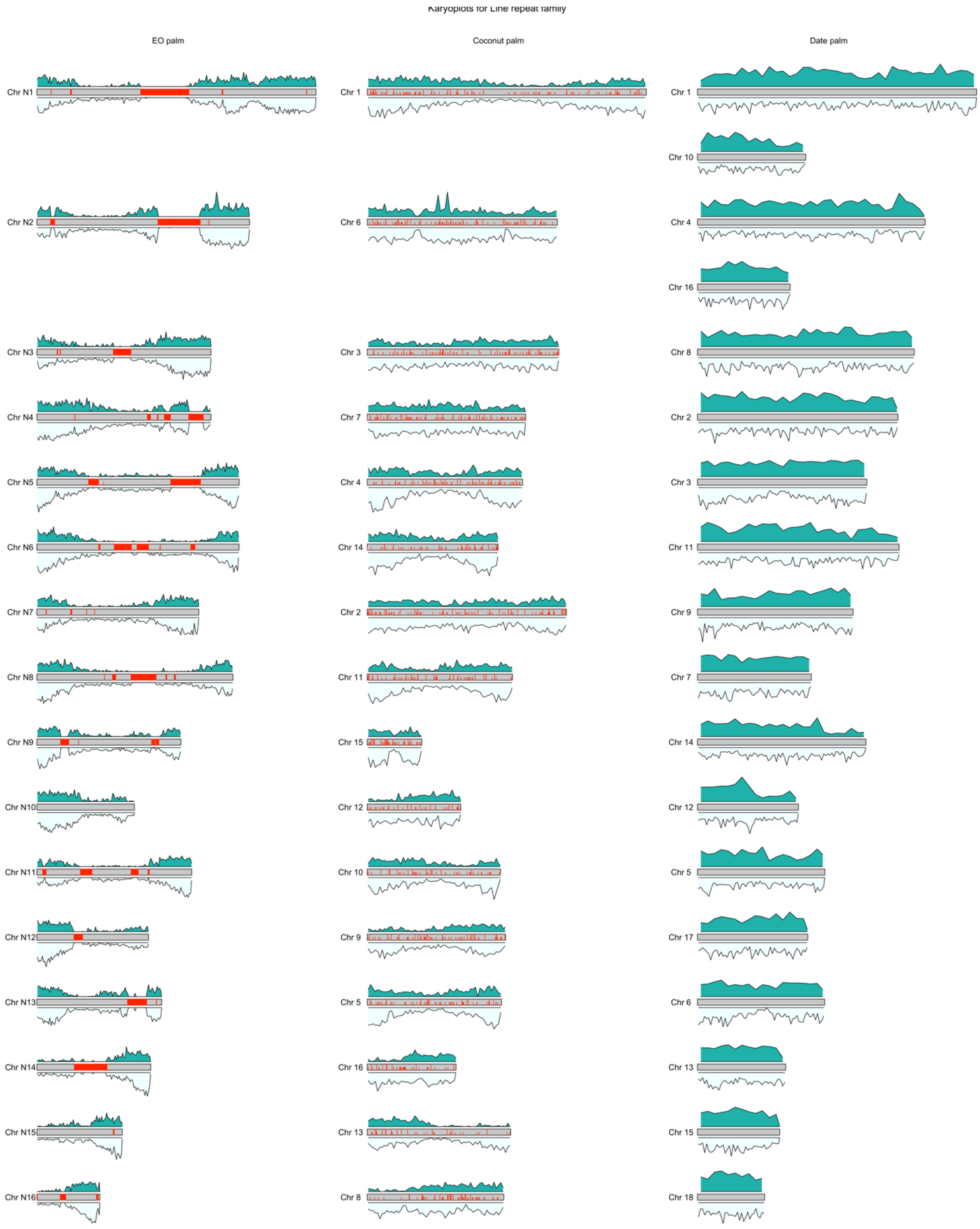

Figure S14. ***E. oleifera, C. nucifera*** **and** ***P. dactylifera*** **LINE karyoplots.** LINE and gene densities are plotted in turquoise and light blue, respectively. Red lines are gaps.

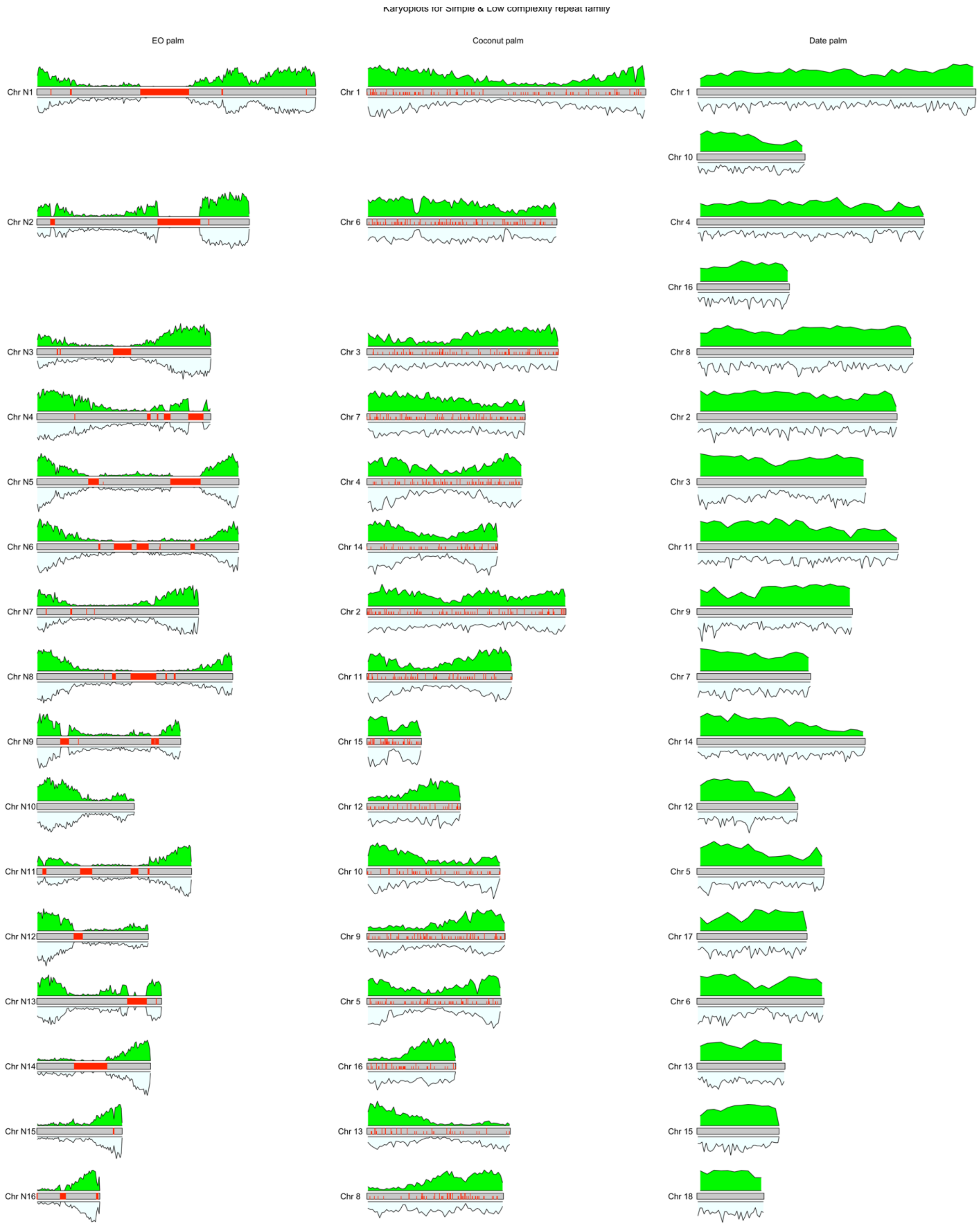

Figure S15. ***E. oleifera, C. nucifera*** **and** ***P. dactylifera*** **simple and low complexity repeat karyoplots.** Simple and low complexity repeats, and gene densities are plotted in green and light blue, respectively. Red lines are gaps.

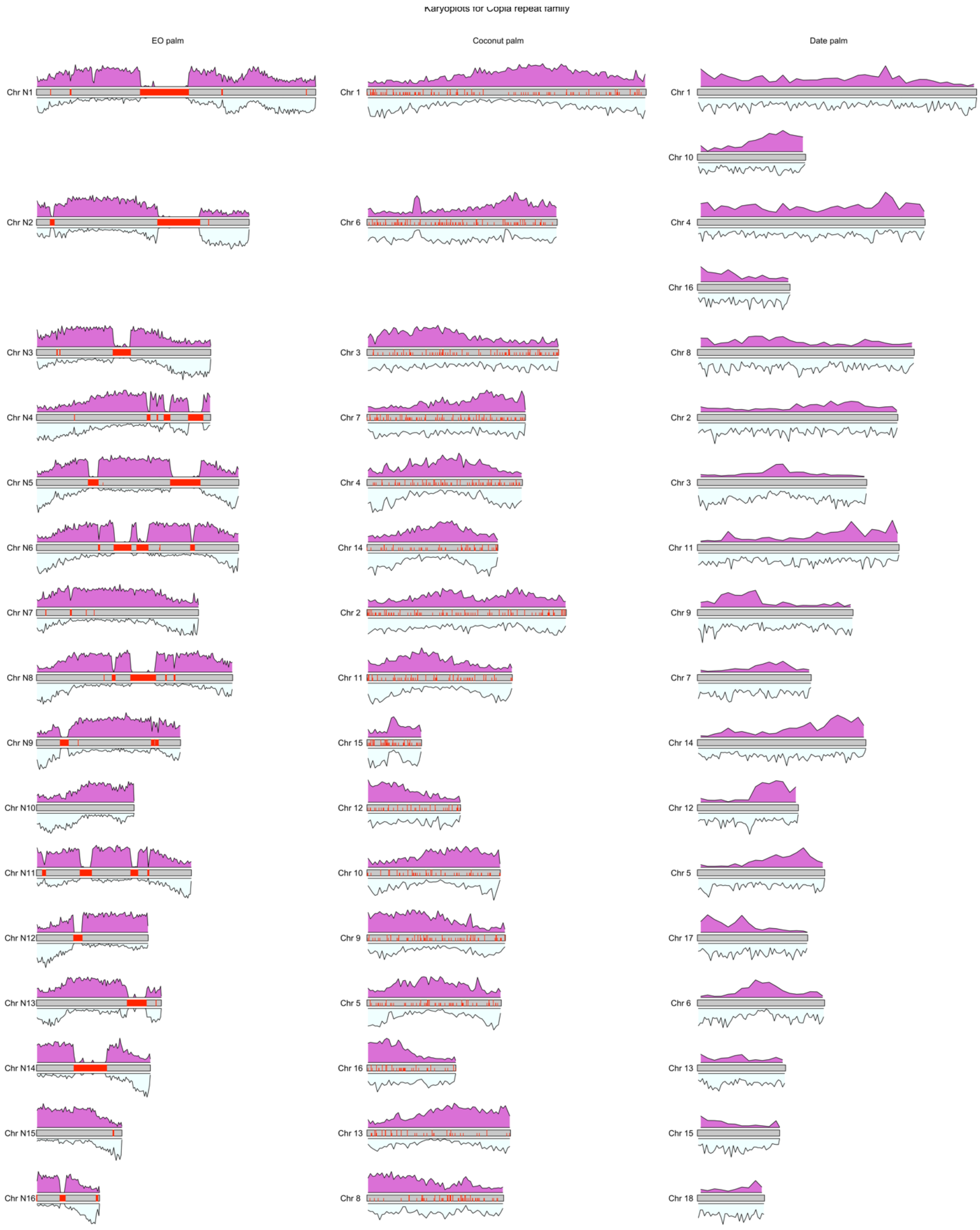

Figure S16. ***E. oleifera, C. nucifera*** **and** ***P. dactylifera*** **copia karyoplots.** Copia and gene densities are plotted in purple and light blue, respectively. Red lines are gaps.

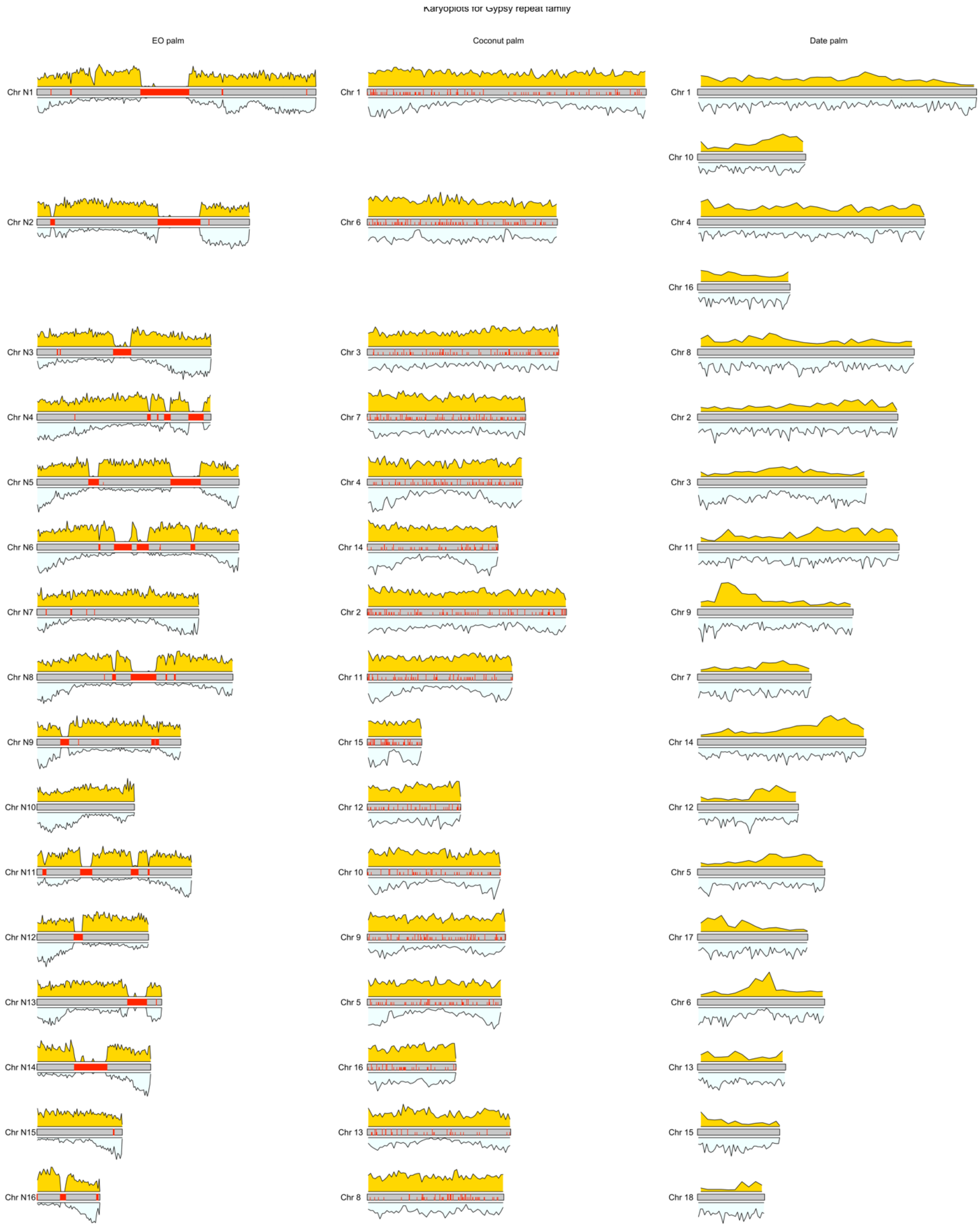

Figure S17. ***E. oleifera, C. nucifera*** **and** ***P. dactylifera*** **gypsy karyoplots.** Gypsy and gene densities are plotted in yellow and light blue, respectively. Red lines are gaps.

Table S1. Genome assembly statistics relative to previously published *E. guineensis* and *E. oleifera* assemblies.

	***Elaeis guineensis* (EG)**					***Elaeis oleifera* (EO)**			
	**Published (Singh, Ong-Abdullah, *et al.* 2013)**		**Published (Wang *et al.* 2023)**	**Latest Assemblies**		**Published (Singh et al., 2013)**		**Latest Assemblies**	
	**Scaffolds**	**Chromosome**	**Chromosome**	**Scaffolds**	**Chromosome**	**Scaffolds**	**Chromosome**	**Scaffolds**	**Chromosome**
**Build**	**P5**	**EG5**	**Egu.V3**	**P11d2**	**EG11**	**O8**		**O12e2**	**EO12.1**
No. of Scaffolds	40,360	40,072	932	73	39	26,769		43	26
Total bases	1,535,150,282	1,535,179,082	1,701,312,507	1,867,264,587	1,867,267,987	1,402,851,853		2,044,354,600	2,042,916,827
GC content	37.21%	37.21%	38.7%	38.54%	38.54%	37.99%		39.33%	39.33%
Gap content	31.14%	31.14%	0.03%	7.95%	7.95%	25.98%		14.43%	14.37%
Maximum length	22,100,610	68,435,666	160,148,325	122,693,502	186,201,133	4,597,586		217,745,620	226,035,659
Minimum length	1,992	1,992	511	93,154	93,154	1,966		89,593	89,593
Mean length	38,036	38,311	1,825,442	25,578,967	47,878,666	52,406	**NA**	47,543,130	78,573,724
N50 length	1,045,414	1,268,079	111,579,804	53,861,278	128,314,321	333,109		123,833,504	141,087,874
N90 length	32,040	32,090	37,783,746	17,063,465	69,731,926	35,717		37,011,007	79,040,982
Gene Models	34,802		33,447	46,697		NA		38,658	
**Total no. bases in 16 Chromosomes**		**657,940,036**	**1,558,018,416**		**1,777,716,855**				**2,022,763,378**
**% Gap in 16 Chromosomes**		**22.90%**	**0.03%**		**5.63%**				**13.76%**

Table S2. EG11 and EO12.1 transcript statistics

	EG11	EO12.1
**No. of Models**		
**Transcripts (Gene Models)**	105,560 (46,697)	80,520 (38,658)
**Long non-coding RNA (lncRNA)**	28,704	25,130
**Gene Models Quality Assessment (BUSCO5 Liliopsida Profile) (Transcript)**		
**Complete BUSCOs**	2567 (79.3%)	2874 (88.8%)
**Complete and single copy BUSCOs**	1036 (32.0%)	1168 (36.1%)
**Complete and duplicated BUSCOs**	1531 (47.3%)	1706 (52.7%)
**Fragmented BUSCOs**	282 (8.7%)	152 (4.7%)
**Missing BUSCOs**	387 (12.0%)	210 (6.5%)
**Total BUSCO groups searched**	3236	3236

Table S3. Summary of pseudochromosome naming between EG5 and EG11/EO12.1

Chromosome	EG5	EG11/EO12.1
Chromosome 1	Chr 2	Chr N1
Chromosome 2	Chr 1	Chr N2
Chromosome 3	Chr 3	Chr N3
Chromosome 4	Chr 4	Chr N4
Chromosome 5	Chr 5	Chr N5
Chromosome 6	Chr 6	Chr N6
Chromosome 7	Chr 7	Chr N7
Chromosome 8	Chr 8	Chr N8
Chromosome 9	Chr 9	Chr N9
Chromosome 10	Chr 10	Chr N10
Chromosome 11	Chr 11	Chr N11
Chromosome 12	Chr 12	Chr N12
Chromosome 13	Chr 13	Chr N13
Chromosome 14	Chr 14	Chr N14
Chromosome 15	Chr 15	Chr N15
Chromosome 16	Chr 16	Chr N16

Table S4. Summary of EG11 guided edits to EO12.1

	Prior to Edit			After Edit				Evidence		
o12e Scaffold	Start	End	Orientation	Pseudochr	Start	End	Change	o12c (HiC)	(optical map)	BACs
o12e_sc0008	1	17,041,178	+	chr N14	1	17,041,178		+	+	0
o12e_sc0008	52,221,916	92,209,035	+	chr N14	17,041,279	57,028,397	edited	-	+	0
o12e_sc0008	46,070,589	52,221,816	+	chr N14	57,028,498	63,179,724	edited	-	-	0
o12e_sc0008	17,041,278	46,070,490	-	chr N14	63,179,825	92,209,035	edited	+	+	0
o12e_sc0001	1	51,750,747	+	chr N13	1	51,750,747				
o12e_sc0025	8,800,677	14,491,423	-	chr N13	51,750,848	57,441,593	edited	-	+	+
o12e_sc0001	51,750,847	58,920,514	-	chr N13	57,441,694	64,611,360				
o12e_sc0025	1	8,800,577	-	chr N13	64,611,461	73,412,037	edited	+		
o12e_sc0001	64,967,430	99,664,872	+	chr N13	73,412,138	101,086,741				+
o12e_sc0006	1	7,738,746	+	chr N10	1	7,738,746				+
o12e_sc0016	28,934,782	47,628,987	-	chr N10	7,738,847	26,433,051	edited	0	0	+
o12e_sc0016	19,832,320	28,934,682	+	chr N10	26,433,152	35,535,513	edited	0	0	+
o12e_sc0016	13,337,988	19,832,220	+	chr N10	35,535,614	42,029,875	edited	0	0	+
o12e_sc0019	10,458,032	33,750,936	-	chr N10	42,029,976	65,322,879	edited	+	0	+
o12e_sc0019	1	10,457,932	+	chr N10	65,322,980	75,780,911		0	0	+
o12e_sc0019	33,751,036	37,011,007	+	chr N10	75,781,012	79,040,982		0	0	+
o12e_sc0011	5,803,615	19,087,687	-	chr N9	1	13,284,072	edited	-	+	0
o12e_sc0011	1	5,803,515	+	chr N9	13,284,173	19,087,687				0
o12e_sc0018	1	44,080,037	-	chr N9	19,087,788	63,167,824	edited	-	-	0
o12e_sc0011	19,087,787	72,557,537	-	chr N9	63,167,925	116,637,674				0
o12e_sc0002	42,941,262	111,590,868	+	chr N8	1	68,649,606	edited	0	0	
o12e_sc0002	1	7,494,346	-	chr N8	68,649,707	76,144,052	edited	0	0	
o12e_sc0002	111,590,967	158,768,208	-	chr N8	76,144,153	123,321,392	edited	0	0	
o12e_sc0002	7,494,446	35,224,330	+	chr N8	123,321,493	151,051,375	edited	0	0	
o12e_sc0002	35,224,428	42,941,162	-	chr N8	151,051,476	158,768,208	edited	0	0	
o12e_sc0004	87,211,796	146,119,212	-	chr N6	1	58,907,416	edited	0	0	+
o12e_sc0030	1	3,697,557	+	chr N6	58,907,517	62,605,073		0	0	+
o12e_sc0004	57,852,354	87,211,796	-	chr N6	62,605,174	90,530,588	edited	0	0	
o12e_sc0027	1	9,575,253	-	chr N6	90,530,689	100,105,941	edited	0	0	
o12e_sc0028	1	5,740,285	-	chr N6	100,106,042	105,846,326	edited	0	0	
o12e_sc0004	1	57,852,354	-	chr N6	105,846,427	163,698,780	edited	0	0	
o12e_sc0010	55,487,337	84,821,544	+	chr N5	1	29,334,207	edited	0	0	0
o12e_sc0026	1	12,326,088	+	chr N5	29,334,308	41,660,395	edited	0	0	0
o12e_sc0014	1	61,174,386	+	chr N5	41,660,496	102,834,881	edited	0	0	0
o12e_sc0029	1	5,472,381	+	chr N5	102,834,982	108,307,362	edited	0	0	0
o12e_sc0010	1	55,487,237	+	chr N5	108,307,463	163,794,699	edited	0	0	0
o12e_sc0007	1	108,221,440	+	chr N4	-	108,221,440				
o12e_sc0024	1	14,707,336	-	chr N4	108,221,541	122,928,876	edited	+	-	0
o12e_sc0007	108,222,325	123,833,504	+	chr N4	122,928,977	138,540,155				0
o12e_sc0034	1	2,426,273	+	chr N4	138,540,256	140,966,528				0
o12e_sc0006	7,738,847	139,759,893	-	chr N3	1	132,021,047	edited	+	+	+
o12e_sc0016	47,629,087	56,695,814	+	chr N3	132,021,148	141,087,874	edited	+	+	+
o12e_sc0003	121,717,353	147,278,685	-	chr N2	1	25,561,333			+	+
o12e_sc0003	74,185,647	121,717,252	+	chr N2	25,561,434	73,093,039	edited	-	+	+
o12e_sc0022	1	24,929,063	-	chr N2	73,093,140	98,022,202	edited		+	0
o12e_sc0003	1	74,180,487	-	chr N2	98,022,303	172,202,789				
o12e_sc0015	1	57,851,820	-	chr N1	1	57,851,820	edited	+	-	-
o12e_sc0021	1	26,081,949	+	chr N1	57,851,921	83,933,869				
o12e_sc0005	1	142,101,690	+	chr N1	83,933,970	226,035,659	edited	+	+	-
						Total Edits:	33			

Prior to Edit columns provide the o12e assembly coordinates and orientation (+/-) for scaffolds prior to EG11-guided edits to produce the final EO12.1 assembly. After Edit columns provide EO12.1 pseudochromosome number and coordinates. For each pseudochromosome involved, "edited" indicates the scaffold number that was edited to produce the final EO12.1 assembly. Evidence columns summarize the supporting evidence for each edit based on HiC proximity ligation data alone (O12c (HiC)), optical mapping data alone (optical map) and pooled BAC sequences (BACs). +, evidence supporting the edit. -, evidence not supporting the edit, 0, no evidence.

Table S5. Candidate indel structural variants with PCR support evidence

	EG11				EO12.1			
event_name	chr	start	end	gene_annotation_in_region	chr	start	end	gene_annotation_in_region
event_1	chr N8	60,790,279	60,811,996	uncharacterized protein LOC105036010 [Elaeis guineensis]				
				ribonuclease TUDOR 1-like isoform X1 [Phoenix dactylifera]	chr N8	58,925,177	58,945,197	Ribonuclease TUDOR 1 (AtTudor1) (TUDOR-SN protein 1) (EC 3.1.-.-)
event_2	chr N8	2,791,511	2,819,488	protein GLUTAMINE DUMPER 3-like protein [Carex littledalei]	chr N8	2,604,938	2,624,949	Protein GLUTAMINE DUMPER 3 (Protein LESS SUSCEPTIBLE TO BSCTV 1) (Protein LBS1)
				retrotransposon protein%2c putative%2c Ty1-copia subclass [Oryza sativa Japonica Group]				
event_3	chr N3	5,173,013	5,208,180	T-complex protein 1 subunit theta [Elaeis guineensis]	chr N3	4,391,708	4,413,743	Photosynthetic NDH subunit of subcomplex B 2, chloroplastic (Protein PnsB2)
event_4	chr N9	33,377,253	33,407,741	DEAD-box ATP-dependent RNA helicase 58%2c chloroplastic isoform X1 [Elaeis guineensis]	window not found in EO			
event_5	chr N15	17,922,085	17,947,547	no_annotation	chr N15	11,738,296	11,758,308	Vacuole membrane protein KMS1 (Protein KILLING ME SLOWLY 1)
								Vacuole membrane protein KMS2 (Protein KILLING ME SLOWLY 2)
event_6	chr N14	60,296,401	60,321,381	acetate/butyrate--CoA ligase AAE7%2c peroxisomal [Elaeis guineensis]	chr N14	82,965,391	82,985,401	Acetate--CoA ligase CCL3 (HICCL3) (EC 6.2.1.1)
event_7	chr N1	41,368,371	41,427,067	uncharacterized protein LOC105032977 [Elaeis guineensis]	window not found in EO			
event_8	chr N1	67,783,025	67,821,117	hypothetical protein EE612_037101 [Oryza sativa]	chr N1	75,834,043	75,854,048	Probable inactive receptor kinase At5g10020
event_9	chr N11	11,663,314	11,690,876	aconitate hydratase%2c cytoplasmic [Elaeis guineensis]	chr N11	12,887,899	12,907,926	Aconitate hydratase, cytoplasmic (Aconitase) (EC 4.2.1.3) (Citrate hydro-lyase)
				dnaJ homolog subfamily C member 17 isoform X1 [Elaeis guineensis]				
event_10	chr N3	128,022,691	128,064,721	uncharacterized protein LOC114913679 [Elaeis guineensis]	chr N3	139,841,434	139,861,450	Protein OVEREXPRESSOR OF CATIONIC PEROXIDASE 3
				zinc finger CCCH domain-containing protein 34 [Elaeis guineensis]				Zinc finger CCCH domain-containing protein 34 (OsC3H34)
event_11	chr N3	127,838,103	127,890,070	uncharacterized protein LOC114913679 [Elaeis guineensis]	window not found in EO			
	chr N3	127,838,103	127,890,070	monothiol glutaredoxin-S4%2c mitochondrial isoform X1 [Phoenix dactylifera]				
	chr N3	127,838,103	127,890,070	CRC domain-containing protein TSO1 isoform X2 [Elaeis guineensis]				
	chr N3	127,838,103	127,890,070	CRC domain-containing protein TSO1 isoform X3 [Elaeis guineensis]				
event_12	chr N2	71,816,440	71,845,452	no_annotation	chr N2	83,848,286	83,868,297	No protein found
event_13	chr N10	14,103,836	14,139,506	uncharacterized protein LOC114913679 [Elaeis guineensis]	window not found in EO			
	chr N10	14,103,836	14,139,506	protein Brevis radix-like 1 isoform X1 [Elaeis guineensis]				
	chr N10	14,103,836	14,139,506	uncharacterized protein LOC105053113 isoform X1 [Elaeis guineensis]				
event_14	chr N1	17,806,704	17,844,315	no_annotation	chr N1	17,138,289	17,158,310	No protein found
event_15	chr N9	92,196,187	92,221,760	no_annotation	chr N9	110,216,911	110,236,926	Chaperone protein DnaJ
event_16	chr N4	41,252,556	41,282,953	probable phospholipid hydroperoxide glutathione peroxidase [Elaeis guineensis]	chr N4	36,803,508	36,823,518	AT-hook motif nuclear-localized protein 11
				PREDICTED: AT-hook motif nuclear-localized protein 9-like isoform X2 [Musa acuminata subsp. malaccensis]				FT-interacting protein 3 (Multiple C2 domain and transmembrane region protein 3)
				LOW QUALITY PROTEIN: AT-hook motif nuclear-localized protein 9-like [Elaeis guineensis]				Probable phospholipid hydroperoxide glutathione peroxidase (PHGPx) (EC 1.11.1.12)
				FT-interacting protein 3 [Elaeis guineensis]				
event_17	chr N2	54,773,842	54,831,479	no_annotation	window not found in EO			
event_18	chr N13	79,454,051	79,487,657	uncharacterized protein LOC109505530 [Elaeis guineensis]	chr N13	98,803,994	98,824,019	ACT domain-containing protein ACR1 (Protein ACT DOMAIN REPEATS 1)
				uncharacterized protein LOC109505143 [Elaeis guineensis]				
				PREDICTED: uncharacterized protein LOC103980200 isoform X1 [Musa acuminata subsp. malaccensis]				

PacBio subreads generated for EO12.1 were aligned to both EO12.1 and EG11 and used to identify candidate deletions in EO12.1 relative to EG11 (Methods). PCR support was obtained for 18 of 18 predicted deletions. Because deletions may impact neighboring genes even if the coding region of the gene is not deleted, sequences including the deleted region (in EG11) plus 10 Kb of sequence flanking the region were annotated based on mapping of sequences to each assembly, and annotations within these sequence windows are reported. Multiple entries for a given event/assembly indicate more than one annotation within the sequence window. For five events, the sequence window was not identified in EO12.1, indicating larger deletions.

Table S5 continued. Candidate duplication structural variants with PCR support evidence

	EG11				EO12.1			
event_name	chr	start	end	gene_annotation_in_region	chr	start	end	gene_annotation_in_region
event_1	chr N14	62,751,533	62,771,962	hypothetical protein BHM03_00021717 [Ensete ventricosum]				
				ABC transporter B family member 25%2c mitochondrial [Phoenix dactylifera]	chr N14	85,550,214	85,571,085	ABC transporter B family member 25, mitochondrial (Protein STARIK 1)
event_2	chr N2	69,971,601	69,992,826	kinesin-like protein KIN-12F isoform X2 [Elaeis guineensis]	chr N2	81,783,879	81,806,355	No annotation
event_3	chr N2	94,749,238	94,769,545	probable beta-1%2c3-galactosyltransferase 2 [Elaeis guineensis]	chr N2	141,185,506	141,206,170	Probable beta-1,3-galactosyltransferase 2 (EC 2.4.1.-)
				uncharacterized protein LOC108511668 [Phoenix dactylifera]				
event_4	chr N11	113,923,432	113,943,752	tetratricopeptide repeat protein 27 homolog [Elaeis guineensis]	chr N11	115,563,748	115,584,387	Electron transfer flavoprotein-ubiquinone oxidoreductase, mitochondrial (ETF-QO)
					chr N11	115,563,748	115,584,387	Heat shock protein STI1

PacBio subreads generated for EO12.1 were aligned to both EO12.1 and EG11 and used to identify candidate tandem duplications in EO12.1 relative to EG11 (Methods). PCR support was obtained for 4 of 7 predicted duplications. Because duplications may impact neighboring genes even if the coding region of the gene is not duplicated, sequences including the duplicated region (in EG11) plus 10 Kb of sequence flanking the region were annotated based on mapping of sequences to each assembly, and annotations within these sequence windows are reported. Multiple entries for a given event/assembly indicate more than one annotation within the sequence window.

Table S5 continued. Candidate translocation structural variants with PCR support evidence

	EG11				EO12.1			
event_name	chr	start	end	gene_annotation_in_region	chr	start	end	gene_annotation_in_region
event_1	chr N13	3,136,629	3,147,129	alternative NAD(P)H-ubiquinone oxidoreductase C1%2c chloroplastic/mitochondrial isoform X1 [Elaeis guineensis]	chr N13	3,175,962	3,196,977	Alternative NAD(P)H-ubiquinone oxidoreductase C1, chloroplastic/mitochondrial (EC 1.6.5.9)
	chr N9	6,041,072	6,051,572	uncharacterized protein LOC103703199 [Phoenix dactylifera]				
event_2	chr N9	7,576,972	7,587,472	potassium channel AKT2 isoform X1 [Elaeis guineensis]	chr N9	5,942,366	5,963,411	Short-chain dehydrogenase TIC 32, chloroplastic (EC 1.1.1.-) (Translocon at the inner envelope membrane of chloroplasts 32) (AtTIC32)
					chr N9	5,942,366	5,963,411	Zinc-finger homeodomain protein 2 (AtZHD2) (Homeobox protein 22) (AtHB-22) (Protein MATERNAL EFFECT EMBRYO ARREST 68)
	chr N14	31,097,636	31,108,136	LOW QUALITY PROTEIN: zinc-finger homeodomain protein 2-like [Elaeis guineensis]				
event_3	chr N4	9,446,170	9,456,670	uncharacterized protein LOC105044011 isoform X1 [Elaeis guineensis]	chr N4	7,643,005	7,664,014	Translation initiation factor IF3-1, mitochondrial (AtIF3-1) (AtINFC-1)
					chr N4	7,643,005	7,664,014	Alpha-glucosidase 2 (EC 3.2.1.20) (Alpha-glucosidase II)
	chr N9	23,089,971	23,100,471	no_annotation				
event_4	chr N6	120,019,841	120,030,341	protein FAR1-RELATED SEQUENCE 5 isoform X2 [Elaeis guineensis]	chr N6	149,909,060	149,930,070	LOB domain-containing protein 4 (ASYMMETRIC LEAVES 2-like protein 6) (AS2-like protein 6)
	chr N1	7,187,638	7,198,138	protein FAR1-RELATED SEQUENCE 5 isoform X1 [Elaeis guineensis]				
event_5	chr N6	12,513,154	12,523,654	retrotransposon protein%2c putative%2c Ty1-copia subclass [Oryza sativa Japonica Group]	chr N6	10,923,900	10,944,905	Cytochrome P450 CYP72A219 (EC 1.14.-.-) (Cytochrome P450 CYP72A129)
	chr N15	51,594,490	51,604,990	no_annotation				
event_6	chr N6	11,988,034	11,998,534	cytochrome b561 domain-containing protein At2g30890 [Elaeis guineensis]	chr N6	10,543,470	10,564,469	Pentatricopeptide repeat-containing protein At2g03380, mitochondrial
					chr N6	10,543,470	10,564,469	Cytochrome b561 domain-containing protein At4g18260 (Protein b561A.tha13)
					chr N6	10,543,470	10,564,469	Thioredoxin-like protein CITRX2, chloroplastic (EC 1.8.-.-) (Cf-9-interacting thioredoxin 2) (NbCiTrx2)
	chr N8	115,247,747	115,258,247	pentatricopeptide repeat-containing protein At2g13600-like [Elaeis guineensis]				
event_7	super-scaffold 54536	897,160	897,660	no_annotation				
	chr N9	83,098,044	83,108,544	no_annotation	chr N9	99,832,974	99,853,976	FHA domain-containing protein FHA2 (Protein FORKHEAD-ASSOCIATED DOMAIN 2) (AtFHA2)
event_8	chr N11	27,447,046	27,457,546	no_annotation				
	chr N7	91,097,284	91,107,784	pathogenesis-related genes transcriptional activator PTI6-like [Elaeis guineensis]	chr N7	115,841,908	115,862,929	no annotation
event_9	chr N11	29,228,454	29,238,954	no_annotation				
	chr N3	86,662,320	86,672,820	ubiquitin carboxyl-terminal hydrolase 26 isoform X1 [Elaeis guineensis]	chr N3	101,856,924	101,877,935	Ubiquitin carboxyl-terminal hydrolase 26 (EC 3.4.19.12) (Deubiquitinating enzyme 26)
event_10	chr N3	120,207,648	120,218,148	profilin isoform X2 [Elaeis guineensis]	chr N3	133,183,951	133,204,949	Mediator of RNA polymerase II transcription subunit 20a
					chr N3	133,183,951	133,204,949	Profilin (Minor allergen Lit c 1) (allergen Lit c 1)
	chr N10	6,765,695	6,776,195	no_annotation				
event_11	chr N3	97,449,932	97,460,432	no_annotation				
	chr N11	105,654,247	105,664,747	hypothetical protein C4D60_Mb06t12470 [Musa balbisiana]	chr N11	107,744,284	107,765,291	no annotation
event_12	chr N3	97,409,145	97,419,645	type 4 nonspecific lipid transfer protein LTP401 [Elaeis guineensis]				
	chr N3	97,409,145	97,419,645	non-specific lipid-transfer protein-like protein At5g64080 isoform X1 [Elaeis guineensis]				
	chr N3	97,409,145	97,419,645	inositol-3-phosphate synthase [Phoenix dactylifera]				
	chr N4	135,474,118	135,484,618	hippocampus abundant transcript-like protein 1 isoform X1 [Elaeis guineensis]	chr N4	120,791,617	120,812,626	24-methylenesterol C-methyltransferase 2 (24-sterol C-methyltransferase 2) (Sterol-C-methyltransferase 2) (EC 2.1.1.143)
event_13	chr N2	105,536,422	105,546,922	uncharacterized protein LOC105046218 [Elaeis guineensis]	chr N2	150,405,631	150,426,632	AP-1 complex subunit mu-2 (Adaptor protein complex AP-1 subunit mu-2) (Protein HAPLESS 13)
	chr N7	2,639,945	2,650,445	beta-glucuronosyltransferase GlcAT14A isoform X1 [Elaeis guineensis]				
event_14	chr N2	110,803,960	110,814,460	E3 ubiquitin-protein ligase BOI isoform X1 [Elaeis guineensis]				
	chr N11	115,491,865	115,502,365	LOW QUALITY PROTEIN: uncharacterized protein LOC105054004 [Elaeis guineensis]	chr N11	117,217,860	117,238,863	Asparagine--tRNA ligase, chloroplastic/mitochondrial (EC 6.1.1.22) (Protein OVULE ABORTION 8)
event_15	Super-Scaffold_82001	7,753,392	7,763,392	no_annotation				
	chr N4	58,649,925	58,660,425	no_annotation	chr N4	46,937,636	46,958,612	No protein found
event_16	Super-Scaffold_82001	7,778,066	7,788,066	no_annotation				
	chr N4	58,674,599	58,685,099	no_annotation	chr N4	46,962,366	46,983,381	No protein found
event_17	chr N2	21,149,044	21,159,544	60S ribosomal protein L21-1 [Elaeis guineensis]	chr N2	23,768,327	23,789,297	60S ribosomal protein L21-1
	chr N16	45,409,215	45,419,715	FAD synthase isoform X2 [Elaeis guineensis]				
event_18	chr N2	21,799,919	21,810,419	no_annotation				
	chr N16	45,223,575	45,234,075	tetraspanin-18 isoform X2 [Elaeis guineensis]	chr N16	47,624,669	47,645,684	no_annotation

PacBio subreads generated for EO12.1 were aligned to both EO12.1 and EG11 and used to identify candidate translocations. EO12.1 was considered the reference with translocation predicted in EG11 (Methods). PCR support was obtained for 18 of 32 predicted translocations. Because translocations may impact neighboring genes even if the coding region of the gene is not translocated, sequences including the regions of both genomes, plus 10 Kb of sequence flanking the region, were annotated based on mapping of sequences to each assembly, and annotations within these sequence windows are reported. Multiple entries for a given event/assembly indicate more than one annotation within the sequence window. For example, event_1 indicates that a region of EO12.1 chromosome N13 within 10 Kb of gene “Alternative NAD(P)H-ubiquinone oxidoreductase” has been translocated in EG11 to Chr N9, and the translocation insertion in N9 is within 10 Kb of gene “uncharacterized protein LOC103703199”. The remaining EG11 Chr N13 sequence retains coding sequences of the “Alternative NAD(P)H-ubiquinone oxidoreductase” gene. Event_2 represents a subtly different type of translocation. Event_2 indicates that a region of EO12.1 on Chr N9 within 10 Kb of genes “TIC 32” and “ZHD2” has been translocated in EG11 to Chr N14. In this example, coding sequence of “ZHD2” was carried with the translocation to Chr N14. The removal of the translocated sequencing from EG11 Chr N9 results in a different gene, “AKT2”, being within 10 Kb of the Chr N9 translocation breakpoints.

Table S6. Structural variants predicted by SYRI comparisons of EG11 and EO12.1

Variant Type	Eg_Chr	Eg_Start	Eg_End	Eg_size_bp	Eo_Chr	Eo_Start	Eo_End	Eo_size_bp
Inversion	Chr N1	66,876,366	71,838,272	4,961,906	Chr N1	66,801,310	80,165,547	13,364,237
Inversion	Chr N2	31,297,011	38,092,341	6,795,330	Chr N2	35,432,515	48,337,688	12,905,173
Inversion	Chr N3	55,370,162	57,654,485	2,284,323	Chr N3	43,042,833	52,743,659	9,700,826
Inversion	Chr N4	5,015	8,342,407	8,337,392	Chr N4	6,328	6,670,881	6,664,553
Inversion	Chr N6	47,525,087	52,675,944	5,150,857	Chr N6	41,574,119	46,753,959	5,179,840
Inversion	Chr N11	51,918,037	58,375,147	6,457,110	Chr N11	52,688,360	57,746,225	5,057,865
Inversion	Chr N12	36,703,790	41,705,426	5,001,636	Chr N12	43,570,263	45,960,463	2,390,200
Inversion	Chr N15	8,189,898	15,608,252	7,418,354	Chr N15	1,885,504	10,148,141	8,262,637
Duplication in EG11	Chr N4	112,977,071	113,038,679	61,608	Chr N4	81,982,679	82,036,718	54,039
Duplication in EG11	Chr N10	8,447,075	8,541,823	94,748	Chr N10	2,638,993	2,734,680	95,687
Inverted Duplication in EG11	Chr N13	29,121,479	29,247,303	125,824	Chr N13	31,513,323	31,643,283	129,960
Duplication in EG11	Chr N15	68,694,818	68,741,144	46,326	Chr N15	61,580,199	61,654,351	74,152
Duplication in EO12.1	Chr N7	49,270,354	49,346,205	75,851	Chr N7	28,550,163	28,603,407	53,244
Inverted Translocation	Chr N3	118,907,573	118,985,608	78,035	Chr N10	7,641,338	7,695,952	54,614
Inverted Translocation	Chr N3	118,925,638	119,021,642	96,004	Chr N10	7,609,932	7,683,944	74,012
Inverted Translocation	Chr N3	119,106,253	119,161,562	55,309	Chr N10	7,481,759	7,538,878	57,119
Inverted Translocation	Chr N6	37,716	92,199	54,483	Chr N8	158,658,835	158,703,023	44,188
Inverted Translocation	Chr N6	53,549	108,858	55,309	Chr N8	158,631,679	158,686,424	54,745
Inverted Translocation	Chr N10	4,455,761	4,538,934	83,173	Chr N3	132,263,324	132,334,333	71,009
Inverted Translocation	Chr N10	4,730,057	4,780,593	50,536	Chr N3	132,041,695	132,101,516	59,821

Structural variants predicted by SYRI comparison of EG11 and EO12.1. Variant Type, type of predicted structural variant; Eg_Chr, EG11 pseudochromosome involved; Eg_Start, starting genome coordinate for EG11 predicted variant; Eg_End, ending genome coordinate for EG11 predicted variant; Eg_size_bp, total predicted EG11 variant size in base pairs; Eo_Chr, EO12.1 pseudochromosome involved; Eo_Start, starting genome coordinate for EO12.1 predicted variant; Eo_End, ending genome coordinate for EO12.1 predicted variant; Eo_size_bp, total predicted EO12.1 variant size in base pairs. Boxed rows indicate independent, yet overlapping SYRI variant predictions which likely represent a common structural variant. Variant are diagrammed in Fig. S8 and S9.

Table S7. Gene model annotation summary

<table>
<tr><td>Step</td><td></td><td></td><td colspan="3">EG11</td><td colspan="3">EO12.1</td></tr>
<tr><td>1</td><td rowspan="2"></td><td>Total Transcripts (Mikado and Maker)</td><td colspan="3">293,746</td><td colspan="3">250,045</td></tr>
<tr><td>2</td><td>Total Transcripts (After Removal of lncRNA)</td><td colspan="3">265,042</td><td colspan="3">224,915</td></tr>
<tr><td rowspan="6">3</td><td rowspan="3">BLAST</td><td>Blastp (RefSeq)</td><td>Hit
130,470</td><td colspan="2">No-hit
134,572</td><td>Hit
105,945</td><td colspan="2">No-hit
118,970</td></tr>
<tr><td>Blastp (nr)</td><td></td><td>Hit
17,575</td><td>No-hit
116,997</td><td></td><td>Hit
16,121</td><td>No-hit
124,725</td></tr>
<tr><td>GO mapping</td><td colspan="3">93,630</td><td colspan="3">72,617</td></tr>
<tr><td>InterProScan</td><td>InterProScan hit</td><td colspan="3">217,282</td><td colspan="3">185,891</td></tr>
<tr><td>EggNOG</td><td>EggNOG hit</td><td colspan="3">50,871</td><td colspan="3">40,206</td></tr>
<tr><td>Merged Annotation</td><td>Total transcripts with GO
Total transcripts with EC</td><td colspan="3">101,123 (44,861 genes)
46,348 (17,833 genes)</td><td colspan="3">76,846 (36,817 genes)
35,790 (17,731 genes)</td></tr>
<tr><td>4</td><td colspan="2">Total transcripts and genes with GO, plus transcripts without GO but have at least 2 independent support evidences (BLASTP, CAGE,RNA-seq) data.</td><td colspan="3">105,594 (46,740 genes)</td><td colspan="3">80,631 (38,829 genes)</td></tr>
<tr><td>5</td><td colspan="2">Final selected set of transcripts and gene models after selected manual curation</td><td colspan="3">105,560 (46,697 genes)</td><td colspan="3">80,520 (38,658 genes)</td></tr>
</table>

Table S8. Gene model prediction summary

Class	Supporting Data			EG11		EO12.1	
	CAGE	Expression	BLAST	No. Transcripts	Cummulative No. Genes	No. Transcripts	Cummulative No. Genes
1	Yes	Yes	Yes	25,408	7,256	19,456	7,332
2	Yes	Expression or BLAST		2,359	7,925	374	7,563
3	-	Yes	Yes	45,311	21,404	43,098	22,256
4	CAGE or Expression		-	4,972	25,884	6,296	27,840
5	-	-	Yes	9,952	29,376	1,737	29,140
6	-	-	-	17,558	46,697	9,559	38,658
Total				**105,560**	**46,697**	**80,520**	**38,658**

Table S9. Primer pairs for amplification of retrotransposon probes used in the Fluorescence *in situ* hybridization (FISH) analyses

Retrotransposon	Primer (Forward)	Primer (Reverse)	Reference
Gypsy	MRNATGTGYGTNGAYTAYMG	RCAYTTNSWNARYTTNGCR	62
LINE	RVNRANTTYCGNCCNATHAG	GACARRGGRTCCCCCTGNCK	63
Copia	CCATATGGGTTGGTTGTCC	ACAGCGACTCATTCTTCTCC	64
